# Supplementary material for: Maternal anemia and underweight as determinants of pregnancy outcomes: cohort study in eastern rural Maharashtra, India
Source: BMJ Open. 2018 Aug 8;8(8):e021623. doi: 10.1136/bmjopen-2018-021623 (PMC6089300; doi:10.1136/bmjopen-2018-021623)
Supplement: Author's manuscript [file bmjopen-2018-021623.draft_revisions.pdf]

BMJ Open

BMJ Open is committed to open peer review. As part of this commitment we make the peer review history of every article we publish publicly available.

When an article is published we post the peer reviewers' comments and the authors' responses online. We also post the versions of the paper that were used during peer review. These are the versions that the peer review comments apply to.

The versions of the paper that follow are the versions that were submitted during the peer review process. They are not the versions of record or the final published versions. They should not be cited or distributed as the published version of this manuscript.

BMJ Open is an open access journal and the full, final, typeset and author-corrected version of record of the manuscript is available on our site with no access controls, subscription charges or pay-per-view fees (http://bmjopen.bmj.com).

If you have any questions on BMJ Open's open peer review process please email info.bmjopen@bmj.com

BMJ Open

MATERNAL ANEMIA AND UNDERWEIGHT AS DETERMINANTS OF PREGNANCY OUTCOMES: COHORT STUDY IN RURAL MAHARASHTRA, INDIA

Journal:	*BMJ Open*
Manuscript ID	bmjopen-2018-021623
Article Type:	Research
Date Submitted by the Author:	10-Jan-2018

Complete List of Authors:	Patel, Archana; Lata Medical Research Foundation, Nagpur<br>Prakash, Amber; Lata Medical Research Foundation, Nagpur, Research Unit<br>Das, Prabir Kumar; Lata Medical Research Foundation, Nagpur<br>Gupta, Swarnim ; Lata Medical Research Foundation, Nagpur<br>Hibberd, Patricia; Boston University School of Public Health; Boston University School of Medicine

Keywords:	Anaemia < HAEMATOLOGY, PUBLIC HEALTH, Community child health < PAEDIATRICS

SCHOLARONE™
Manuscripts

For peer review only

MATERNAL ANEMIA AND UNDERWEIGHT AS DETERMINANTS OF PREGNANCY OUTCOMES: COHORT STUDY IN RURAL MAHARASHTRA, INDIA

Archana Patel[1], Amber Abhijeet Prakash*[1], Prabir Kumar Das[1], Swarnim Gupta[1], Patricia Hibberd[2]

1. Lata Medical Research Foundation, Nagpur, India

2. Boston University School of Public Health and Boston University School of Medicine, Boston, United Stated of America

First Author:

Archana Patel (MD PhD)

Program Director

Address: Lata Medical Research Foundation,

Vasant Nagar, Nagpur- 440022,

Maharashtra, India

Telephone: +0712-2249569

Email: dr_apatel@yahoo.com

*Second and Corresponding Author:

Amber Abhijeet Prakash (MSc)

Senior Statistician and Data Manager

Address: Lata Medical Research Foundation,

Vasant Nagar, Nagpur- 440022,

For peer review only

Maharashtra, India

Telephone: +0712-2249569

Email: rockman.blues21@gmail.com

Third Author:

Prabir Kumar Das (MD)

Country Coordinator

Address: Lata Medical Research Foundation,

Vasant Nagar, Nagpur- 440022,

Maharashtra, India

Telephone: +0712-2249569

Email: prabir_das23@rediffmail.com

Fourth Author:

Swarnim Gupta (PhD)

Nutrition Consultant

Address: Lata Medical Research Foundation,

Vasant Nagar, Nagpur- 440022,

Maharashtra, India

Telephone: +0712-2249569

Email: gupta.swarnim@gmail.com

For peer review only

Fifth Author:

Patricia L Hibberd (MD PhD)

Chair, Department of Global Health

Professor of Global Health, Boston University School of Public Health

Professor of Medicine, Boston University School of Medicine

372 Crosstown Building

801 Massachusetts Avenue

Boston, MA 02118

Email: plh0@bu.edu

Telephone: 617-414-1260

WORD COUNT: 5,147

COPYRIGHT

The Corresponding Author has the right to grant on behalf of all authors and does grant on behalf of all authors, an exclusive licence (or non exclusive for government employees) on a worldwide basis to the BMJ Publishing Group Ltd to permit this article (if accepted) to be published in BMJ editions and any other BMJPGL products and sublicences such use and exploit all subsidiary rights, as set out in our licence

For peer review only

ABSTRACT

Objectives: To study the trend in the prevalence of anemia and low BMI pregnant women from Central India and evaluate if low BMI and anemia have any effects on pregnancy outcomes.

Design: Prospective observational cohort study.

Setting: Catchment areas of 20 rural primary health centers in 4 districts of Maharashtra state (central India)

Participants: 72,750 women from the Nagpur site of Maternal and Newborn Health Registry of Global Network, enrolled from 2009 to 2016.

Main outcome measures: Mode of delivery, pregnancy related complications at delivery, stillbirths, neonatal deaths and low birth weight in babies.

Results: Over 90% of the women included in the study were anemic and about a third were underweight (BMI<18 kg/m^2). Any degree of anemia was found to increase the risk of having stillbirths neonatal deaths and LBW babies significantly ($p<0.05$). The risks were higher among the moderate to severely anemic women than those with mild anemia for post natal outcomes (relative risk; 95% confidence interval (RR;(95% CI)) for stillbirth (1.4;(1.2 - 1.8) vs. 1.3;(1.1 - 1.6)); for neonatal deaths (1.7;(1.3 - 2.1)vs.1.3;(1.0 - 1.6)); for LBW (<2500gms) (1.3;(1.2 - 1.4) vs. 1.1;(1.0 - 1.2)). Underweight BMI was found to be a significant risk for neonatal deaths

(1.1;(1.0 - 1.3)) and having LBW babies (1.2;(1.2 - 1.3)). The risk of having stillbirths (1.5;(1.2 - 1.8)), neonatal deaths (1.7;(1.3 - 2.3)) and LBW babies (1.5;(1.4 - 1.6)) was highest when the anemia and underweight co-existed in the included women. Obesity/overweight during pregnancy increased the risk of maternal complications at delivery (1.6;(1.5 - 1.7)) and of cesarean section (1.5;(1.4 - 1.6)).

Conclusions: Anemia and underweight in pregnancy were individually associated with enhanced risk of stillbirths, neonatal deaths and LBWs. This risk increased further if these anemia and underweight were present simultaneously.

Trial registration: Registered at ClinicTrials.gov (NCT01073475)

Keywords: Body Mass Index, anemia, pregnancy, neonatal deaths, malnutrition, stillbirth, low birth weight

For peer review only

STRENGTHS AND LIMITATIONS

Strengths of the study

1. ***Large sample and data quality***: The biggest strength of the present study is its prospective nature and large sample size. Most of the studies conducted in the target geographical region over the years have reported their finding on much smaller sample sizes. This study not only reports findings based on a larger number of participants but is also robust in the quality of

data it uses, due to rigorous and continuous monitoring of the collected data conforming to international standards.

2. ***Synergistic effects:*** Although there are number of studies to show effects of BMI or anemia on birth outcomes independently and also some evidence that anemia is associated with low BMI, none of these studies reports the synergistic effects of both these indicators on poor maternal outcome at delivery and post-partum comprehensively. To best of our knowledge the study is the first, with more than 72000 subjects to report independent and synergistic effects of maternal anemia and low BMI on complications at delivery, experiencing cesarean sections, and on poor outcomes- still birth, neonatal death and delivering low birth weight babies.
3. ***Policy implications:*** The findings of the study may have policy implications at national level or in low middle income countries that have similarities with India in term of the maternal and child nutritional status indicators.

Limitations of the study

1. ***Dietary practices:*** We believe that micronutrient deficiency was the major cause of anemia, we were not able to conclusively determine it or determine other possible causes such as hemoglobinopathies, worm infestations and malaria. We did not collect the data on dietary intake consumption of iron folic acid (IFA) supplement and food supplements through the government scheme. This additional data could have helped in understanding the etiology better and verifying our speculations. Nevertheless, national survey shows a poor intake of this nutrients and low compliance in general by the rural pregnant women of Maharashtra, India.

For peer review only

2. ***Gestational age and pre-pregnancy data:*** We did not have accurate data on gestational age to evaluate if anemia and BMI independently or together had any relationship with preterm births which is recognized as a major cause of neonatal mortality. We also did not have pre-pregnancy BMI and anemia status, which may have underestimated the BMI prevalence. However, we do not expect any large variation due to the reasons that we attempted to take anthropometry measurements as early as possible and within first trimester when there is not much weight gain and the appetite is poor because of gastritis.

For peer review only

INTRODUCTION

Malnutrition is a serious underlying cause for child and maternal deaths around the globe. Undernutrition during pregnancy restricts fetal growth which contributes to about 800,000 neonatal and 400,000 post-neonate deaths among infants, and 20% of the stunting in the first 2 years of life. Short stature of mothers and maternal anemia increase the risk of death of the mother at delivery, and accounts for at least 20% of maternal deaths.[1-3] Because of the crucial role in survival and well being of both the mother and child, Sustainable Development Goals (SDG) bring explicit attention to nutrition in the 2nd element of the 17 point agenda. World Health Assembly also has set 6 Global Nutrition Targets for 2025, out of which one is specifically for women of reproductive age.[4-5] Since India has largest number of neonatal, infant and children under 5, mortality and growth faltering,[6-8] understanding the maternal nutritional status as a determinant may have strategic implications.

The two important indicators for assessing maternal malnutrition are Body Mass Index (BMI) and prevalence of anemia, both of which can affect health of a mother and her fetus. Complications associated with maternal overweight and obesity (BMI>25 kg/m^2) encompass increased incidence of gestational hypertension, pre-eclampsia, macrosomia, early induction of labor and caesarian deliveries. These concerns are, at present, no more restricted to only high income countries. With a large rightward shift in the BMI distributions, there is perturbation among low middle-income countries as well.[9] However, in the rural Indian scenario where undernutrition is still predominant, low BMI and the associated increased risk of LBW and preterm deliveries require greater attention. Maternal anemia, as a result of undernutrition and

For peer review only - http://bmjopen.bmj.com/site/about/guidelines.xhtml

infections has been found to coexist with low BMI. This co-existence among the Indian women of reproductive age is reflected in findings of the recent National Family and Health Survey (NFHS).In the survey, approximately one-fourth (22.9%) of the females were reported to have a BMI less than 18.5 kg/m^2 and 53% were reported as anemic. The percentage of low BMI further increases for rural areas of the Maharashtra state.[10]

Anemia has been found to be associated with post-partum hemorrhage, LBW, small for gestational age babies and perinatal death.[11,12] Yet the information on pregnancy and neonatal outcomes in women who suffer from the dual burden of low BMI and anemia is fragmentary. Understanding the extent of risk arising out of this synergy specifically in the context of adverse pregnancy outcomes prior to the onset of labor may guide further referrals and admissions to health facility equipped to manage pregnancy complications and preterm babies until the long-term goal of eradicating undernutrition is achieved. Further, any increased risk associated with undernutrition, may also suggest a targeted approach for improving the nutritional status of this segment of the population. We used data from an ongoing Maternal and Newborn Health Registry (MNHR) based in rural areas of Nagpur, Maharashtra, India to (1) study the prevalence and trends of maternal anemia and low BMI from 2009 to 2016 (2) describe the maternal demographic characteristics associated with different levels of maternal hemoglobin and BMI from 2009-2016, (3) evaluate whether low BMI and anemia were independent or synergistic risk factors for adverse pregnancy outcomes that occurred from year 2009 to 2016, (4) investigate if co-existence of low BMI and anemia had any augmentative affect on poor birth outcomes.

For peer review only

METHODS

Study design and settings

The present study utilizes data collected from the Nagpur, India site of MNHR, which is a population based registry established in 2009 by the Global Network (GN) with support from *Eunice Kennedy Shriver* National Institute of Child Health and Human Development (NICHD), United States of America (USA).The MNHR is an observational, prospective cohort operating in seven different sites around the world including two, in India. The Nagpur site comprises of 20study clusters from the rural areas of central India in the state of Maharashtra. Geographical areas surrounding government Public health centers (PHC) with approximately 900 deliveries per year, constitute a study cluster. The MNHR aims to recruit all consenting women residing within the clusters, as early as possible during their pregnancy and then to follow them until 42 days post-partum. The detailed methods followed by the MNHR have been published elsewhere. [13] The time period of this study was from 2009 to 2016.

Participants

The participants in this study, were all consenting pregnant women belonging to the clusters of the Nagpur site, enrolled within the study period, with completed data collection at the time of analyses. Exclusions comprised of women with medical termination of pregnancy (MTP) (premature expulsion of a non-viable fetus before 20 weeks gestation) or miscarriages or multiple fetuses or extreme/missing values for one or more study variables of interest. Early enrollment of the participants was ensured through active surveillance in the study clusters by Nagpur site MNHR public health staff. The enrolled women were followed through to a

For peer review only

perinatal visit after delivery (within a week) and to a follow up visit around 42 days after birth. A variety of maternal and infant data on study variables and outcomes, were recorded for the enrollees.

Data collection procedures

Briefly, data were collected by trained registry administrators (RA) via standardized forms. RAs included medical officers and auxiliary nurse-midwives (ANM) employed at Primary Health Centers (PHCs) and sub-centers. A two day initial training for data collection was provided to the Registry Administrators (RAs). This was followed by a refresher training every six months, and unscheduled special trainings as per need. The RAs returned filled in forms which were manually scrutinized for errors prior to data entry at Lata Medical Research Foundation (LMRF), Nagpur. After entry, data were transmitted to the Global Network (GN) data management center (Research Triangle Institute, Durham, North Carolina, USA) where digital data checks were performed on a monthly basis. The data management center forwarded edit, monitoring and performance reports to the Nagpur site team for data cleaning and management. The data were continually reviewed for quality by *Eunice Kennedy Shriver* National Institute of Child Health and Human Development (NICHD) appointed data monitoring committee and by the Nagpur site data collection team

Study variables

At the first antenatal visit, information regarding the enrolled women's, characteristics namely age, education, parity, height, weight and hemoglobin was recorded. Weight was measured to nearest 100grams using a spring balance and height to nearest centimeter using a non-flexible

For peer review only

measuring tape fixed to a wall. Body mass index (BMI) was calculated by dividing weight in kilograms (kg) by squared height in meters. Standard World Health Organization (WHO) BMI classifications [14] were used to categorize the enrollees as underweight (BMI<18.5 Kg/m^2), normal (BMI 18.5-24.9 Kg/m^2), or as overweight/ obese (BMI≥25Kg/m^2). Hemoglobin was estimated within two weeks of enrollment using Sahli's method.[15]A concentration of hemoglobin (Hb) level < 11 g/dL indicated presence of anemia. Based on WHO classification [16] hemoglobin readings were used to define the severity of anemia as normal (>=11g/dL), mild (10<11 g/dL) and moderate/severe (<10 g/dL).A BMI-anemia factor variable at 4 levels was defined using combinations of hemoglobin<11(g/dL) or not and BMI<18.5 (Kg/m^2) or not. Thus the 4 levels of the anemia-BMI factor variable were 'anemic-normal', 'anemic-underweight', 'non anemic-normal' and 'non anemic-underweight'. Mother's age was recorded in years completed at the time of enrolment. An education of up to 4 grades was considered 'primary', from grade 5 to grade 10 as 'secondary' and more than grade 10 as 'university'. Parity was recorded as whole numbers zero or more, depending on if the woman had been pregnant before enrollment or in pregnancy had carried a fetus to 20 weeks of gestation, or not.

Study Outcomes

The impact of variations in levels of anemia and categories of BMI was assessed on rates of 5 study outcomes including mode of delivery, any pregnancy related complications at delivery, stillbirths, neonatal deaths and low birth weight (LBW) in babies. We did not assess the determinants of maternal mortality as the numbers of mortality were small, thus restricting any modeling analysis. However, we described the causes of death with respect to the level of anemia and BMI status. Mode of delivery was recorded as cesarean section (CS) or non-

For peer review only

cesarean (vaginal, assisted vaginal).Any pregnancy related complications at delivery were recorded as presence of one or more of the following; obstructed/prolonged labor, severe antepartum hemorrhage (APH), severe postpartum hemorrhage (PPH), hypertension, pre-eclampsia, and oblique/abnormal lie. Birth outcomes such as stillbirth, neonatal deaths (deaths of neonates from birth to 28 days of life) and LBW rates were assessed. Stillbirth rates were calculated as number of stillbirths per 1000 total births while neonatal death rates were calculated as number of neonatal deaths per 1000 live births. Birth weight was measured by using either a pan spring or a pan digital weighing scale. Infants were classified as LBW if their birth weight was found to be less than 2500 grams. Maternal deaths along with their most likely reasons were recorded according to the time of their occurrence either at the perinatal period or at the follow up visit around 42 days post partum.

Statistical Analyses

Data were analyzed using the STATA 13.1 statistical package (Stata Corp, College Station, Texas, USA). Study variables of interest and their combinations, with years of enrollment were cross tabulated in Stata and were transformed to graphs either in Stata itself or in Microsoft Excel. Pearson's correlation coefficient was used to study correlation between hemoglobin concentrations and BMI values and was tested for significance. A univariate multinomial logistic regression, adjusting for clustering was used to identify association between the WHO levels of anemia and BMI categories. Significance of trends over the progression of years of enrollment in any of the study variables was determined using Cuzick's non parametric test [17] for trend across ordered groups. The year-wise rates of the outcomes (CS, pregnancy related complication and LBW) were determined for each level of anemia and category of BMI. For e.g. the rates of

For peer review only

LBW in women with moderate/severe anemia in year 2009 was–(number of women who delivered a LBW/number of women who had moderate/severe anemia) x 100. Similarly for stillbirths and neonatal deaths the rates were calculated per 1000 total births and per 1000 live births respectively. The risks of association of maternal demographic characteristics and study outcomes were studied using multivariable regression analyses solved with generalized estimating equations (GEE), adjusting for clustering within the PHCs. For the first set of regression models, explanatory variables included demographic characteristics - mother's age, education level and parity, along with levels of anemia and categories of BMI, as individual variables while dependent variables included all 5 study outcomes. A second set of models used demographic characteristics along with the anemia-BMI factor variable as explanatory and modeled 3 post natal outcomes - stillbirths, neonatal death and LBW. Type I error rates of 0.05 (significant) and 0.001 (highly significant) were used to test the statistical relevance of trends as well as the association between factors and outcomes.

RESULTS

Participants

Of the 79,707 pregnant women enrolled in the MNHR during the study period, 6,957 were excluded, leaving 72,750 women eligible for analyses. The reasons for exclusion were no pregnancy outcomes yet (1,781/6,957, 25.6%), miscarriage/MTP/multiple gestation (4412/6,957, 63.4%) and invalid or missing data on BMI or hemoglobin (764/6957, 11%).

For peer review only

Baseline maternal characteristics

A vast majority of the included women was found to be anemic (65811/72750, 90.5%) and over a third were underweight (25571/72750, 35.1%) (Table 1).A casual but highly significant correlation(r= 0.2; P<0.001) between hemoglobin concentrations and BMI values was observed. A multinomial logistic regression with normal hemoglobin concentrations as reference, showed highly potent (P<0.001) associations between underweight women and moderate/severe anemia (Risk Ratio (RR):1.9, 95% Confidence Interval (CI):1.6-2.2) as well as mild anemia (RR:1.3, 95% CI:1.2-1.5).The included women were mostly of young age, that is from 20 to 29 years (93%) and were nearly even in terms of parity (primiparous, 48% vs. multiparous, 52%). There was a significant rise in the proportion of women in age group >29 over the years of enrolment, and those who attained higher level of education in later years (2015-2016). For parity, there was a reduction in proportions of parity>2 in 2016 (Table 1). The levels of anemia showed no noticeable or significant trends over the progression of enrollment years. The proportions of underweight (28% to 38%) and overweight/obese (3.5% to 5.5%) women showed a casual increasing trend over years of enrollment. The levels of anemia displayed a nearly homogenous trend distribution over categories of BMI and year of enrollment (Figure 1). The BMI-anemia factors variable showed a highly significant (P<0.001) trend, over the years of enrollment. Anemic women showed a rise in proportion when coupled with the underweight BMI class (25% to 35%) and a decline in proportion on coupling with the normal BMI class (63% to 53%,) (Figure 2). Non anemic combinations with the same BMI classes did not exhibit an identifiable trend over years of enrollment.

For peer review only

Table 1: Baseline Characteristics of women in the study, by year of enrollment

Characteristics	Years								
	**2009**	**2010**	**2011**	**2012**	**2013**	**2014**	**2015**	**2016**	**Total**
	N (%)	N (%)	N (%)	N (%)	N (%)	N (%)	N (%)	N (%)	N (%)
	6433 (100)	10447 (100)	9626 (100)	9905 (100)	9496 (100)	9454 (100)	10108 (100)	7281 (100)	72750 (100)
Mother's Age (years)*									
<20	131 (2)	209 (2)	181 (1.9)	188 (1.9)	190 (2)	193 (2)	209 (2.1)	157 (2.2)	1458 (2)
20-29	6044 (94)	9867 (94.4)	9056 (94.1)	9269 (93.6)	8834 (93)	8743 (92.5)	9266 (91.7)	6640 (91.2)	67719 (93.1)
>29	258 (4)	371 (3.6)	389 (4)	448 (4.5)	472 (5)	518 (5.5)	633 (6.3)	484 (6.6)	3573 (4.9)
Mother's Education (grades)*									
Primary or Less (<=4)	1476 (22.9)	2281 (21.8)	1846 (19.2)	1927 (19.5)	1882 (19.8)	693 (7.3)	633 (6.3)	329 (4.5)	11067 (15.2)
Secondary(5-10)	3814 (59.3)	6210 (59.4)	5818 (60.4)	5880 (59.4)	5432 (57.2)	4962 (52.5)	5101 (50.5)	3522 (48.4)	40739 (56)
University(>10)	1139 (17.7)	1956 (18.7)	1958 (20.3)	2071 (20.9)	2172 (22.9)	3768 (39.9)	4358 (43.1)	3415 (46.9)	20837 (28.6)
Missing	4 (0.1)	0 (0)	4 (0)	27 (0.3)	10 (0.1)	31 (0.3)	16 (0.2)	15 (0.2)	107 (0.1)
Parity*									
Nulliparous	2905 (45.2)	5118 (49)	4706 (48.9)	4817 (48.6)	4384 (46.2)	4530 (47.9)	4967 (49.1)	3598 (49.4)	35025 (48.1)
1-2	3300 (51.3)	5032 (48.2)	4669 (48.5)	4827 (48.7)	4907 (51.7)	4721 (49.9)	4888 (48.4)	3544 (48.7)	35888 (49.3)
>2	228 (3.5)	297 (2.8)	249 (2.6)	261 (2.6)	204 (2.1)	197 (2.1)	234 (2.3)	137 (1.9)	1807 (2.5)
Missing	0 (0)	0 (0)	2 (0)	0 (0)	1 (0)	6 (0.1)	19 (0.2)	2 (0)	30 (0)
Anemia (gm/dl)									
Moderate/Severe(<10)	2962 (46)	5283 (50.6)	4620 (48)	4730 (47.8)	4682 (49.3)	4418 (46.7)	5058 (50)	3457 (47.5)	35210 (48.4)
Mild(10<11)	2743 (42.6)	4341 (41.6)	4033 (41.9)	4337 (43.8)	4006 (42.2)	4152 (43.9)	4041 (40)	2948 (40.5)	30601 (42.1)
Normal(>=11)	728 (11.3)	823 (7.9)	973 (10.1)	838 (8.5)	808 (8.5)	884 (9.4)	1009 (10)	876 (12)	6939 (9.5)
Body Mass Index (kg/m^2) *									
Underweight(<18.5)	1784 (27.7)	3537 (33.9)	3391 (35.2)	3450 (34.8)	3411 (35.9)	3424 (36.2)	3786 (37.5)	2788 (38.3)	25571 (35.1)
Normal(18.5-24.9)	4426 (68.8)	6558 (62.8)	5931 (61.6)	6061 (61.2)	5699 (60)	5619 (59.4)	5789 (57.3)	4095 (56.2)	44178 (60.7)
Overweight or Obese(≥25)	223 (3.5)	352 (3.4)	304 (3.2)	394 (4)	386 (4.1)	411 (4.3)	533 (5.3)	398 (5.5)	3001 (4.1)

* Highly significant (P<0.001) non parametric trend over years of enrollment

For peer review only - http://bmjopen.bmj.com/site/about/guidelines.xhtml

Maternal Mortality

The total number of maternal deaths was 29. Severe anemia was present in 20 women. Moderate/Severe anemia was identified as a primary cause of death in 1 and as a co-morbid condition in remaining 19. Of the total 29 deaths, anemia and underweight was observed in 8, only anemia in 19, only underweight in 1, and overweight/obese in 2. Reasons for maternal deaths, were hemorrhage (n=9, 31%), preeclampsia/eclampsia (n=7, 24%), infection (n=6, 21%), other (n=6, 21%) and missing(n=1, 3%).

Rates of study outcomes by years of enrollment

Overall(across all years), the rates of women experiencing CS or pregnancy related complications at delivery showed an increase with decreasing severity of anemia (Table 2). In contrast, overall rates of stillbirths per 1000 total births, neonatal deaths per 1000 live births and rates of LBW babies showed an increase with increasing severity of anemia. The rates of CS and pregnancy related complications increased with increasing BMI. The rates of stillbirths per 1000 total births, neonatal deaths per 1000 live births and rates of LBW babies showed an increase in underweight and decreased in overweight/obese mothers.

In particular, an increasing trend in women experiencing CS was observed for all levels of anemia as well as BMI, over the progression of years of enrollment. However, rates of pregnancy related maternal complications, stillbirths per 1000 births, neonatal deaths per 1000 live births and LBW babies did not show a discernable trend with the years of enrollment.

For peer review only

Table 2: Rates of study with respect to levels of anemia and body mass index categories, by year of enrolment

	Year of Enrollment								
	**2009**	**2010**	**2011**	**2012**	**2013**	**2014**	**2015**	**2016**	**Total**
**Variables**	**Rates of women experiencing cesarean sections**								
Anemia (gm/dl)									
Moderate/Severe(<10)	15.67	16.83	17.19	19.53	20.91	23.74	24.73	28.67	20.85
Mild(10-11)	16	17.97	19.69	21.51	24.84	26.42	31.08	33.58	23.8
Normal(>=11)	18.41	26.12	24.87	26.13	31.44	32.01	37.07	39.61	29.8
Body Mass Index (kg/m^2)									
Underweight(<18.5)	15.08	15.13	16.13	17.65	19.2	21.61	23.38	26.79	19.5
Normal(18.5-24.9)	15.95	18.65	19.91	21.53	24.64	26.87	29.9	33.41	23.6
Overweight or Obese(≥25)	27.8	35.8	33.55	41.12	43.78	43.55	49.72	53.52	42.59
**Variables**	**Rates of women with pregnancy complications present at delivery**								
Anemia (gm/dl)									
Moderate/Severe(<10)	14.58	14.1	12.03	14.76	14.72	10.5	10.7	10.85	12.78
Mild(10<11)	14.51	14.67	14.95	15.75	17.05	13.97	11.9	12.48	14.49
Normal(>=11)	16.62	21.51	16.65	17.42	22.15	18.1	15.86	14.27	17.73
Body Mass Index (kg/m^2)									
Underweight(<18.5)	13.85	12.58	11.53	14.03	14.07	10.25	10.46	10.26	12.04
Normal(18.5-24.9)	14.91	15.46	14.37	15.38	17.07	13.54	11.99	12.43	14.48
Overweight or Obese(≥25)	19.73	28.41	25.66	28.17	25.39	22.38	17.26	18.34	22.93
**Variables**	**Stillbirth rate per 1000 total births**								
Anemia (gm/dl)									
Moderate/Severe(<10)	29.37	25.55	22.29	23.89	26.27	21.05	23.33	26.61	24.54
Mild(10-11)	27.34	24.65	20.58	19.37	23.71	19.27	18.81	15.6	21.11
Normal(>=11)	12.36	18.23	15.42	16.71	16.09	16.97	14.87	15.98	15.85
Body Mass Index (kg/m^2)									
Underweight(<18.5)	29.15	25.73	22.41	22.32	25.21	20.74	22.45	27.62	24.05
Normal(18.5-24.9)	25.76	24.7	19.9	20.13	24.21	19.93	19.52	17.34	21.5
Overweight or Obese(≥25)	22.42	11.36	23.03	30.46	18.13	12.17	20.64	10.05	18.33
**Variables**	**Neonatal death rate per 1000 live births**								
Anemia (gm/dl)									
Moderate/Severe(<10)	22.06	21.63	19.64	28.74	24.05	21.99	24.05	21.89	23.09
Mild(10<11)	17.54	16.08	15.44	16.74	19.03	18.26	17.72	16.47	17.15
Normal(>=11)	11.25	25.38	11.62	14.8	6.33	12.82	13.25	9.37	13.06
Body Mass Index (kg/m^2)									
Underweight(<18.5)	21.23	20.13	21.26	23.68	24.98	20.09	22.94	20.74	21.96
Normal(18.5-24.9)	18.19	19.28	14.14	21.86	18.13	19.25	19.04	16.42	18.38
Overweight or Obese(≥25)	13.95	20.53	27.68	15.96	13.37	17.54	17.54	18.13	17.97
**Variables**	**Rates of low birth weight (<2500 g) babies**								
Anemia (gm/dl)									
Moderate/Severe(<10)	17.42	17.87	17.51	18.46	19.14	19.04	20.42	22.65	19.01
Mild(10-11)	16.99	14.37	14.63	13.53	14.5	14.72	16.98	18.69	15.35
Normal(>=11)	14.15	15.43	12.85	11.22	10.4	12.9	16.45	16.32	13.78
Body Mass Index (kg/m^2)									
Underweight(<18.5)	19.79	18.63	17.9	19.59	20.14	19.28	21.61	22.74	19.92
Normal(18.5-24.9)	15.95	15.23	14.69	13.81	14.6	15.09	17.29	19.54	15.61
Overweight or Obese(≥25)	11.66	10.51	15.13	10.41	10.88	14.11	12.38	10.8	11.96

For peer review only

Study Outcomes: Regressions

The rates of CS and pregnancy related complications during delivery were significantly higher in non-anemic women as compared to those who were anemic. However, significantly higher risk of stillbirths, neonatal deaths and LBW was observed with increasing severity of anemia (Table 3). Similarly the risks of CS and pregnancy related complications during delivery were higher in overweight/obese women. The rates of neonatal deaths and LBW were significantly higher in underweight women (Table 3).

Mother's age - more than 29 years, education level - secondary or less, and nulliparity were in common, found to be highly significantly associated with stillbirths, neonatal deaths and LBW. Women < 20 years were less at risk for CS and pregnancy related complications. Women with lower levels of education (secondary or lesser level of education) were also significantly less likely to experience CS or pregnancy related complication. Nulliparous women had a higher risk of CS and twice as likely to experience pregnancy related complications at birth. Though a parity of more than two though lowered the risk of CS and pregnancy related complications, it increased the risk for neonatal deaths (Table 3). The risks of stillbirth, neonatal deaths and LBW were the highest when women were both anemic and underweight (Table 4).

For peer review only

Table 3: Risk factors for study outcomes, multivariable estimates

	Study Outcomes				
**Characteristics**	**Cesarean Sections<br>N=16693, 23%<br>RR(95% CI)^**	**Pregnancy related Maternal complications<br>N=10163, 14%<br>RR(95% CI)^**	**Stillbirths<br>N=1620, 2.2%<br>RR(95% CI)^**	**Neonatal deaths (within 28 days of birth)<br>N=1368, 1.9%<br>RR(95% CI)^**	**Low birth weight (<2500 gms)<br>N=12347, 17%<br>RR(95% CI)^**
Mother's Age (years)					
<20	0.80**(0.72 - 0.90)	0.83*(0.73 - 0.95)	0.88(0.62 - 1.25)	1.28(0.94 - 1.75)	1.04(0.92 - 1.16)
20-29	ref	ref	ref	ref	ref
>29	1.60**(1.50 - 1.70)	1.38**(1.26 - 1.50)	1.76**(1.45 - 2.12)	1.47**(1.18 - 1.83)	1.28**(1.18 - 1.38)
Mother's Education (grades)					
Primary or Less (<=4)	0.47**(0.44 - 0.50)	0.69**(0.64 - 0.74)	1.62**(1.38 - 1.89)	1.49**(1.26 - 1.77)	1.14**(1.08 - 1.21)
Secondary(5-10)	0.68**(0.66 - 0.70)	0.89**(0.85 - 0.93)	1.38**(1.22 - 1.57)	1.30**(1.14 - 1.49)	1.14**(1.09 - 1.19)
University(>10)	ref	ref	ref	ref	ref
Parity					
Nulliparous	1.38**(1.34 - 1.43)	2.00**(1.88 - 2.12)	1.31**(1.18 - 1.45)	1.48**(1.33 - 1.66)	1.35**(1.30 - 1.41)
1-2	ref	ref	ref	ref	ref
>2	0.45**(0.38 - 0.53)	0.76*(0.64 - 0.90)	1.22(0.93 - 1.62)	1.52*(1.14 - 2.04)	1(0.89 - 1.13)
Anemia (gm/dl)					
Severe/Moderate (<10)	0.87**(0.83 - 0.92)	0.89**(0.84 - 0.95)	1.43**(1.17 - 1.75)	1.67**(1.33 - 2.08)	1.26**(1.18 - 1.35)
Mild(10>11)	0.90**(0.86 - 0.94)	0.90*(0.85 - 0.96)	1.28*(1.05 - 1.57)	1.28*(1.02 - 1.60)	1.08*(1.01 - 1.15)
Normal(>=11)	ref	ref	ref	ref	ref
Body Mass Index (kg/m^2)					
Underweight(<18.5)	0.85**(0.82 - 0.88)	0.85**(0.81 - 0.88)	1.1(1.00 - 1.22)	1.12*(1.00 - 1.25)	1.21**(1.16 - 1.25)
Normal(18.5-24.9)	ref	ref	ref	ref	ref
Overweight or Obese(≥25)	1.57**(1.48 - 1.67)	1.48**(1.36 - 1.60)	0.92(0.70 - 1.20)	1.11(0.84 - 1.46)	0.84*(0.76 - 0.94)

* P<0.05, ** P<0.001

^ Obtained by multivariable GEE regression models for each outcome, separately

For peer review only

Table 4: Anemia-BMI factors for study outcomes, multivariable estimates

	Study Outcomes		
**Characteristics**	**Stillbirths**<br><br>**N=1620, 2.2%**<br>**RR(95% CI)^**	**Neonatal deaths (within 28 days of birth)**<br>**N=1368, 1.9%**<br>**RR(95% CI)^**	**Low birth weight (<2500 gms)**<br>**N=12347, 17%**<br>**RR(95% CI)^**
Mother's Age (years)			
<20	0.88(0.62 - 1.25)	1.28(0.94 - 1.75)	1.04(0.93 - 1.16)
20-29	ref	ref	ref
>29	1.75**(1.45 - 2.11)	1.48**(1.19 - 1.84)	1.27**(1.17 - 1.37)
Mother's Education (grades)			
Primary or Less (<=4)	1.64**(1.40 - 1.91)	1.52**(1.29 - 1.80)	1.16**(1.10 - 1.23)
Secondary(5-10)	1.40**(1.23 - 1.58)	1.32**(1.16 - 1.51)	1.15**(1.10 - 1.20)
University(>10)	ref	ref	ref
Parity			
Nulliparous	1.30**(1.18 - 1.44)	1.47**(1.31 - 1.64)	1.35**(1.29 - 1.40)
1-2	ref	ref	ref
>2	1.24(0.94 - 1.63)	1.56*(1.17 - 2.09)	1.02(0.91 - 1.15)
Anemia(gm/dl)-BMI(kg/m^2) factors			
Anemic(<11)-Normal(>=18.5)	1.30*(1.04 - 1.63)	1.54**(1.19 - 2.00)	1.22**(1.13 - 1.32)
Anemic(<11)-Underweight(<18.5)	1.47**(1.17 - 1.85)	1.74**(1.33 - 2.26)	1.49**(1.37 - 1.62)
Non-Anemic(>=11- Normal(>=18.5)	ref	ref	ref
Non-Anemic(>=11)-Underweight(<18.5)	0.96(0.62 - 1.48)	1.37(0.88 - 2.13)	1.37**(1.20 - 1.57)

* P<0.05, ** P<0.001

^ Obtained by univariate GEE regression models for each outcome, separately

For peer review only

DISCUSSION

Maternal malnutrition continues to be a silent emergency particularly in low middle income countries (LMICs) like India. We attempted to understand through present study, not only the trends in the prevalence of anemia and low BMI over the eight years but less understood relationship of the maternal BMI and presence of anemia with pregnancy related outcomes among rural women from central India. We found that more than 90% of the pregnant women were anemic and 35% were underweight (BMI< 18.5). It was alarming that the rates of anemia remained unchanged over eight years (2009-2016). The rates of underweight increased by 8% over eight years, but also there was an increase in the rates of overweight/obese from 2012 to 2016. Anemia and underweight during pregnancy were individually found to significantly increase the risk of stillbirths, neonatal deaths and LBW. The risk increased further if both conditions were present during pregnancy.

The NFHS data (NFHS-3 and NFHS-4) appears to be the best available estimate for comparing the above findings. However, NFHS reports a anemia prevalence rate of about 54% for 2005-2016 and a subtle reduction of about 9 percentage points in prevalence for Maharashtra between NFHS-3(58%) to NFHS-4 (49%).[10,18] Our estimates are even higher than the District Level Household Survey (DLHS-4, 2012-2013) which reported a rate of 70%among women aged 15-49 years.[19] The women in our study were young (20-29yrs), from both rural and backward tribal regions known for high prevalence of micronutrient deficiency specifically iron deficiency anemia and other causes of anemia such as sickle cell anemia, hookworm infestation and

For peer review only

malaria.[20-25]Since the data collected in this study, were not focused on explaining the reasons for prevalence of anemia, the aforementioned can only be speculated and not verified.

Previous studies from these regions have also observed similar rates of anemia. [26-29] Variations in rates of hemoglobin estimations between studies can also be attributed to methods used for surveying and assessing hemoglobin levels.[30-31] The overall rates of anemia in pregnant women remain unchanged over eight years although there was some reduction in rates of severe anemia (Figure 1). This could be in part due to irregular supply and poor coverage of the national iron supplementation program [32-34] but also because of low compliance. The national level surveys show the compliance of merely 30% among the pregnant women [10,35] for which the side effects, forgetfulness, lack of awareness and false beliefs appear to be the major reasons.[36-38]Directly observed iron therapy seems promising in improving the compliance and is being implemented for adolescent girls.[39,40] Feasibility of extending such interventions along with intensive counseling to the pregnant women should be tested for improving the compliance.

Our study reports over a third of the study participants to be underweight during pregnancy which is consistent with the findings of NFHS for rural Nagpur.[10]Further, we also reported a significant rise of about 8% in the prevalence of low BMI from 2009-16. Present study also revealed an increasing shift in BMI from normal to overweight/obese categories over the years of enrolment, which has been a staple result in most of the studies across the globe.[41] These trends in sub and above normal BMI are indicative of the emerging dual burden of disease even the rural regions of central India.

Anemia and underweight co-existed among one-third of the study participants. Over the years of enrollment, there was an increase in the proportions of women who were both anemic and

For peer review only

underweight. This rise may be attributed to the rise prevalence of underweight alone as the rates of anemia did not change over the study period(Figure 2). As expected, BMI and hemoglobin concentrations were found to be significantly correlated. Underweight women were not only more likely to have anemia, but have higher rates of moderate and severe anemia. The most probable cause for maternal underweight in this region would be malnutrition.[42,43] Although this study did not assess the causes of anemia, it is likely that iron and other micronutrient deficiency would responsible for higher rates of anemia in underweight women.[44-46]

The rates of CS increased over the years consistent with an increase in facility based deliveries in India.[47] Anemia at any level reduced the likelihood of CS and pregnancy related complication. Underweight and anemic women are more likely to have fetal growth retardation and smaller babies.[48-51]The commonest cause of pregnancy related complication in this study population was obstructed labor and toxemia of pregnancy which is less likely to occur in underweight and anemic women who tend to have smaller babies.[47]Underweight and anemia increased the risk of LBW in this study population also. The risk of LBW in women with mild anemia increased by 8% and this risk tripled when women had severe/moderate anemia. A recent cohort of from the north eastern region of India also reported that severely anemic women had 6 and 16 fold odds of having an LBW baby or perinatal deaths respectively than normal/mildly anemic women [11].The risk of having LBW babies in women who were underweight also increased by 20%. This is consistent with other studies that have reported an association of maternal anthropometry and size of the baby.[2,3]A study in Tanzania, on 12,269 newborns, identified short maternal stature as an significant risk factor for SGA and preterm-appropriate for gestational age.[52]When a woman had both anemia and underweight, the risk of LBW was 1.5 times more

For peer review only

as compared to those who had normal BMI and were not anemic. A study investigating the effect of maternal malnutrition and anemia on the endocrine regulation of fetal growth found also significantly higher proportions of SGA neonates born to malnourished and anemic mothers than the mothers who were either malnourished or anemic.[53]The rates of LBW also increased over the years in women who had moderate/severe anemia and in women who were underweight (Table 2). Babies that are born LBW are likely to be stunted adults which will expound the inter-generational burden of LBW. [54]

Anemia also significantly increased the risk of stillbirths. It increased by 28% and 43% respectively in women who were mildly anemic and moderate/severely anemic as compared to non-anemic women. While there are studies to support our findings on the association of maternal anemia on stillbirths,[11,55,56] there is a need for understanding the affect of maternal underweight *per se* in the developing countries. Anemia could also reflect poor socio-economic conditions and limited access to quality intra-partum care which resulted in stillbirths. As previously mentioned, it could be speculated that other causes of anemia such as sickle cell anemia, hookworm infestation and malaria that are common in this region which may have contributed to higher rates of stillbirths. [20-25]Ethnicity has shown to influence on the risk of still births among underweight women previously.[57] We found that the increase in the risk was 10% in women who were underweight. Till date there is no published data on the augmented affect of underweight and anemia on stillbirths. We report when women were both anemia and underweight the rate of stillbirth was 1.5 times more than in women with normal weight and no anemia.

For peer review only

There is a paucity of data to understand association between anemia and neonatal mortality.[55, 58] We contribute to bridging this gap by analyzing 1638 infant deaths based on presence of pre-natal anemia among the mothers, or not. We found that the rates of neonatal deaths were nearly 1.4 times more in women with mild anemia as compared to who were non anemic and this risk further increased to 1.7 times for moderate/severe anemic women. This is consistent with the finding of a recent meta-analysis restricting to LMIC that found the odd of neonatal mortality to be 2.7 times among the anemic mothers.[59] However the analysis included just two studies, one from India and another from Malawi using the outcomes of 447 and 795 pregnant women, respectively.

We observed that the neonatal death outcome was worst in babies from underweight mothers compared to who were normal. Similar finding has been reported previously [60]A relatively recent meta-analysis shows a J shaped association between neonatal deaths and maternal BMI [61]. Though, non-significant, we also observed an increased risk of neonatal deaths (11%) among overweight/ obese women. Further, our study demonstrates the increase in risk of neonatal death to 1.7 folds when maternal anemia is compounded by underweight. Low maternal BMI and anemia are associated with low birth weight and preterm deliveries–the leading causes of infant mortality in India[62-63] and thus could have contributed to these deaths.

CONCLUSIONS AND PUBLIC HEALTH IMPLICATIONS

Over all, anemia and underweight in pregnancy increased the risk of stillbirths, neonatal deaths and delivering LBWs and this risk was further enhanced if the two conditions co-existed. These

For peer review only

finding raise major concerns as despite the national programs in place to address iron deficiency anemia, high rates of anemia among the rural pregnant women from central India failed to diminish over past 8 years. To worsen this, there has been a steady increase in underweight prevalence and almost a third of these women were both anemic and underweight. In view of above findings, immediate and effective public health intervention for addressing maternal anemia and malnutrition is needed to improve birth outcomes. There is an urgent need for evaluation and addressing of gaps of the iron supplementation program for adolescent girls and pregnant women in these areas with high rates of anemia, in addition to a more holistic approach to curb maternal malnutrition. Meanwhile, seeking simultaneously short term strategies such as referral to a better healthcare service and management of delivery for the segment of pregnant population who are both anemia and underweight could be helpful in reducing neonatal mortality. Such actions are imperative to break the intergenerational cycle of poor growth in the offspring and also for improving child survival.

LIST OF ABBREVIATIONS

ANM - Auxiliary Nurse Midwife

APH - Antepartum Hemorrhage

BMI - Body Mass Index

CI - Confidence Interval

CS - Cesarean Section

DLHS - District Level Household Survey

GEE - Generalized Estimating Equations

GN - Global Network

For peer review only

IFA - Iron Folic Acid

IRB - Institutional Review Board

LBW - Low Birth Weight

LMIC - Low and Middle Income Countries

LMRF - Lata Medical Research Foundation

MNHR - Maternal and Newborn Health Registry

MTP - Medical Termination of Pregnancy

NFHS - National Family and Health Survey

NICHD - *Eunice Kennedy Shriver* National Institute of Child Health and Human Development

PHC - Public health Center

PPH - Postpartum Hemorrhage

RA - Registry Administrator

RR - Relative Risk

SGA - Small for Gestational Age

USA - United States of America

WHO - World Health Organization

AUTHOR CONTRIBUTIONS

ABP conceived and designed the study, and developed the initial data collection tools; ABP and AAP wrote the first draft of the manuscript, which PLH, PKD, and SG subsequently revised. ABP and AAP developed the analysis plan outline and AAP carried out the data analyses. ABP,

For peer review only

PLH, PKD and SG contributed to data analysis through review, interpretation and cross-checking figures and tables. All authors read, revised, and approved the final manuscript.

ACKNOWLEDGEMENTS

We are deeply indebted to Research Triangle International, Durham, USA for their enthusiastic support. Thanks and recognition are due to the Registry Administrators at the Nagpur site of the MNHR who collect data, often in very challenging circumstances, and the communities, families, and mothers who agree to participate in the MNHR. We express our sincere gratitude to Dr. Yamini Pusdekar, Dr. Vaishali Khedikar, Dr. Savita Bhargav and Dr, Kunal Kurhe at LMRF, who helped immensely by strengthening the manuscript through their inputs.

DATA SHARING

A de-identified minimal dataset that underlies the findings and conclusions described in the manuscript can be shared upon request by the editors to verify the reported study findings. All authors hereby declare that individual participant data in any form is not a part of this publication.

For peer review only

FUNDING

This study was funded by the *Eunice Kennedy Shriver* National Institute of Child Health and Human Development of the US National Institutes of Health funded this study (Grants Reference Nos. U01HD058322 and U01HD078439).

ETHICS

The study in Nagpur, India has been reviewed and approved by the Institutional Review Board (IRB) of the Lata Medical Research Foundation (RPC#22A) and the Partners Human Research Committee/IRB in Boston, MA (H-35430).

CONSENT TO PARTICIPATE

The study was explained thoroughly and in detail, to the women who lived in the participating communities. The women were enrolled in the study only after giving informed consent. An IRB approved consent form was used for this purpose.

COMPETING INTERESTS

The authors declare no competing interests.

For peer review only

TRANSPARENCY

The lead author (ABP) affirms that the manuscript is an honest, accurate, and transparent account of the study being reported; that no important aspects of the study have been omitted; and that any discrepancies from the study as planned have been explained.

For peer review only

What is already known on this topic

Low body mass Index (BMI) and anemia during pregnancy have been independently identified as risk factors for delivering pre-term, small for gestational age (SGA) and LBWs, however their relationship with respect to neonatal mortality and stillbirth remains inconclusive. Also, their relationship of severity of anemia with adverse pregnancy outcomes is not well established for low middle income countries (LMIC). The risk arising out of co-existence of anemia and low BMI -a common scenario in LMIC like India, on poor maternal has not been characterized.

What this study adds

This study showed a high prevalence of anemia (>90%) and underweight (35%) among rural pregnant women in Maharashtra, Central India. The rate of anemia remained unaltered but there was an increase in the underweight and overweight/obese woman over the past 8 years from 2009-2016. This highlights the magnitude of persistent public health problem of nutrition origin despite the national programs in place and also the emerging dual burden of diseases in the rural areas as well.

Anemia and underweight during pregnancy were individually found to increase the risk of stillbirths, neonatal deaths and LBW. Women with moderate-severe anemia were at greater risk as compared to the mildly anemic women. These risks increased further if anemia and underweight were present together during pregnancy. This raises concerns as the two conditions often co-exist in LMICs and in the present study was, as high as, affecting a third of the study population.

Recommendations for policy

There is urgent need to address maternal malnutrition. Though holistic approach would be eradicating maternal malnutrition through more targeted and intensive food or medicinal supplement based approach, seeking short term strategies such as referral to a better healthcare service simultaneously could help in breaking the intergenerational cycle of poor growth in the offspring and reducing child mortality.

For peer review only

REFERENCES:

1. Black RE, Victora CG, Walker SP, et al. Maternal and child undernutrition and overweight in low-income and middle-income countries. *Lancet* 2013; 382(9890): 427-51. doi: 10.1016/S0140-6736(13)60937-X. (accessed 30 November, 2017).
2. Bhutta ZA, Das JK, Rizvi A, et al. Evidence-based interventions for improvement of maternal and child nutrition: what can be done and at what cost? *Lancet* 2013; 382(9890): 452-77. doi:10.1016/S0140-6736(13)60996-4.(accessed 30 November, 2017).
3. Black RE, Allen LH, Bhutta ZA, et al. Maternal and child undernutrition: global and regional exposures and health consequences. *Lancet* 2008; 371(9608):243-60. doi: 10.1016/S0140-6736(07)61690-0.(accessed30 November, 2017).
4. United Nations Development Program. *Sustainable Development Goals*. http://www.undp.org/content/undp/en/home/sustainable-development-goals.html (accessed 30 November 2017).
5. World Health Organization. *Global nutrition targets 2025: policy brief series (WHO/NMH/NHD/14.2).2014*.http://apps.who.int/iris/bitstream/10665/149018/1/WHO_NMH_NHD_14.2_eng.pdf?ua=1(accessed on 11 Sept 2017).
6. Government of India. *Millennium Development Goals: India Country Report 2015*. http://mospi.nic.in/sites/default/files/publication_reports/mdg_2july15_1.pdf(accessed on 11 Sept 2017).
7. United Nations Inter-agency Group for Child Mortality. *Levels & Trends in Child Mortality: Report 2015*. https://www.unicef.org/publications/files/Child_Mortality_Report_2015_Web_9_Sept_15.pdf(accessed on 30 Nov 2017).
8. United Nations Children's Fund. *Committing to Child Survival: A promise renewed. Progress Report 2015*. http://www.apromiserenewed.org/wp-content/uploads/2015/09/APR_2015_8_Sep_15.pdf(accessed on 30 Nov 2017).
9. Van Der Linden EL, Browne JL, Vissers KM, et al. Maternal body mass index and adverse pregnancy outcomes: A Ghanaian cohort study. *Obesity* 2016; 24(1): 215-22. doi:10.1002/oby.21210. (accessed on 30 Nov 2017).

For peer review only

10. International Institute of Population Sciences. *National Family Health Survey-4: Maharashtra State Fact Sheet*. http://rchiips.org/nfhs/pdf/NFHS4/MH_FactSheet.pdf (accessed 30 November 2017).
11. Nair M, Choudhury MK, Choudhury SS, et al. Association between maternal anemia and pregnancy outcomes: a cohort study in Assam, India. *BMJ Glob Health* 2016;1:e000026. doi:10.1136/bmjgh-2015-000026.(accessed30th November 2017).
12. Haider BA, Olofin I, Wang M, et al. Anemia, prenatal iron use, and risk of adverse pregnancy outcomes: systematic review and meta-analysis.*BMJ* 2013;346:f3443. doi:10.1136/bmj.f3443. (accessed30 November 2017).
13. Goudar SS, Carlo WA, McClure EM, et al. The maternal and newborn health registry study of the global network for women's and children's health research. *Int J Gynaecol Obstet* 2012; 118(3): 190-3. doi:10.1016/j.ijgo.2012.04.022
14. World Health Organization. *Global database on body mass index.* Http://apps.who.int/bmi/index.jsp?introPage=intro_3.html (accessed 30 November 2017)
15. U.S.Agency for International Development/ PATH. *A Manual for Health Workers: Anemia Detection Methods in Low-Resource Settings*;1997. http://pdf.usaid.gov/pdf_docs/PNACC882.pdf(accessed30 November 2017).
16. World Health Organization (WHO*). Preventing and controlling anemia through primary health care: A guide for health administrators and program managers*. Geneva: WHO; 1989. http://www.who.int/nutrition/publications/micronutrients/anaemia_iron_deficiency/9241542497.pdf (accessed 30 November 2017).
17. Cuzick J. A Wilcoxon-type test for trend. *Stat Med* 1985;4(1):87–90.
18. International Institute for Population Sciences (IIPS) and Macro International. *National Family Health Survey (NFHS-3), 2005–06, India, Volume I*. Mumbai: IIPS;2007. http://rchiips.org/nfhs/NFHS-3%20Data/VOL-1/India_volume_I_corrected_17oct08.pdf(accessed 1 December 2017).
19. International Institute of Population Sciences(IIPS). *District Level Household and Facility Survey -4. State Fact Sheet Maharashtra (2012-13)*. http://rchiips.org/pdf/dlhs4/report/MH.pdf (accessed 22 November 2017).

For peer review only

20. World Health Organization. *The prevalence of anemia in women: a tabulation of available information (WHO/MCH/MSM/92)*. 2nd ed. Geneva: World Health Organization; 1992. http://apps.who.int/iris/bitstream/10665/58994/1/WHO_MCH_MSM_92.2.pd (accessed 30 November 2017)

21. Bharati S, Pal M, Som S, Bharati P. Temporal trend of anemia among reproductive-aged women in India. *Asia Pac J Public Health*2015;27(2):NP1193-1207.

22. Balarajan YS, Fawzi WW, Subramanian SV. Changing patterns of social inequalities in anemia among women in India: cross-sectional study using nationally representative data. *BMJ Open* 2013;3(3):e002233 (accessed 1 December 2017).

23. Kalaivani K. Prevalence & consequences of anemia in pregnancy. *Indian J Med Res* 2009;130(5):627–33.

24. Colah RB, Mukherjee MB, Martin S,et al. Sickle cell disease in tribal populations in India. *Indian J Med Res*2015;141(5):509-515. doi:10.4103/0971-5916.159492 (accessed 1 December 2017).

25. Corrêa G, Das M, Kovelamudi R,et al. High burden of malaria and anemia among tribal pregnant women in a chronic conflict corridor in India. *Confl and Health 2017*; 11: 10. doi: 10.1186/s13031-017-0113-1 (accessed 1 December 2017).

26. Ahankari AS, Myles PR, Dixit JV,et al. Risk factors for maternal anemia and low birth weight in pregnant women living in rural India: a prospective cohort study. *Public Health* 2017 Oct;151:63-73. doi: 10.1016/j.puhe.2017.06.023(accessed 1 December 2017)

27. Arlappa N, Meshram II, Balakrishna N,et al. Prevalence of anemia among different physiological groups in the rural areas of Maharashtra. *Indian J Community Health* 2014; 26(3):278-284.

28. Ahankari A, Bapat S, Myles P, et al. Factors associated with preterm delivery and low birth weight: a study from rural Maharashtra, India. *F1000Res*2017;6:72. doi: 10.12688/f1000research.10659.1 1 (accessed 1 December 2017).

29. Wadgave HV. Burden of anemia among the pregnant women in rural Area. Healthline 2011;2:76-7.http://www.iapsmgc.org/index_pdf/53.pdf (accessed 1 December 2017).

30. Balasubramaniam P, Malathi A. Comparative study of hemoglobin estimated by Drabkin's and Sahli's methods.*J Postgrad Med* 1992; 38(1):8-9.

For peer review only - http://bmjopen.bmj.com/site/about/guidelines.xhtml

31. Rakesh PS. Prevalence of anemia in Kerala State, Southern India - a systematic review.*J ClinDiagn Res* 2017;11(5):LE01-LE04. doi: 10.7860/JCDR/2017/24681.9951(accessed 1 December 2017).
32. Pasricha SR, Biggs BA, Prashanth NS, et al. Factors influencing receipt of iron supplementation by young children and their mothers in rural India: local and national cross-sectional studies. *BMC Public Health*2011;11:617. doi: 10.1186/1471-2458-11-617(accessed 1 December 2017).
33. Chaturvedi S, Ranadive B.Are we really making motherhood safe? A study of provision of iron supplements and emergency obstetric care in rural Maharashtra.*Natl Med J India* 2007; 20(6):294-6.
34. Kumar V,Singh P. Insurance coverage under different health schemes in Uttar Pradesh, India. *ClinEpidemiol Glob Health*2017; 5(1): 1-7.http://www.ceghonline.com/article/S2213-3984(16)30011-2/fulltext (accessed 1 December 2017).
35. UNICEF. *National Factsheet, Coverage Evaluation Survey, 2009*. New Delhi: UNICEF India Country Office; 2010. http://www.indiawaterportal.org/sites/indiawaterportal.org/files/National%20Factsheet_Coverage%20Evaluation%20Survey_UNICEF_2009.pdf (accessed 25 November 2017).
36. Pal PP, Sharma S, Sarkar TK,et al. Iron and folic acid consumption by the ante-natal mothers in a rural area of India in 2010. *Int JPrev Med* 2013;4(10):1213-1216.
37. Mithra P, Unnikrishnan B, Rekha T, et al. Compliance with iron-folic acid (IFA) therapy among pregnant women in an urban area of south India. *Afr Health Sci* 2013;13(4):880-885. 10.4314/ahs.v13i4.3(accessed 1 December 2017).
38. Galloway R, Dusch E, Elder L,et al. Women's perceptions of iron deficiency and anemia prevention and control in eight developing countries. *SocSci Med* 2002;55(4):529-44.
39. Kotecha PV, Nirupam S, Karkar PD. Adolescent girls' anemia control program, Gujarat, India. Indian J Med Res 2009;130(5):584-9.
40. Vir SC, Singh N, Nigam AK, et al. Weekly iron and folic acid supplementation with counseling reduces anemia in adolescent girls: A large-scale effectiveness study in Uttar Pradesh, India. *Food Nutr Bull*2008;29:186-94.

41. NCD Risk Factor Collaboration (NCD-RisC).Trends in adult body-mass index in 200 countries from 1975 to 2014: a pooled analysis of 1698 population-based measurement studies with 19.2 million participants. Lancet 2016;387(10026):1377-1396. doi: 10.1016/S0140-6736(16)30054-X(accessed 1 December 2017).

42. National Nutrition Monitoring Bureau (NNMB). Diet and nutritional status of rural population, prevalence of hypertension and diabetes among adults and infant and young child feeding practices-A report of third repeat survey. Technical report no. 26.Hyderabad: National Institute of Nutrition, Indian Council of Medical Research; 2012. http://nnmbindia.org/1_NNMB_Third_Repeat_Rural_Survey___Technicl_Report_26.pdf (accessed 1 December 2017).

43. Mahanta LB, Choudhury M, Devi A, et al. On the Study of Pre-Pregnancy Body Mass Index (BMI) and Weight Gain as Indicators of Nutritional Status of Pregnant Women Belonging to Low Socio-Economic Category: A Study from Assam. *Indian J Community Med* 2015;40(3):198-202. doi: 10.4103/0970-0218.158870 (accessed 1 December 2017).

44. Sumarmi S, Puspitasari N, Handajani R, et al. Underweight as a risk factor for iron depletion and iron-deficient erythropoiesis among young women in rural areas of East Java, Indonesia.*Malays J Nutr* 2016;22(2):219–32.

45. Hettiarachchi M, Liyanage C, Wickremasinghe R, et al.Prevalence and severity of micronutrient deficiency: a cross-sectional study among adolescents in Sri Lanka.*Asia Pac J ClinNutr* 2006;15(1):56-63.

46. Darnton-Hill I, Mkparu UC. Micronutrients in Pregnancy in Low- and Middle-Income Countries. *Nutrients* 2015;7(3):1744-1768. doi:10.3390/nu7031744 (accessed on 1 December 2017). https://link.springer.com/article/10.1186/s13690-017-0181-0

47. Patel AB, Prakash AA, Raynes-Greenow C, et al. Description of inter-institutional referrals after admission for labor and delivery: a prospective population based cohort study in rural Maharashtra, India.BMC Health Serv Res2017; 17(1):360. doi: 10.1186/s12913-017-2302-4 (accessed 1 December, 2017).

48. Sebastian T, Yadav B, Jeyaseelan L, et al. Small for gestational age births among South Indian women: temporal trend and risk factors from 1996 to 2010. *BMC Pregnancy and Childbirth*2015;15:7. doi:10.1186/s12884-015-0440-4 (accessed 1 December 2017).

49. Kaur M, Chauhan A, Manzar MD, et al. Maternal anemia and neonatal outcome: a prospective study on urban pregnant women. *J ClinDiagn Res* 2015;9(12):QC04-QC08. doi:10.7860/JCDR/2015/14924.6985 (accessed 1 December 2017).

50. Verma A, Shrimali L. Maternal body mass index and pregnancy outcome. J ClinDiagn Res 2012;6(9):1531-1533. doi:10.7860/JCDR/2012/4508.2551(accessed 1 December 2017).

51. Haider BA, Olofin I, Wang M, et al. Anemia, prenatal iron use, and risk of adverse pregnancy outcomes: systematic review and meta-analysis. *The BMJ*2013;346:f3443. doi:10.1136/bmj.f3443(accessed 1 December 2017).

52. Muhihi A, Sudfeld CR, Smith ER, et al. Risk factors for small-for-gestational-age and preterm births among 19,269 Tanzanian newborns. *BMC Pregnancy Childbirth* 2016;16:110. doi:10.1186/s12884-016-0900-5(accessed 1 December 2017).

53. Mahajan SD, Singh S, Shah P, et al. Effect of maternal malnutrition and anemia on the endocrine regulation of fetal growth. *Endocr Res* 2004; 30(2):189-203. Varela-Silva MI, Azcorra H, Dickinson F, Bogin B, Frisancho AR. Influence of maternal stature, pregnancy age, and infant birth weight on growth during childhood in Yucatan, Mexico: a test of the intergenerational effects hypothesis. *Am J Hum Biol* 2009; 21(5):657-63. doi: 10.1002/ajhb.20883 (accessed 6 December 2017)

54. Varela-Silva MI, Azcorra H, Dickinson F, et al. Influence of maternal stature, pregnancy age, and infant birth weight on growth during childhood in Yucatan, Mexico: a test of the intergenerational effects hypothesis. *Am J Hum Biol* 2009; 21(5):657-63. doi: 10.1002/ajhb.20883 (accessed 6 December 2017)

55. Tandu-Umba B, Mbangama AM. Association of maternal anemia with other risk factors in occurrence of Great obstetrical syndromes at university clinics, Kinshasa, DR Congo. *BMC Pregnancy Childbirth* 2015; *15:* 183. doi: 10.1186/s12884-015-0623-z(accessed 1 December 2017).

56. Ali AA, Adam I.Anaemia and stillbirth in Kassala Hospital, Eastern Sudan.*J Trop Pediatr* 2011;57(1):62-4.

57. Salihu HM, Mbah AK, Alio AP, et al. Maternal prepregnancy underweight and risk of early and late stillbirth in black and white gravidas.*J Natl Med Assoc*2009;101(6):582-7

58. Ramakrishnan U, Grant F, Goldenberg T, et al. Effect of women's nutrition before and during early pregnancy on maternal and infant outcomes: a systematic review.PaediatrPerinatEpidemiol, 26: 285–301. doi:10.1111/j.1365-3016.2012.01281.x (accessed 1 December 2017).

59. Rahman MM, Abe SK, Rahman MS, et al. Maternal anemia and risk of adverse birth and health outcomes maternal in low- and middle-income countries: systematic review and meta-analysis.*Am J ClinNutr*2016;103(2):495-504.

60. Kalk P, Guthmann F, Krause K, et al.Impact of maternal body mass index on neonatal outcome.*Eur J Med Res* 2009 May 14; 14(5): 216-22.

61. Aune D, Saugstad OD, Henriksen T, Tonstad S.Maternal body mass index and the risk of fetal death, stillbirth, and infant death: a systematic review and meta-analysis.*JAMA*2014;311(15):1536-46.

62. Million Death Study Collaborators,Bassani DG, Kumar R, et al. Causes of neonatal and child mortality in India: nationally representative mortality survey. *Lancet*2010;376(9755):1853-60. doi: 10.1016/S0140-6736(10)61461-4 (accessed 1 December 2017).

63. Sankar MJ, Neogi SB, Sharma J, et al. State of newborn health in India. *J Perinato* 2016; 36(3), S3–S8. doi: 10.1038/jp.2016.183 (accessed 1 December 2017).

only

Figure 1: Categorical anemia (gm/dl)in included women by body mass index (kg/m^2), over year of enrolment

Figure1: Pic

For peer review only

Figure 2: Proportions of Anemia (gm/dl) & body mass index (kg/m^2) interaction in included women, over year of enrollment

Figure2: Pic

For peer review only

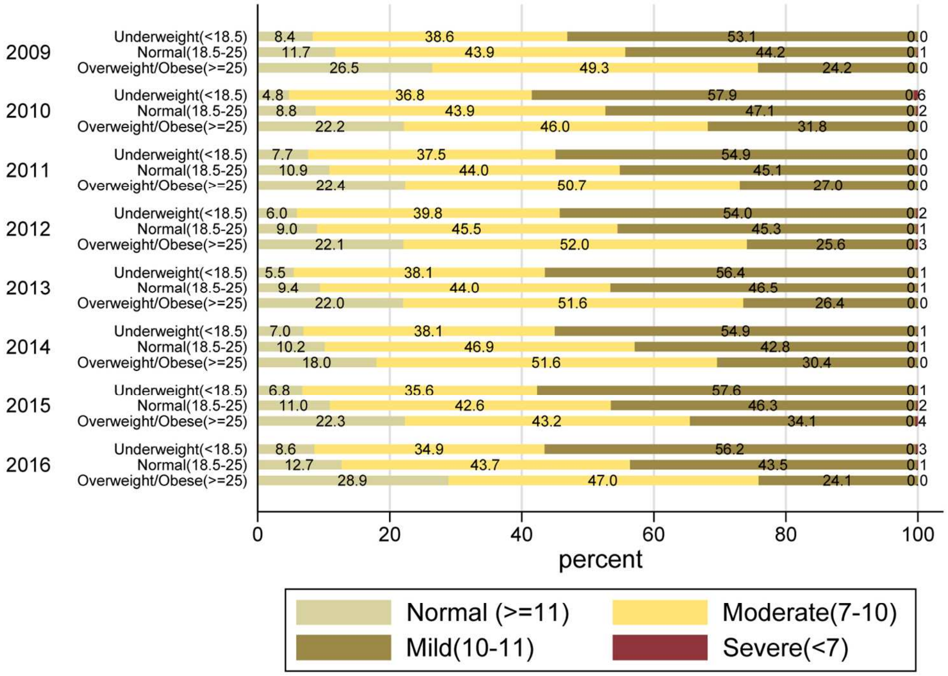

Figure 1: Categorical anemia (gm/dl)in included women by body mass index (kg/m2), over year of enrolment

139x101mm (300 x 300 DPI)

For peer review only - http://bmjopen.bmj.com/site/about/guidelines.xhtml

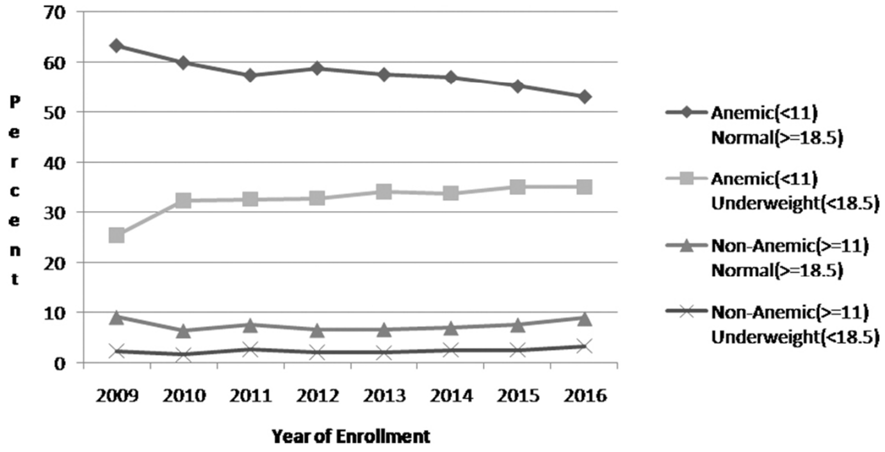

Figure 2: Proportions of Anemia (gm/dl) & body mass index (kg/m2) interaction in included women, over year of enrollment

101x49mm (300 x 300 DPI)

For peer review only

STROBE Statement—Checklist of items that should be included in reports of ***cross-sectional studies***

	Item No	Recommendation
**Title and abstract**	1	(*a*) Indicate the study's design with a commonly used term in the title or the abstract ☑ - ***Page 1***
		(*b*) Provide in the abstract an informative and balanced summary of what was done and what was found ☑ - ***Page 4***
**Introduction**		
Background/rationale	2	Explain the scientific background and rationale for the investigation being reported ☑ - ***Page 8***
Objectives	3	State specific objectives, including any prespecified hypotheses ☑ - ***Page 9***
**Methods**		
Study design	4	Present key elements of study design early in the paper ☑ - ***Page 10***
Setting	5	Describe the setting, locations, and relevant dates, including periods of recruitment, exposure, follow-up, and data collection ☑ - ***Page 10***
Participants	6	(*a*) Give the eligibility criteria, and the sources and methods of selection of participants ☑ - ***Page 10***
Variables	7	Clearly define all outcomes, exposures, predictors, potential confounders, and effect modifiers. Give diagnostic criteria, if applicable ☑ - ***Page 11 & 12***
Data sources/ measurement	8*	For each variable of interest, give sources of data and details of methods of assessment (measurement). Describe comparability of assessment methods if there is more than one group ☑ - ***Page 11 & 12***
Bias	9	Describe any efforts to address potential sources of bias ☑ - ***Page 12 & 13***
Study size	10	Explain how the study size was arrived at - N/A
Quantitative variables	11	Explain how quantitative variables were handled in the analyses. If applicable, describe which groupings were chosen and why ☑ - ***Page 11, 12 & 13***
Statistical methods	12	(*a*) Describe all statistical methods, including those used to control for confounding ☑ - ***Page 13***
		(*b*) Describe any methods used to examine subgroups and interactions ☑ - ***Page 13***
		(*c*) Explain how missing data were addressed ☑ - ***Page 13 & 14***
		(*d*) If applicable, describe analytical methods taking account of sampling strategy ☑ - ***Page 13***
		(*e*) Describe any sensitivity analyses - N/A
**Results**		
Participants	13*	(a) Report numbers of individuals at each stage of study—eg numbers potentially eligible, examined for eligibility, confirmed eligible, included in the study, completing follow-up, and analysed ☑ - ***Page 14***
		(b) Give reasons for non-participation at each stage - N/A
		(c) Consider use of a flow diagram - N/A
Descriptive data	14*	(a) Give characteristics of study participants (eg demographic, clinical, social) and information on exposures and potential confounders ☑ - ***Page 15***
		(b) Indicate number of participants with missing data for each variable of interest ☑ - ***Page 14***
Outcome data	15*	Report numbers of outcome events or summary measures ☑ - ***Page 17***
Main results	16	(*a*) Give unadjusted estimates and, if applicable, confounder-adjusted estimates and their precision (eg, 95% confidence interval). Make clear which confounders were adjusted for and why they were included ☑ - ***Page 19 thru 21***

For peer review only

		(*b*) Report category boundaries when continuous variables were categorized ☑ - ***Page 11 & 12***
		(*c*) If relevant, consider translating estimates of relative risk into absolute risk for a meaningful time period - N/A
Other analyses	17	Report other analyses done—eg analyses of subgroups and interactions, and sensitivity analyses ☑ - ***Page 19 & 21***
**Discussion**		
Key results	18	Summarise key results with reference to study objectives ☑ - ***Page 17 thru 21***
Limitations	19	Discuss limitations of the study, taking into account sources of potential bias or imprecision. Discuss both direction and magnitude of any potential bias ☑ - ***Page 6***
Interpretation	20	Give a cautious overall interpretation of results considering objectives, limitations, multiplicity of analyses, results from similar studies, and other relevant evidence ☑ - ***Page 22 thru 26***
Generalisability	21	Discuss the generalisability (external validity) of the study results ☑ - ***Page 22 thru 27***
**Other information**		
Funding	22	Give the source of funding and the role of the funders for the present study and, if applicable, for the original study on which the present article is based ☑ - ***Page 30***

*Give information separately for exposed and unexposed groups.

Note: An Explanation and Elaboration article discusses each checklist item and gives methodological background and published examples of transparent reporting. The STROBE checklist is best used in conjunction with this article (freely available on the Web sites of PLoS Medicine at http://www.plosmedicine.org/, Annals of Internal Medicine at http://www.annals.org/, and Epidemiology at http://www.epidem.com/). Information on the STROBE Initiative is available at www.strobe-statement.org.

For peer review only

BMJ Open

MATERNAL ANEMIA AND UNDERWEIGHT AS DETERMINANTS OF PREGNANCY OUTCOMES: COHORT STUDY IN EASTERN RURAL MAHARASHTRA, INDIA

Journal:	*BMJ Open*
Manuscript ID	bmjopen-2018-021623.R1
Article Type:	Research
Date Submitted by the Author:	08-Mar-2018

Complete List of Authors:	Patel, Archana; Lata Medical Research Foundation, Nagpur<br>Prakash, Amber; Lata Medical Research Foundation, Nagpur, Research Unit<br>Das, Prabir Kumar; Lata Medical Research Foundation, Nagpur<br>Gupta, Swarnim ; Lata Medical Research Foundation, Nagpur<br>Pusdekar, Yamini; Lata Medical Research Foundation, Nagpur<br>Hibberd, Patricia; Boston University School of Public Health; Boston University School of Medicine

**Primary Subject Heading**:	Public health
Secondary Subject Heading:	Global health, Epidemiology, Nutrition and metabolism
Keywords:	Body Mass Index, Anemia, Pregnancy, Neonatal Deaths, Malnutrition, Stillbirth

SCHOLARONE™
Manuscripts

only

MATERNAL ANEMIA AND UNDERWEIGHT AS DETERMINANTS OF PREGNANCY OUTCOMES: COHORT STUDY IN EASTERN RURAL MAHARASHTRA, INDIA

Archana Patel[1], Amber Abhijeet Prakash*[1], Prabir Kumar Das[1], Swarnim Gupta[1], Yamini Pusdekar[1], Patricia L Hibberd[2]

1. Lata Medical Research Foundation, Nagpur, India

2. Boston University School of Public Health and Boston University School of Medicine, Boston, United Stated of America

First Author:

Archana Patel (MD PhD)

Program Director

Address: Lata Medical Research Foundation,

Vasant Nagar, Nagpur- 440022,

Maharashtra, India

Telephone: +0712-2249569

Email: dr_apatel@yahoo.com

*Second and Corresponding Author:

Amber Abhijeet Prakash (MSc)

Senior Statistician and Data Manager

Address: Lata Medical Research Foundation,

Vasant Nagar, Nagpur- 440022,

For peer review only

Maharashtra, India
Telephone: +0712-2249569
Email: rockman.blues21@gmail.com

Third Author:
Prabir Kumar Das (MD)
Country Coordinator
Address: Lata Medical Research Foundation,
Vasant Nagar, Nagpur- 440022,
Maharashtra, India
Telephone: +0712-2249569
Email: prabir_das23@rediffmail.com

Fourth Author:
Swarnim Gupta (PhD)
Nutrition Consultant
Address: Lata Medical Research Foundation,
Vasant Nagar, Nagpur- 440022,
Maharashtra, India
Telephone: +0712-2249569
Email: gupta.swarnim@gmail.com

Fifth Author:

For peer review only

Yamini Pusdekar (MD)

Research Co-ordinator

Address: Lata Medical Research Foundation,

Vasant Nagar, Nagpur- 440022,

Maharashtra, India

Telephone: +0712-2249569

Email: dryaminipusdekar27@gmail.com

Sixth Author:

Patricia L Hibberd (MD PhD)

Chair, Department of Global Health

Professor of Global Health, Boston University School of Public Health

Professor of Medicine, Boston University School of Medicine

372 Crosstown Building

801 Massachusetts Avenue

Boston, MA 02118

Email:plh0@bu.edu

Telephone:617-414-1260

WORD COUNT: 5,608

For peer review only

COPYRIGHT

The Corresponding Author has the right to grant on behalf of all authors and does grant on behalf of all authors, an exclusive license (or non exclusive for government employees) on a worldwide basis to the BMJ Publishing Group Ltd to permit this article (if accepted) to be published in BMJ editions and any other BMJPGL products and sublicenses such use and exploit all subsidiary rights, as set out in our license

For peer review only

ABSTRACT

Objectives: To study the trend in the prevalence of anemia and low BMI pregnant women from Eastern Maharashtra and evaluate if low BMI and anemia affects pregnancy outcomes.

Design: Prospective observational cohort study.

Setting: Catchment areas of 20 rural primary health centers in 4 eastern districts of Maharashtra State, India.

Participants: 72,750 women from the Nagpur site of Maternal and Newborn Health Registry of NIH's Global Network, enrolled from 2009 to 2016.

Main outcome measures: Mode of delivery, pregnancy related complications at delivery, stillbirths, neonatal deaths and low birth weight (LBW) in babies.

Results: Over 90% of the women included in the study were anemic and about a third were underweight (BMI<18 kg/m^2). Any degree of anemia significantly increased the risk of stillbirth, neonatal deaths and LBW babies ($p<0.05$). Risks were higher among the moderate to severely anemic women vs. those with mild anemia for post natal outcomes Risk ratio; 95% confidence interval (RR;(95% CI) for stillbirth (1.4;(1.2 - 1.8) vs. 1.3;(1.1 - 1.6)); for neonatal deaths (1.7;(1.3 - 2.1)vs.1.3;(1.0 - 1.6)); for LBW (<2500gms)(1.3;(1.2 - 1.4)vs. 1.1;(1.0 - 1.2)). Low maternal BMI increased the neonatal deaths (1.1;(1.0 - 1.3)), andLBW babies (1.2;(1.2 - 1.3)).

The risk of having stillbirths (1.5;(1.2 - 1.8)), neonatal deaths (1.7;(1.3 - 2.3)) and LBW babies (1.5;(1.4 - 1.6)) was highest when the anemia and underweight co-existed in the included women. Obesity/overweight during pregnancy increased the risk of maternal complications at delivery (1.6;(1.5 - 1.7)) and of cesarean section (1.5;(1.4 - 1.6)).

Conclusions: Maternal anemia and low BMI in pregnancy were individually associated with enhanced risk of stillbirth, neonatal deaths and LBW. This risk increased further if anemia and low BMI were present simultaneously.

Trial registration: Registered at ClinicTrials.gov (NCT01073475)

Keywords: Body Mass Index, anemia, pregnancy, neonatal deaths, malnutrition, stillbirth, low birth weight

For peer review only

STRENGTHS AND LIMITATIONS

1. This is a large prospective study with a sample size of 72,750 pregnant women collected over an 8 year period in the target geographical region of Eastern Maharashtra, focusing on the synergistic effects of low body mass index (BMI) and anemia in pregnant women which may have been overlooked in prior research.
2. Dietary practices and details of compliance with iron supplementation data was not collected in this study.
3. Pre-pregnancy data on BMI and anemia were not available in this study.
4. The hemoglobin levels were measured using Sahli's method, which may not be accurate due to subjectivity of the method, poor sensitivity and lack of reliability.

For peer review only

INTRODUCTION

Malnutrition is a serious underlying cause for child and maternal deaths around the globe. Undernutrition during pregnancy restricts fetal growth, contributing to about 800,000 neonatal and 400,000 infant deaths, and 20% of stunting in the first 2 years of the child's life, as well as 20% of maternal deaths at delivery. [1-3]The The Sustainable Development Goals (SDG) bring explicit attention to nutrition, including the World Health Assembly target to reduce anemia in women of ages 15-49 years by 50% by 2025.[4-5] Since India has largest number of neonatal, infant and under age 5 children deaths in the world as well as high rates of stunting and growth faltering,[6-8], it is particularly timely to understand maternal nutritional status as a risk factor for maternal and childhood adverse outcomes of pregnancy in the Indian population.

Two important indicators of maternal nutrition are Body Mass Index (BMI) and anemia, both of which can affect health of a mother and her fetus. Much attention has been focused on complications of maternal overweight and obesity (BMI>25 kg/m^2) include gestational hypertension, pre-eclampsia, macrosomia, early induction of labor and need for caesarian deliveries and currently apply mostly to high but increasingly middle income countries, including India.[9]However, in rural India, undernutrition (BMI<18.5 kg/m^2) predominates and is associated with low birth weight (LBW, <2.5kg) and preterm deliveries (<37 weeks of gestation). Maternal anemia, as a result of undernutrition and infections coexist with low BMI, particularly in Indian women of reproductive age. In the 2015 National Family and Health Survey (NFHS), 23% of Indian women had a BMI less than 18.5 kg/m^2 and 53% were anemic (Hemoglobin < 11 gm/dL). Even higher rates were reported in rural areas of the state of

For peer review only

Maharashtra.[10] Maternal anemia is also associated with post-partum hemorrhage, LBW, small for gestational age (SGA) babies and perinatal death.[11,12]. However, there is minimal information on the consequences of the combination of low BMI and anemia in pregnant women in rural Maharashtra and whether women with this dual burden should be targeted for specific interventions. We used data from an ongoing Maternal and Newborn Health Registry (MNHR) based in rural areas of Nagpur, Maharashtra, India to (1) describe the prevalence and trends of maternal anemia and low BMI from 2009 to 2016 (2) describe the maternal demographic characteristics associated with different levels of maternal hemoglobin and BMI from 2009-2016, (3) evaluate whether low BMI and anemia were independent or synergistic risk factors for adverse pregnancy outcomes between 2009 and 2016, (4) investigate whether low BMI and anemia had additive or synergistic effects on poor birth outcomes.

METHODS

Study design and settings

The present study utilizes data collected from the Nagpur, India site of MNHR, which is a population based registry established in 2009 by the Global Network (GN) with support from *Eunice Kennedy Shriver* National Institute of Child Health and Human Development (NICHD), United States of America (USA). The MNHR is an observational, prospective cohort operating in seven different sites around the world including two sites, in India. The Nagpur site comprises of 20 study clusters in the 4 districts of Nagpur, Bhandara, Chandrapur and Wardha in Eastern Maharashtra, India. Geographical areas surrounding government primary health centers (PHC) that have approximately 900 deliveries per year, constitute a study cluster. The MNHR aims to

For peer review only

recruit all consenting women residing within the clusters, as early as possible during their pregnancy and then to follow them until 42 days post-partum. The MNHR methods have been published elsewhere. [13] The time period of this study was from June 2009 to December 2016.

Patient and Public Involvement

The research questions for this study, were developed to evaluate frequent reports of tiredness and weakness by the pregnant women enrolled in the prospective MNHR at the Nagpur site in rural Eastern Maharashtra and to further review the reported high prevalence of maternal anemia in the Indian Demographic Health Surveys. Specifically, we wanted to determine whether rates of anemia had decreased over time, as a result of an active national iron supplementation program, and, whether in our prospective cohort, these rates had an impact on pregnancy outcomes. We also wanted to study whether low BMI, common in our study subjects, further exacerbated the impact of anemia in pregnancy on perinatal outcomes. Since we intended to address these questions in a secondary data analysis using de-identified data in the MNHR, patients and public were not involved in the design of this study. Women enrolled in the MNHR were recruited through community awareness programs and by training and engaging community health care providers. The results will not be disseminated to study participants but are intended for additional research to find ways to reduce maternal undernutrition (low BMI) and anemia, as well as to inform health policies on pre-pregnancy and maternal nutrition.

Participants

For peer review only

The participants in this study, were all consenting pregnant women residing in the 20 clusters of the Nagpur Global Network site, enrolled within the study period,, who completed data collection (i.e., had delivery outcomes and a completed 42 days post-partum follow-up) at the time of analyses. Women who had medical termination of pregnancy (MTP) (premature expulsion of a non-viable fetus before 20 weeks of gestation), miscarriages, multiple fetuses or had extreme/missing values for one or more study variables of interest were excluded from the analyses. Early enrollment of the participants was ensured through active surveillance in the study clusters by Nagpur site MNHR public health staff. The enrolled women were followed through to a perinatal visit after delivery (within a week) and to a follow up visit around 42 days after birth. Maternal and infant data on study variables and outcomes, were recorded for the enrollees.

Data collection procedures

Briefly, data were collected by trained registry administrators (RA) via standardized forms. RAs included medical officers and auxiliary nurse-midwives (ANM) employed at PHCs and sub-centers. A two day training for data collection was provided to the Registry Administrators (RAs), followed by a refresher training every six months, and unscheduled special trainings as needed. The RAs returned filled in forms which were manually scrutinized for errors prior to data entry at Lata Medical Research Foundation (LMRF), Nagpur. After entry, data were transmitted to the Global Network (GN) data management center (Research Triangle Institute, Durham, North Carolina, USA) where digital data checks were performed on a monthly basis. The data management center forwarded edit, monitoring and performance reports to the Nagpur

For peer review only

site team for data cleaning and management. The data were continually reviewed for quality by NICHD appointed data monitoring committee and by the Nagpur site data collection team.

Study variables

At the first antenatal visit, information regarding the enrolled women's, characteristics including age, education, parity, height, weight and hemoglobin was recorded. Weight to nearest 100 grams using a spring balance and height to nearest centimeter was measured using a non-flexible measuring tape fixed to a wall. Body mass index (BMI) was calculated by dividing weight in kilograms (kg) by squared height in meters. Standard World Health Organization (WHO) BMI classifications [14] were used to categorize the enrollees as underweight (BMI<18.5 Kg/m^2), normal (BMI 18.5-24.9 Kg/m^2), or as overweight/ obese (BMI≥25Kg/m^2). Hemoglobin was estimated within two weeks of enrollment using Sahli's method. [15]A concentration of hemoglobin (Hb) level < 11 g/dL indicated presence of anemia. Based on WHO classification [16] hemoglobin readings were used to define the severity of anemia as normal (≥11g/dL), mild (10<11 g/dL) and moderate/severe (<10 g/dL). A 4 level combination variable for BMI-anemia was defined as based on anemia - hemoglobin<11(g/dL) (anemic) vs. ≥ 11 (g/dL) (not anemic) and BMI<18.5 (Kg/m^2) (underweight) vs ≥ 18.5 (Kg/m^2) (not underweight), creating 'anemic-normal', 'anemic-underweight', 'non anemic-normal' and 'non anemic-underweight'. Mother's age was recorded at the time of enrolment. Maternal education of up to 4 grades was considered 'primary', grade 5 to grade 10 as 'secondary' and more than grade 10 as 'university'. Parity was recorded as whole numbers zero or more, depending on if the woman had been pregnant before enrollment or in pregnancy had carried a fetus to 20 weeks of gestation, or not.

For peer review only

Study Outcomes

The impact of 2 level variables of anemia (yes/no) and BMI (underweight/normal) was assessed on rates of mode of delivery and any pregnancy related complications at delivery- specifically stillbirths, neonatal deaths and low birth weight (LBW). While we did not assess the determinants of maternal mortality as maternal mortality rates were low, we described the causes of death with respect to the level of anemia and BMI status.. Mode of delivery was recorded as cesarean section (CS) or non-cesarean (vaginal, assisted vaginal). Any pregnancy related complications at delivery were recorded as presence of one or more of the following; obstructed/prolonged labor, severe antepartum hemorrhage (APH), severe postpartum hemorrhage (PPH), hypertension, pre-eclampsia, and oblique/abnormal lie. Birth outcomes such as stillbirth, neonatal deaths (deaths of neonates from birth to 28 days of life) and LBW rates were assessed. Stillbirth rates were calculated as number of stillbirths per 1000 total births while neonatal death rates were calculated as number of neonatal deaths per 1000 live births. Birth weight was measured within 24 hours of birth by using either a pan spring or a pan digital weighing scale. LBW infants had birth weight less than 2500 grams.

Statistical Analyses

Data were analyzed using the STATA 13.1 statistical package (Stata Corp, College Station, Texas, USA). Study variables of interest and their combinations, with years of enrollment were cross tabulated in Stata and were transformed to graphs either in Stata itself or in Microsoft Excel. Pearson's correlation coefficient was used to study correlation between hemoglobin concentrations and BMI values and was tested for significance. A univariate multinomial logistic regression, adjusting for clustering was used to identify association between the WHO levels of

For peer review only

anemia and BMI categories. Significance of trends over the progression of years of enrollment in any of the study variables was determined using Cuzick's non-parametric test [17] for trend across ordered groups. The year-wise rates of the outcomes (CS, pregnancy related complication and LBW) were determined for each level of anemia and category of BMI. Similarly, for stillbirths and neonatal deaths the rates were calculated per 1000 total births and per 1000 live births respectively. The risks of association of maternal demographic characteristics and study outcomes were studied using multivariable regression analyses solved with generalized estimating equations (GEE), adjusting for clustering within the PHCs.

For the first set of regression models, explanatory variables included demographic characteristics - mother's age, education level and parity, along with levels of anemia and categories of BMI, as independent variables while dependent variables included all 5 study outcomes. BMI and anemia were analyzed as ordinal variables to be consistent with standard public health practices and management guidelines. A second set of models used demographic characteristics along with the constructed anemia-BMI variable as explanatory and modeled 3 post-natal outcomes– caesarian section stillbirths, neonatal death and LBW.

RESULTS

Participants

During the study period, 72,750 women had delivery outcomes and complete follow up through day 42 post-partum was completed. Women with invalid or missing data on BMI or hemoglobin (N=764) and miscarriage/MTP/multiple gestation (N=4412) were excluded.

For peer review only - http://bmjopen.bmj.com/site/about/guidelines.xhtml

Baseline maternal characteristics

Almost 91% of the included women were anemic (65811/72750) and over a third were underweight (25571/72750, 35.1%) (Table 1). The gestational age at the first antenatal visit was 6 to < 20 weeks for 72%, 20-28 weeks for 15% and > 28 weeks for 13% of the study population. Stratified analysis according to gestational age categories did not reveal any significant difference in the outcomes for those who had their first ANC visit during week 6-20 vs. at any time during pregnancy. We found a highly significant correlation(r= 0.2; P<0.001) between hemoglobin and BMI. Anemia was severe in less than 0.2% and moderate in nearly 48% women.

Multinomial logistic regression with normal hemoglobin concentrations as reference, showed highly potent (P<0.001) associations between women who were underweight and those with moderate/severe anemia (Risk Ratio (RR):1.9, 95% Confidence Interval (CI):1.6-2.2) as well as those with mild anemia (RR:1.3, 95% CI:1.2-1.5). Our study population mostly aged 20-29 years (93%); about half were primiparous 48% vs. multiparous, 52%.

As shown in Table 1, over time there was an increase in pregnant women in the older age groups>29 over and those who completed higher levels of education in later years (2015-2016). For parity, there was a reduction in proportions of parity>2 in 2016. The levels of anemia showed no significant changes over time. The proportion of women who were underweight (28% to 38%) increased over time (Figure 1). Anemia and low BMI increased over time (Figure 2).

For peer review only

Table 1: Baseline Characteristics of women in the study, by year of enrollment

Characteristics	Years								
	2009 N (%)	2010 N (%)	2011 N (%)	2012 N (%)	2013 N (%)	2014 N (%)	2015 N (%)	2016 N (%)	Total N (%)
	6433 (100)	10447 (100)	9626 (100)	9905 (100)	9496 (100)	9454 (100)	10108 (100)	7281 (100)	72750 (100)
Mother's Age (years)*									
<20	131 (2)	209 (2)	181 (1.9)	188 (1.9)	190 (2)	193 (2)	209 (2.1)	157 (2.2)	1458 (2)
20-29	6044 (94)	9867 (94.4)	9056 (94.1)	9269 (93.6)	8834 (93)	8743 (92.5)	9266 (91.7)	6640 (91.2)	67719 (93.1)
>29	258 (4)	371 (3.6)	389 (4)	448 (4.5)	472 (5)	518 (5.5)	633 (6.3)	484 (6.6)	3573 (4.9)
Mother's Education (grades)*									
Primary or Less (<=4)	1476 (22.9)	2281 (21.8)	1846 (19.2)	1927 (19.5)	1882 (19.8)	693 (7.3)	633 (6.3)	329 (4.5)	11067 (15.2)
Secondary(5-10)	3814 (59.3)	6210 (59.4)	5818 (60.4)	5880 (59.4)	5432 (57.2)	4962 (52.5)	5101 (50.5)	3522 (48.4)	40739 (56)
University(>10)	1139 (17.7)	1956 (18.7)	1958 (20.3)	2071 (20.9)	2172 (22.9)	3768 (39.9)	4358 (43.1)	3415 (46.9)	20837 (28.6)
Missing	4 (0.1)	0 (0)	4 (0)	27 (0.3)	10 (0.1)	31 (0.3)	16 (0.2)	15 (0.2)	107 (0.1)
Parity*									
Nulliparous	2905 (45.2)	5118 (49)	4706 (48.9)	4817 (48.6)	4384 (46.2)	4530 (47.9)	4967 (49.1)	3598 (49.4)	35025 (48.1)
1-2	3300 (51.3)	5032 (48.2)	4669 (48.5)	4827 (48.7)	4907 (51.7)	4721 (49.9)	4888 (48.4)	3544 (48.7)	35888 (49.3)
>2	228 (3.5)	297 (2.8)	249 (2.6)	261 (2.6)	204 (2.1)	197 (2.1)	234 (2.3)	137 (1.9)	1807 (2.5)
Missing	0 (0)	0 (0)	2 (0)	0 (0)	1 (0)	6 (0.1)	19 (0.2)	2 (0)	30 (0)
Anemia (gm/dl)									
Moderate/Severe(<10)	2962 (46)	5283 (50.6)	4620 (48)	4730 (47.8)	4682 (49.3)	4418 (46.7)	5058 (50)	3457 (47.5)	35210 (48.4)
Mild(10<11)	2743 (42.6)	4341 (41.6)	4033 (41.9)	4337 (43.8)	4006 (42.2)	4152 (43.9)	4041 (40)	2948 (40.5)	30601 (42.1)
Normal(>=11)	728 (11.3)	823 (7.9)	973 (10.1)	838 (8.5)	808 (8.5)	884 (9.4)	1009 (10)	876 (12)	6939 (9.5)
Body Mass Index (kg/m^2) *									
Underweight(<18.5)	1784 (27.7)	3537 (33.9)	3391 (35.2)	3450 (34.8)	3411 (35.9)	3424 (36.2)	3786 (37.5)	2788 (38.3)	25571 (35.1)
Normal(18.5-24.9)	4426 (68.8)	6558 (62.8)	5931 (61.6)	6061 (61.2)	5699 (60)	5619 (59.4)	5789 (57.3)	4095 (56.2)	44178 (60.7)
Overweight or Obese(≥25)	223 (3.5)	352 (3.4)	304 (3.2)	394 (4)	386 (4.1)	411 (4.3)	533 (5.3)	398 (5.5)	3001 (4.1)

* Highly significant (P<0.001) non parametric trend over years of enrollment

For peer review only

Maternal Mortality

There were 29 maternal deaths in the study population, 20 of whom had severe anemia. Moderate/Severe anemia was identified as a primary cause of death in 1 and as a co-morbid condition in 19. Of the total 29 deaths, anemia and underweight was observed in 8, anemia alone in 19, underweight alone in 1, and overweight/obese in 2. Reasons for maternal deaths, were hemorrhage (n=9, 31%), preeclampsia/eclampsia (n=7, 24%), infection (n=6, 21%), other/missing (n=7, 22%).

Study outcomes by years of enrollment

Overall, across all years, the rates of women who had CS or pregnancy related complications at delivery increased for those with anemia (Table 2). In contrast, stillbirths, neonatal deaths and LBW babies showed increased with greater severity of anemia. The rates of CS and pregnancy related complications increased with increasing BMI. Stillbirths, neonatal deaths and rates of LBW babies increased over time in underweight and decreased in overweight/obese mothers. Rates of CS increased for all levels of anemia as well as BMI, over time.

For peer review only

Table 2: Rates of study with respect to levels of anemia and body mass index categories, by year of enrolment

	Year of Enrollment								
	**2009**	**2010**	**2011**	**2012**	**2013**	**2014**	**2015**	**2016**	**Total**
**Variables**	**Rates of women experiencing cesarean sections**								
Anemia (gm/dl)									
Moderate/Severe(<10)	15.67	16.83	17.19	19.53	20.91	23.74	24.73	28.67	20.85
Mild(10-11)	16	17.97	19.69	21.51	24.84	26.42	31.08	33.58	23.8
Normal(>=11)	18.41	26.12	24.87	26.13	31.44	32.01	37.07	39.61	29.8
Body Mass Index (kg/m^2)									
Underweight(<18.5)	15.08	15.13	16.13	17.65	19.2	21.61	23.38	26.79	19.5
Normal(18.5-24.9)	15.95	18.65	19.91	21.53	24.64	26.87	29.9	33.41	23.6
Overweight or Obese(≥25)	27.8	35.8	33.55	41.12	43.78	43.55	49.72	53.52	42.59
**Variables**	**Rates of women with pregnancy complications present at delivery**								
Anemia (gm/dl)									
Moderate/Severe(<10)	14.58	14.1	12.03	14.76	14.72	10.5	10.7	10.85	12.78
Mild(10<11)	14.51	14.67	14.95	15.75	17.05	13.97	11.9	12.48	14.49
Normal(>=11)	16.62	21.51	16.65	17.42	22.15	18.1	15.86	14.27	17.73
Body Mass Index (kg/m^2)									
Underweight(<18.5)	13.85	12.58	11.53	14.03	14.07	10.25	10.46	10.26	12.04
Normal(18.5-24.9)	14.91	15.46	14.37	15.38	17.07	13.54	11.99	12.43	14.48
Overweight or Obese(≥25)	19.73	28.41	25.66	28.17	25.39	22.38	17.26	18.34	22.93
**Variables**	**Stillbirth rate per 1000 total births**								
Anemia (gm/dl)									
Moderate/Severe(<10)	29.37	25.55	22.29	23.89	26.27	21.05	23.33	26.61	24.54
Mild(10-11)	27.34	24.65	20.58	19.37	23.71	19.27	18.81	15.6	21.11
Normal(>=11)	12.36	18.23	15.42	16.71	16.09	16.97	14.87	15.98	15.85
Body Mass Index (kg/m^2)									
Underweight(<18.5)	29.15	25.73	22.41	22.32	25.21	20.74	22.45	27.62	24.05
Normal(18.5-24.9)	25.76	24.7	19.9	20.13	24.21	19.93	19.52	17.34	21.5
Overweight or Obese(≥25)	22.42	11.36	23.03	30.46	18.13	12.17	20.64	10.05	18.33
**Variables**	**Neonatal death rate per 1000 live births**								
Anemia (gm/dl)									
Moderate/Severe(<10)	22.06	21.63	19.64	28.74	24.05	21.99	24.05	21.89	23.09
Mild(10<11)	17.54	16.08	15.44	16.74	19.03	18.26	17.72	16.47	17.15
Normal(>=11)	11.25	25.38	11.62	14.8	6.33	12.82	13.25	9.37	13.06
Body Mass Index (kg/m^2)									
Underweight(<18.5)	21.23	20.13	21.26	23.68	24.98	20.09	22.94	20.74	21.96
Normal(18.5-24.9)	18.19	19.28	14.14	21.86	18.13	19.25	19.04	16.42	18.38
Overweight or Obese(≥25)	13.95	20.53	27.68	15.96	13.37	17.54	17.54	18.13	17.97
**Variables**	**Rates of low birth weight (<2500 g) babies**								
Anemia (gm/dl)									
Moderate/Severe(<10)	17.42	17.87	17.51	18.46	19.14	19.04	20.42	22.65	19.01
Mild(10-11)	16.99	14.37	14.63	13.53	14.5	14.72	16.98	18.69	15.35
Normal(>=11)	14.15	15.43	12.85	11.22	10.4	12.9	16.45	16.32	13.78
Body Mass Index (kg/m^2)									
Underweight(<18.5)	19.79	18.63	17.9	19.59	20.14	19.28	21.61	22.74	19.92
Normal(18.5-24.9)	15.95	15.23	14.69	13.81	14.6	15.09	17.29	19.54	15.61
Overweight or Obese(≥25)	11.66	10.51	15.13	10.41	10.88	14.11	12.38	10.8	11.96

For peer review only

Study Outcomes: Regression

The rates of CS and pregnancy related complications during delivery were significantly higher in non-anemic women vs. anemic women while higher risk of stillbirths, neonatal deaths and LBW was associated with increasing severity of anemia (Table 3). Risks of CS and pregnancy related complications during delivery were higher in overweight/obese women. The rates of neonatal deaths and LBW were significantly higher in underweight women (Table 3).

Mother's age (> 29 years), lower education level (secondary or less), and nulliparity were associated with stillbirth, neonatal deaths and LBW. Women < 20 years were at lower risk for CS and pregnancy related complications. Women with lower levels of education were also significantly less likely to have a CS or pregnancy related complication. Nulliparous women had a higher risk of CS and were twice as likely to experience pregnancy related complications at birth. Though parity of more than two lowered the risk of CS and pregnancy related complications, it increased the risk for neonatal deaths (Table 3). The risks of stillbirth, neonatal deaths and LBW were the highest when women were both anemic and underweight (Table 4).

For peer review only

Table 3: Risk factors for study outcomes, multivariable estimates

	Study Outcomes				
**Characteristics**	**Cesarean Sections<br>N=16693, 23%<br>RR(95% CI)^**	**Pregnancy related Maternal complications<br>N=10163, 14%<br>RR(95% CI)^**	**Stillbirths<br>N=1620, 2.2%<br>RR(95% CI)^**	**Neonatal deaths (within 28 days of birth)<br>N=1368, 1.9%<br>RR(95% CI)^**	**Low birth weight (<2500 gms)<br>N=12347, 17%<br>RR(95% CI)^**
Mother's Age (years)					
<20	0.80**(0.72 - 0.90)	0.83*(0.73 - 0.95)	0.88(0.62 - 1.25)	1.28(0.94 - 1.75)	1.04(0.92 - 1.16)
20-29	ref	ref	Ref	ref	ref
>29	1.60**(1.50 - 1.70)	1.38**(1.26 - 1.50)	1.76**(1.45 - 2.12)	1.47**(1.18 - 1.83)	1.28**(1.18 - 1.38)
Mother's Education (grades)					
Primary or Less (<=4)	0.47**(0.44 - 0.50)	0.69**(0.64 - 0.74)	1.62**(1.38 - 1.89)	1.49**(1.26 - 1.77)	1.14**(1.08 - 1.21)
Secondary(5-10)	0.68**(0.66 - 0.70)	0.89**(0.85 - 0.93)	1.38**(1.22 - 1.57)	1.30**(1.14 - 1.49)	1.14**(1.09 - 1.19)
University(>10)	ref	ref	Ref	ref	ref
Parity					
Nulliparous	1.38**(1.34 - 1.43)	2.00**(1.88 - 2.12)	1.31**(1.18 - 1.45)	1.48**(1.33 - 1.66)	1.35**(1.30 - 1.41)
1-2	ref	ref	Ref	ref	ref
>2	0.45**(0.38 - 0.53)	0.76*(0.64 - 0.90)	1.22(0.93 - 1.62)	1.52*(1.14 - 2.04)	1(0.89 - 1.13)
Anemia (gm/dl)					
Severe/Moderate (<10)	0.87**(0.83 - 0.92)	0.89**(0.84 - 0.95)	1.43**(1.17 - 1.75)	1.67**(1.33 - 2.08)	1.26**(1.18 - 1.35)
Mild(10>11)	0.90**(0.86 - 0.94)	0.90*(0.85 - 0.96)	1.28*(1.05 - 1.57)	1.28*(1.02 - 1.60)	1.08*(1.01 - 1.15)
Normal(>=11)	ref	ref	Ref	ref	ref
Body Mass Index (kg/m^2)					
Underweight(<18.5)	0.85**(0.82 - 0.88)	0.85**(0.81 - 0.88)	1.1(1.00 - 1.22)	1.12*(1.00 - 1.25)	1.21**(1.16 - 1.25)
Normal(18.5-24.9)	ref	ref	Ref	ref	ref
Overweight or Obese(≥25)	1.57**(1.48 - 1.67)	1.48**(1.36 - 1.60)	0.92(0.70 - 1.20)	1.11(0.84 - 1.46)	0.84*(0.76 - 0.94)

* P<0.05, ** P<0.001

^ Obtained by multivariable GEE regression models for each outcome, separately

For peer review only

Table 4: Anemia-BMI factors for study outcomes, multivariable estimates

	Study Outcomes		
**Characteristics**	**Stillbirths**<br><br>**N=1620, 2.2%**<br>**RR(95% CI)^**	**Neonatal deaths (within 28 days of birth)**<br>**N=1368, 1.9%**<br>**RR(95% CI)^**	**Low birth weight (<2500 gms)**<br>**N=12347, 17%**<br>**RR(95% CI)^**
Mother's Age (years)			
<20	0.88(0.62 - 1.25)	1.28(0.94 - 1.75)	1.04(0.93 - 1.16)
20-29	ref	Ref	ref
>29	1.75**(1.45 - 2.11)	1.48**(1.19 - 1.84)	1.27**(1.17 - 1.37)
Mother's Education (grades)			
Primary or Less (<=4)	1.64**(1.40 - 1.91)	1.52**(1.29 - 1.80)	1.16**(1.10 - 1.23)
Secondary(5-10)	1.40**(1.23 - 1.58)	1.32**(1.16 - 1.51)	1.15**(1.10 - 1.20)
University(>10)	ref	Ref	ref
Parity			
Nulliparous	1.30**(1.18 - 1.44)	1.47**(1.31 - 1.64)	1.35**(1.29 - 1.40)
1-2	ref	Ref	ref
>2	1.24(0.94 - 1.63)	1.56*(1.17 - 2.09)	1.02(0.91 - 1.15)
Anemia(gm/dl)-BMI(kg/m^2) factors			
Anemic(<11)-Normal(>=18.5)	1.30*(1.04 - 1.63)	1.54**(1.19 - 2.00)	1.22**(1.13 - 1.32)
Anemic(<11)-Underweight(<18.5)	1.47**(1.17 - 1.85)	1.74**(1.33 - 2.26)	1.49**(1.37 - 1.62)
Non-Anemic(>=11- Normal(>=18.5)	ref	Ref	ref
Non-Anemic(>=11)-Underweight(<18.5)	0.96(0.62 - 1.48)	1.37(0.88 - 2.13)	1.37**(1.20 - 1.57)

* P<0.05, ** P<0.001

^ Obtained by univariate GEE regression models for each outcome, separately

For peer review only

DISCUSSION

Maternal malnutrition continues to be a silent emergency particularly in low middle income countries (LMICs) like India. We attempted to understand through present study, not only the trends in the prevalence of anemia and low BMI over the eight years but the less understood combination of low maternal BMI and anemia with pregnancy related outcomes among rural women from Eastern Maharashtra. We found that more than 90% of the pregnant women were anemic and 35% were underweight (BMI< 18.5). It was alarming that the rates of anemia remained unchanged over eight years (2009-2016). The majority of women studied had mild to moderate anemia, with severe anemia in less than 0.2%. Rates of underweight increased by 8% over eight years, and there was an increase in the rates of overweight/obese from 2012 to 2016. Anemia and increased the risk of stillbirths, neonatal deaths and LBW. The risk increased further if both conditions were present.

The NFHS data (NFHS-3 and NFHS-4) found anemia prevalence rate of 54% between 2005 and 2016, with a minimal reduction in Maharashtra between NFHS-3(58%) to NFHS-4 (49%).[10,18] Disturbingly, our estimates are higher than the District Level Household Survey (DLHS-4, 2012-2013) which reported a rate between 43% and 49% among pregnant women aged 15-49 years in the Nagpur, Bhandara and Wardha districts while the rates were not reported for the Chandrapur district.[19] The women in our study were young (20-29 years), from both rural and backward tribal regions known for high prevalence of micronutrient deficiency specifically iron deficiency anemia and other causes of anemia such as sickle cell anemia, hookworm infestation and malaria. [20-25] Another limiting factor in our study was that we were

For peer review only - http://bmjopen.bmj.com/site/about/guidelines.xhtml

not able to evaluate likely confounders of adverse outcomes in pregnancy such as socio-economic status, number and timing of ANC visits and use of tobacco, etc. as these data were not collected in the MNH registry. Previous studies from eastern Maharashtra have reported similar high rates of anemia. [26-29]Variations in rates of anemia may be due to methods used for surveying and assessing hemoglobin levels.[30-31] Overall rates of anemia in pregnant women remain unchanged over the eight years of study although there was some reduction in rates of severe anemia possibly related to irregular and poor coverage of the national iron supplementation program [32-34] and suboptimal compliance. Indian national surveys show poor compliance with iron supplementation during pregnancy (<30%), [10,35] due to side effects, forgetfulness, and false beliefs about the importance of anemia in pregnancy.[36-38] Directly observed administration of iron supplementation may improve compliance and is being implemented for adolescent girls in India.[39,40] We believe it should be considered in pregnant women.

However, we note that in our study, rates of severe anemia decreased over the span of eight years possibly related to improved attendance of ante-natal care visits, early detection and current management guidelines using injectable iron for treatment of severe anemia at the public health facilities from the 12th Five year plan.[41]

Our study reports over a third of the study participants to be underweight during pregnancy, which is consistent with the findings of NFHS for rural Nagpur.[10]We also found an 8% increase in low BMI in pregnant women between 2009 and 2016. There was also a trend towards

For peer review only

BMI from the normal to overweight/obese categories over this same time period as has been observed.[42]

Anemia and underweight co-existed among one-third of the study participants which increased over time This change may be due to increasing prevalence of underweight as the rates of anemia did not change over the study period. The most probable cause for maternal underweight in this region would be malnutrition.[43,44] It is also likely that iron and other micronutrient deficiency were responsible for higher rates of anemia in underweight women [45-47] but this should be studied further to reduce rates of anemia in the community.

The rates of CS increased over the years consistent with an increase in facility based deliveries in India.[48] However, anemia at any level reduced the likelihood of CS and pregnancy related complication and underweight and anemic women are more likely to have fetal growth retardation and smaller babies.[49-52] Pregnancy related complication in our study population are most commonly due to obstructed labor and toxemia of pregnancy, both of which are less likely to occur in underweight and anemic women who tend to have smaller babies.[47] Women with higher BMI (who are less likely to be anemic are at increased risk of miscarriage, gestational diabetes, preeclampsia, venous thromboembolism, induced labour, caesarean section, anaesthetic complications and wound infections.[48]

Underweight and anemia significantly increased the risk of LBW and is consistent with other Indian studies[11]. .The risk of having LBW babies in women who were underweight also increased by 20%, consistent with other studies that have reported an association of maternal

For peer review only

anthropometry and size of the baby. [2,3] A study in Tanzania, on 12,269 newborns, identified short maternal stature as an significant risk factor for SGA and preterm-appropriate for gestational age.[53] A recent study from Mexico reported that higher proportions of SGA neonates were born to malnourished and anemic mothers than the mothers who were either malnourished or anemic.[54] This is consistent with our data. We also note that rates of LBW increased over the years in women who had moderate/severe anemia and in women who were underweight. Of note LBW babies are at increased risk of becoming stunted adults which will expound the inter-generational burden of LBW. [55]

Anemia also significantly increased the risk of stillbirths. It increased by 28% and 43% respectively in women who were mildly anemic and moderate/severely anemic as compared to non-anemic women. While there are studies to support our findings on the association of maternal anemia on stillbirths,[11,56,57] there is a need to understand the effect of maternal underweight *per se* in the developing countries. Anemia could also reflect poor socio-economic conditions and limited access to quality intra-partum care which resulted in stillbirths. For example, sickle cell anemia, hookworm infestation and malaria, all common in Eastern Maharashtra may have contributed to higher rates of stillbirths, [20-25]as does ethnicity in underweight women previously.[58]

There is a paucity of data on the reason for an association between anemia and neonatal mortality.[55, 59] We contribute to bridging this gap by analyzing 1638 infant deaths based on presence of pre-natal anemia among the mothers, or not. We found that the rates of neonatal deaths were nearly 1.4 times more in women with mild anemia as compared to who were non

For peer review only

anemic and this risk further increased to 1.7 times for moderate/severe anemic women. This is consistent with the finding of a recent meta-analysis in LMIC that found the odds of neonatal mortality to be 2.7 times among the anemic mothers[60] based on one study from India and another from Malawi usingthe outcomes of 447 and 795 pregnant women, respectively. Women with anemia are also more likely to deliver newborns that are low birth weight and that may be a reason for the higher risk of mortality. [61]

Neonatal death rates were highest in underweight mothers as has been reported previously.[60]A recent meta-analysis shows a J shaped association between neonatal deaths and maternal BMI [62,63]. Low maternal BMI and anemia are associated with LBW and preterm deliveries which are independent risk factors for infant mortality in India[64,65].

CONCLUSIONS AND PUBLIC HEALTH IMPLICATIONS

The combination of anemia and underweight in pregnancy increased the risk of stillbirths, neonatal deaths and delivering LBW babies. These findings raise major concerns because national programs to address iron deficiency anemia have not reduced the rates of anemia among the rural pregnant women in eastern Maharashtra over past 8 years. There has been a steady increase in prevalence of underweight in pregnancy with almost a third of pregnant women being both anemic and underweight. Immediate and effective public health interventions are needed to address maternal anemia and malnutrition to improve birth outcomes. There is an urgent need for evaluation and addressing of gaps of the iron supplementation program for adolescent girls and

For peer review only

pregnant women in these areas with high rates of anemia, in addition to a more holistic approach to curb maternal malnutrition. Meanwhile, seeking simultaneously short term strategies such as referral to a better healthcare service and management of delivery for the segment of pregnant population who are both anemic and underweight could be helpful in reducing neonatal mortality. Such actions are imperative to break the intergenerational cycle of poor growth in the offspring and also for improving child survival.

LIST OF ABBREVIATIONS

ANM - Auxiliary Nurse Midwife

APH - Antepartum Hemorrhage

BMI - Body Mass Index

CI - Confidence Interval

CS - Cesarean Section

DLHS - District Level Household Survey

GEE - Generalized Estimating Equations

GN - Global Network

IFA - Iron Folic Acid

IRB - Institutional Review Board

LBW - Low Birth Weight

LMIC - Low and Middle Income Countries

LMRF - Lata Medical Research Foundation

For peer review only

MNHR - Maternal and Newborn Health Registry

MTP - Medical Termination of Pregnancy

NFHS - National Family and Health Survey

NICHD - *Eunice Kennedy Shriver* National Institute of Child Health and Human Development

PHC - Primary health Center

PPH - Postpartum Hemorrhage

RA - Registry Administrator

RR - Risk Ratio

SGA - Small for Gestational Age

USA - United States of America

WHO - World Health Organization

AUTHOR CONTRIBUTIONS

AP conceived and designed the study, and developed the initial data collection tools; AP and AAP wrote the first draft of the manuscript, which PLH, PKD, YVP and SG subsequently revised. AP and AAP developed the analysis plan outline and AAP carried out the data analyses. AP, PLH, PKD, YVP and SG contributed to data analysis through review, interpretation and cross-checking figures and tables. All authors read, revised, and approved the final manuscript.

For peer review only

ACKNOWLEDGEMENTS

We are deeply indebted to Research Triangle International, Durham, USA for their enthusiastic support. Thanks and recognition are due to the Registry Administrators at the Nagpur site of the MNHR who collect data, often in very challenging circumstances, and the communities, families, and mothers who agree to participate in the MNHR. We express our sincere gratitude to Dr. Vaishali Khedikar, Dr. Savita Bhargav and Dr, Kunal Kurhe at LMRF, who helped immensely by strengthening the manuscript through their inputs.

DATA SHARING

A de-identified minimal dataset that underlies the findings and conclusions described in the manuscript can be shared upon request by the editors to verify the reported study findings.All authors hereby declare that individual participant data in any form is not a part of this publication.

FUNDING

This study was funded by the *Eunice Kennedy Shriver* National Institute of Child Health and Human Development of the US National Institutes of Health funded this study (Grants Reference Nos. U01HD058322 and U01HD078439).

For peer review only

ETHICS

The study in Nagpur, India has been reviewed and approved by the Institutional Review Board (IRB) of the Lata Medical Research Foundation (RPC#22A)and the Partners Human Research Committee/IRB in Boston, MA (H-35430).

CONSENT TO PARTICIPATE

The study was explained thoroughly and in detail, to the women who lived in the participating communities. The women were enrolled in the study only after giving informed consent. An IRB approved consent form was used for this purpose.

COMPETING INTERESTS

None declared

TRANSPARENCY

The lead author(ABP) affirms that the manuscript is an honest, accurate, and transparent account of the study being reported; that no important aspects of the study have been omitted; and that any discrepancies from the study as planned have been explained.

For peer review only

What is already known on this topic

Low body mass Index (BMI) and anemia during pregnancy have been independently identified as risk factors for delivering pre-term, small for gestational age (SGA) and LBWs, however their relationship with respect to neonatal mortality and stillbirth remains inconclusive. Also, their relationship of severity of anemia with adverse pregnancy outcomes is not well established for low middle income countries (LMIC). The risk arising out of co-existence of anemia and low BMI -a common scenario in LMIC like India, on poor maternal has not been characterized.

What this study adds

This study showed a high prevalence of anemia (>90%) and underweight (35%) among rural pregnant women in eastern Maharashtra. The rate of anemia remained unaltered but there was an increase in the underweight and overweight/obese woman over the past 8 years from 2009-2016. This highlights the magnitude of persistent public health problem of nutrition origin despite the national programs in place and also the emerging dual burden of diseases in the rural areas as well.

Anemia and underweight during pregnancy were individually found to increase the risk of stillbirths, neonatal deaths and LBW. Women with moderate-severe anemia were at greater risk as compared to the mildly anemic women. These risks increased further if anemia and underweight were present together during pregnancy. This raises concerns as the two conditions often co-exist in LMICs and in the present study was, as high as, affecting a third of the study population.

Recommendations for policy

There is urgent need to address maternal malnutrition. Though holistic approach would be eradicating maternal malnutrition through more targeted and intensive food or medicinal supplement based approach, seeking short term strategies such as referral to a better healthcare service simultaneously could help in breaking the intergenerational cycle of poor growth in the offspring and reducing child mortality.

For peer review only

REFERENCES:

1. Black RE, Victora CG, Walker SP, et al. Maternal and child undernutrition and overweight in low-income and middle-income countries.*Lancet*2013; 382(9890): 427-51. doi: 10.1016/S0140-6736(13)60937-X. (accessed 30 November, 2017).
2. Bhutta ZA, Das JK, Rizvi A, et al. Evidence-based interventions for improvement of maternal and child nutrition: what can be done and at what cost? *Lancet* 2013; 382(9890): 452-77. doi:10.1016/S0140-6736(13)60996-4.(accessed 30 November, 2017).
3. Black RE, Allen LH, Bhutta ZA, et al. Maternal and child undernutrition: global and regional exposures and health consequences. *Lancet* 2008; 371(9608):243-60. doi: 10.1016/S0140-6736(07)61690-0.(accessed30 November, 2017).
4. United Nations Development Program.*Sustainable Development Goals*. http://www.undp.org/content/undp/en/home/sustainable-development-goals.html (accessed 30 November 2017).
5. World Health Organization. *Global nutrition targets 2025: policy brief series (WHO/NMH/NHD/14.2).2014*.http://apps.who.int/iris/bitstream/10665/149018/1/WHO_NMH_NHD_14.2_eng.pdf?ua=1(accessed on 11 Sept 2017).
6. Government of India. *Millennium Development Goals: India Country Report 2015*. http://mospi.nic.in/sites/default/files/publication_reports/mdg_2july15_1.pdf(accessed on 11 Sept 2017).
7. United Nations Inter-agency Group for Child Mortality. *Levels & Trends in Child Mortality: Report 2015*. https://www.unicef.org/publications/files/Child_Mortality_Report_2015_Web_9_Sept_15.pdf(accessed on 30 Nov 2017).
8. United Nations Children's Fund. *Committing to Child Survival: A promise renewed. Progress Report 2015*. http://www.apromiserenewed.org/wp-content/uploads/2015/09/APR_2015_8_Sep_15.pdf(accessed on 30 Nov 2017).
9. Van Der Linden EL, Browne JL, Vissers KM, et al. Maternal body mass index and adverse pregnancy outcomes: A Ghanaian cohort study. *Obesity* 2016; 24(1): 215-22. doi:10.1002/oby.21210. (accessed on 30 Nov 2017).

10. International Institute of Population Sciences. *National Family Health Survey-4: Maharashtra State Fact Sheet*. http://rchiips.org/nfhs/pdf/NFHS4/MH_FactSheet.pdf (accessed 30 November 2017).
11. NairM,ChoudhuryMK,Choudhury SS, et al. Association between maternal anemia and pregnancy outcomes: a cohort study in Assam, India. *BMJ Glob Health* 2016;1:e000026. doi:10.1136/bmjgh-2015-000026.(accessed30th November 2017).
12. Haider BA, Olofin I, Wang M, et al.Anemia, prenatal iron use, and risk of adverse pregnancy outcomes: systematic review and meta-analysis.*BMJ* 2013;346:f3443. doi:10.1136/bmj.f3443. (accessed30 November 2017).
13. Goudar SS, Carlo WA, McClure EM, et al. The maternal and newborn health registry study of the global network for women's and children's health research. *Int J GynaecolObstet* 2012; 118(3): 190-3. doi:10.1016/j.ijgo.2012.04.022
14. World Health Organization. *Global database on body mass index.* Http://apps.who.int/bmi/index.jsp?introPage=intro_3.html (accessed 30 November 2017)
15. U.S.Agency for International Development/ PATH. *A Manual for Health Workers: Anemia Detection Methods in Low-Resource Settings*;1997. http://pdf.usaid.gov/pdf_docs/PNACC882.pdf(accessed30 November 2017).
16. World Health Organization (WHO*). Preventing and controlling anemia through primary health care: A guide for health administrators and program managers*. Geneva: WHO; 1989. http://www.who.int/nutrition/publications/micronutrients/anaemia_iron_deficiency/9241542497.pdf (accessed 30 November 2017).
17. Cuzick J. A Wilcoxon-type test for trend. *Stat Med* 1985;4(1):87–90.
18. International Institute for Population Sciences (IIPS) and Macro International. *National Family Health Survey (NFHS-3), 2005–06, India, Volume I*. Mumbai: IIPS;2007. http://rchiips.org/nfhs/NFHS-3%20Data/VOL-1/India_volume_I_corrected_17oct08.pdf(accessed 1 December 2017).
19. International Institute of Population Sciences(IIPS). *District Level Household and Facility Survey -4. State Fact Sheet Maharashtra (2012-13).* http://rchiips.org/pdf/dlhs4/report/MH.pdf (accessed 22 November 2017).

For peer review only

20. World Health Organization. *The prevalence of anemia in women: a tabulation of available information (WHO/MCH/MSM/92)*. 2nd ed. Geneva: World Health Organization; 1992. http://apps.who.int/iris/bitstream/10665/58994/1/WHO_MCH_MSM_92.2.pd (accessed 30 November 2017)

21. Bharati S, Pal M, Som S, Bharati P. Temporal trend of anemia among reproductive-aged women in India. *Asia Pac J Public Health*2015;27(2):NP1193-1207.

22. Balarajan YS, Fawzi WW, Subramanian SV. Changing patterns of social inequalities in anemia among women in India: cross-sectional study using nationally representative data. *BMJOpen* 2013;3(3):e002233 (accessed 1 December 2017).

23. Kalaivani K. Prevalence & consequences of anemia in pregnancy. *Indian J Med Res* 2009;130(5):627–33.

24. Colah RB, Mukherjee MB, Martin S,et al. Sickle cell disease in tribal populations in India. *Indian J Med Res*2015;141(5):509-515. doi:10.4103/0971-5916.159492 (accessed 1 December 2017).

25. Corrêa G, Das M, KovelamudiR,et al. High burden of malaria and anemia among tribal pregnant women in a chronic conflict corridor in India. *Confl and Health 2017*; 11: 10. doi: 10.1186/s13031-017-0113-1 (accessed 1 December 2017).

26. Ahankari AS, Myles PR, Dixit JV,etal.Risk factors for maternal anemia and low birth weight in pregnant women living in rural India: a prospective cohort study. *Public Health* 2017 Oct;151:63-73. doi: 10.1016/j.puhe.2017.06.023(accessed 1 December 2017)

27. Arlappa N, Meshram II, BalakrishnaN,et al. Prevalence of anemia among different physiological groups in the rural areas of Maharashtra. *Indian J Community Health* 2014; 26(3):278-284.

28. Ahankari A, Bapat S, Myles P, et al. Factors associated with preterm delivery and low birth weight: a study from rural Maharashtra, India. *F1000Res*2017;6:72. doi: 10.12688/f1000research.10659.1 1 (accessed 1 December 2017).

29. Wadgave HV. Burden of anemia among the pregnant women in rural Area. Healthline 2011;2:76-7.http://www.iapsmgc.org/index_pdf/53.pdf (accessed 1 December 2017).

30. Balasubramaniam P, Malathi A. Comparative study of hemoglobin estimated by Drabkin's and Sahli'smethods.*J Postgrad Med* 1992; 38(1):8-9.

31. Rakesh PS. Prevalence of anemia in Kerala State, Southern India - a systematic review.*JClinDiagn Res* 2017;11(5):LE01-LE04. doi: 10.7860/JCDR/2017/24681.9951(accessed 1 December 2017).
32. Pasricha SR, Biggs BA, Prashanth NS, et al. Factors influencing receipt of iron supplementation by young children and their mothers in rural India: local and national cross-sectional studies. *BMC Public Health*2011;11:617. doi: 10.1186/1471-2458-11-617(accessed 1 December 2017).
33. Chaturvedi S, RanadiveB.Are we really making motherhood safe? A study of provision of iron supplements and emergency obstetric care in rural Maharashtra.*Natl Med J India* 2007; 20(6):294-6.
34. KumarV,Singh P. Insurance coverage under different health schemes in Uttar Pradesh, India. *ClinEpidemiol Glob Health*2017; 5(1): 1-7.http://www.ceghonline.com/article/S2213-3984(16)30011-2/fulltext(accessed 1 December 2017).
35. UNICEF. *National Factsheet, Coverage Evaluation Survey, 2009*. New Delhi: UNICEF India Country Office; 2010. http://www.indiawaterportal.org/sites/indiawaterportal.org/files/National%20Factsheet_Coverage%20Evaluation%20Survey_UNICEF_2009.pdf (accessed 25 November 2017).
36. Pal PP, Sharma S, SarkarTK,et al. Iron and folic acid consumption by the ante-natal mothers in a rural area of India in 2010. *IntJPrev Med* 2013;4(10):1213-1216.
37. Mithra P, Unnikrishnan B, Rekha T, et al. Compliance with iron-folic acid (IFA) therapy among pregnant women in an urban area of south India. *Afr Health Sci* 2013;13(4):880-885. 10.4314/ahs.v13i4.3(accessed 1 December 2017).
38. Galloway R, Dusch E, Elder L,etal.Women's perceptions of iron deficiency and anemia prevention and control in eight developing countries.*SocSci Med* 2002;55(4):529-44.
39. Kotecha PV, Nirupam S, Karkar PD. Adolescent girls' anemia control program, Gujarat, India. Indian J Med Res 2009;130(5):584-9.
40. Vir SC, Singh N, Nigam AK, et al. Weekly iron and folic acid supplementation with counseling reduces anemia in adolescent girls: A large-scale effectiveness study in Uttar Pradesh, India. *Food Nutr Bull*2008;29:186-94.

41. Bhatt B, Suryawanshi S, Mali B. "THE NATIONAL HEALTH OUTCOMES IN THE 12th FIVE YEAR PLAN AND THE GOALS THEREOF" International Journal of Application or Innovation in Engineering & Management (IJAIEM) 2014.l;3(3): 423-25

42. NCD Risk Factor Collaboration (NCD-RisC).Trends in adult body-mass index in 200 countries from 1975 to 2014: a pooled analysis of 1698 population-based measurement studies with 19.2 million participants. Lancet 2016;387(10026):1377-1396. doi: 10.1016/S0140-6736(16)30054-X(accessed 1 December 2017).

43. National Nutrition Monitoring Bureau (NNMB). Diet and nutritional status of rural population, prevalence of hypertension and diabetes among adults and infant and young child feeding practices-A report of third repeat survey. Technical report no. 26.Hyderabad: National Institute of Nutrition, Indian Council of Medical Research; 2012. http://nnmbindia.org/1_NNMB_Third_Repeat_Rural_Survey___Technicl_Report_26.pdf (accessed 1 December 2017).

44. Mahanta LB, Choudhury M, Devi A, et al. On the Study of Pre-Pregnancy Body Mass Index (BMI) and Weight Gain as Indicators of Nutritional Status of Pregnant Women Belonging to Low Socio-Economic Category: A Study from Assam. *Indian J Community Med* 2015;40(3):198-202. doi: 10.4103/0970-0218.158870 (accessed 1 December 2017).

45. Sumarmi S, Puspitasari N, Handajani R, et al. Underweight as a risk factor for iron depletion and iron-deficient erythropoiesis among young women in rural areas of East Java, Indonesia.*Malays J Nutr* 2016;22(2):219–32.

46. Hettiarachchi M, Liyanage C, Wickremasinghe R, et al.Prevalence and severity of micronutrient deficiency: a cross-sectional study among adolescents in Sri Lanka.*Asia Pac J ClinNutr* 2006;15(1):56-63.

47. Darnton-Hill I, Mkparu UC. Micronutrients in Pregnancy in Low- and Middle-Income Countries. *Nutrients* 2015;7(3):1744-1768. doi:10.3390/nu7031744 (accessed on 1 December 2017).
https://link.springer.com/article/10.1186/s13690-017-0181-0

48. Patel AB, Prakash AA, Raynes-Greenow C, et al. Description of inter-institutional referrals after admission for labor and delivery: a prospective population based cohort

study in rural Maharashtra, India.BMC Health Serv Res2017; 17(1):360. doi: 10.1186/s12913-017-2302-4 (accessed 1 December, 2017).

49. Sebastian T, Yadav B, Jeyaseelan L, et al. Small for gestational age births among South Indian women: temporal trend and risk factors from 1996 to 2010. *BMC Pregnancy and Childbirth*2015;15:7. doi:10.1186/s12884-015-0440-4 (accessed 1 December 2017).
50. Kaur M, Chauhan A, Manzar MD, et al. Maternal anemia and neonatal outcome: aprospective study on urban pregnant women. *J ClinDiagn Res* 2015;9(12):QC04-QC08. doi:10.7860/JCDR/2015/14924.6985 (accessed 1 December 2017).
51. Verma A, Shrimali L. Maternal body mass index and pregnancy outcome. J ClinDiagn Res 2012;6(9):1531-1533. doi:10.7860/JCDR/2012/4508.2551(accessed 1 December 2017).
52. Haider BA, Olofin I, Wang M, et al.Anemia, prenatal iron use, and risk of adverse pregnancy outcomes: systematic review and meta-analysis. *The BMJ*2013;346:f3443. doi:10.1136/bmj.f3443(accessed 1 December 2017).
53. Muhihi A, Sudfeld CR, Smith ER, et al. Risk factors for small-for-gestational-age and preterm births among 19,269 Tanzanian newborns. *BMC Pregnancy Childbirth* 2016;16:110. doi:10.1186/s12884-016-0900-5(accessed 1 December 2017).
54. Mahajan SD, Singh S, Shah P, et al.Effect of maternal malnutrition and anemia on the endocrine regulation of fetal growth.*Endocr Res* 2004; 30(2):189-203. Varela-Silva MI, Azcorra H, Dickinson F, Bogin B, Frisancho AR. Influence of maternal stature, pregnancy age, and infant birth weight on growth during childhood in Yucatan, Mexico: a test of the intergenerational effects hypothesis. *Am J Hum Biol* 2009; 21(5):657-63. doi: 10.1002/ajhb.20883 (accessed 6 December 2017)
55. Varela-Silva MI, Azcorra H, Dickinson F, et al. Influence of maternal stature, pregnancy age, and infant birth weight on growth during childhood in Yucatan, Mexico: a test of the intergenerational effects hypothesis. *Am J Hum Biol* 2009; 21(5):657-63. doi: 10.1002/ajhb.20883 (accessed 6 December 2017)
56. Tandu-Umba B, Mbangama AM. Association of maternal anemia with other risk factors in occurrence of Great obstetrical syndromes at university clinics, Kinshasa, DR Congo. *BMC Pregnancy Childbirth* 2015; *15:* 183. doi: 10.1186/s12884-015-0623-z(accessed 1 December 2017).

57. Ali AA, Adam I.Anaemia and stillbirth in Kassala Hospital, Eastern Sudan.*J Trop Pediatr* 2011;57(1):62-4.

58. Salihu HM, Mbah AK, Alio AP, et al.Maternalprepregnancy underweight and risk of early and late stillbirth in black and white gravidas.*JNatl Med Assoc*2009;101(6):582-7

59. Ramakrishnan U, Grant F, Goldenberg T, et al. Effect of women's nutrition before and during early pregnancy on maternal and infant outcomes: a systematic review.PaediatrPerinatEpidemiol, 26: 285–301. doi:10.1111/j.1365-3016.2012.01281.x (accessed 1 December 2017).

60. Rahman MM, Abe SK, Rahman MS, et al. Maternal anemia and risk of adverse birth and health outcomes maternal in low- and middle-income countries: systematic review and meta-analysis.*Am J ClinNutr*2016;103(2):495-504.

61. Rahmati S, Delpishe A, Azami M, Reza M,Ahmadi H andSayehmiriK.Maternal Anemia during pregnancy and infant low birth weight: A systematic review and Meta-analysis. Int J Reprod Biomed (Yazd). 2017 Mar; 15(3): 125–134.

62. Kalk P, Guthmann F, Krause K, et al.Impact of maternal body mass index on neonatal outcome.*Eur J Med Res* 2009 May 14; 14(5): 216-22.

63. Sankar MJ, Neogi SB, Sharma J, et al. State of newborn health in India. *J Perinato* 2016; 36(3), S3–S8. doi: 10.1038/jp.2016.183 (accessed 1 December 2017).

64. Aune D, Saugstad OD, Henriksen T, TonstadS.Maternal body mass index and the risk of fetal death, stillbirth, and infant death: a systematic review and meta-analysis.*JAMA*2014;311(15):1536-46.

65. Million Death Study Collaborators,Bassani DG, Kumar R, et al. Causes of neonatal and child mortality in India: nationally representative mortality survey. *Lancet*2010;376(9755):1853-60. doi: 10.1016/S0140-6736(10)61461-4 (accessed 1 December 2017).

Figure 1: Categorical anemia (gm/dl)in included women by body mass index (kg/m^2), over year of enrolment

Figure1: Pic

For peer review only

1 **Figure 2: Proportions of Anemia (gm/dl) & body mass index (kg/m^2) interaction in**
2 **included women, over year of enrollment**
3

4 **Figure2: Pic**

5

6

For peer review only

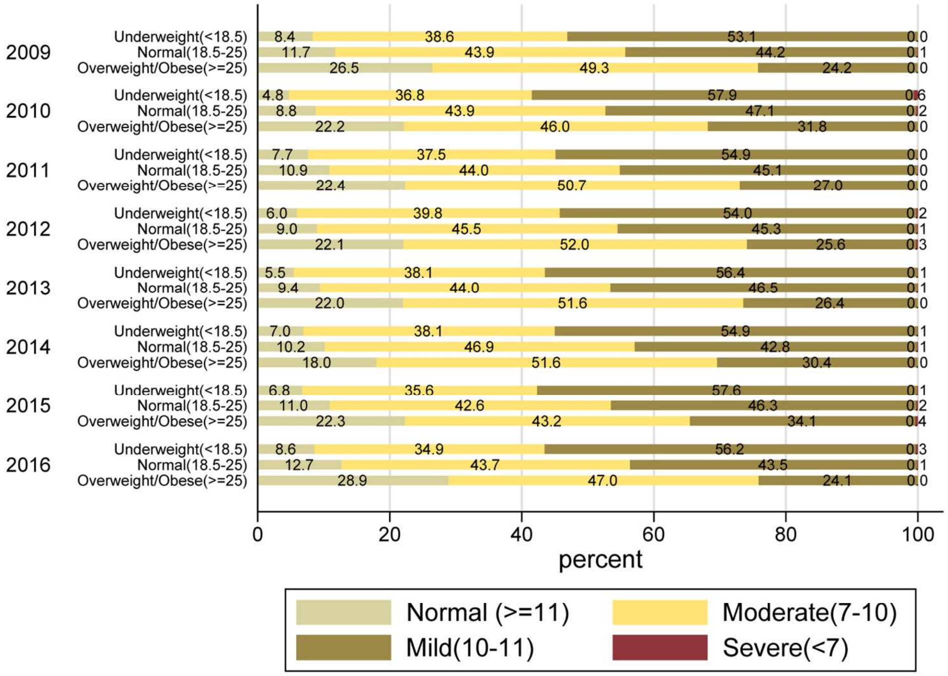

Figure 1: Categorical anemia (gm/dl)in included women by body mass index (kg/m2), over year of enrolment

139x101mm (300 x 300 DPI)

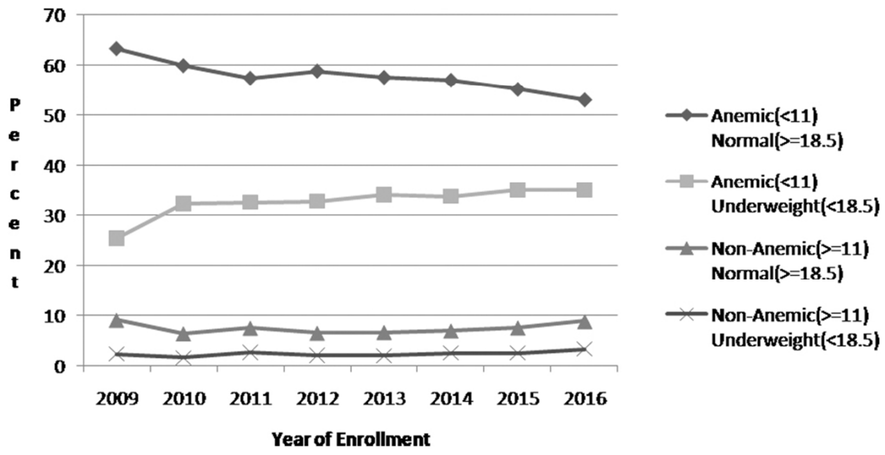

Figure 2: Proportions of Anemia (gm/dl) & body mass index (kg/m2) interaction in included women, over year of enrollment

101x49mm (300 x 300 DPI)

For peer review only

STROBE Statement—Checklist of items that should be included in reports of ***cohort studies***

	Item No	Recommendation
**Title and abstract**	1	(*a*) Indicate the study's design with a commonly used term in the title or the abstract ☑ - ***Page 1***
		(*b*) Provide in the abstract an informative and balanced summary of what was done and what was found ☑ - ***Page 5***
**Introduction**		
Background/rationale	2	Explain the scientific background and rationale for the investigation being reported ☑ - ***Page 8***
Objectives	3	State specific objectives, including any prespecified hypotheses ☑ - ***Page 8-9***
**Methods**		
Study design	4	Present key elements of study design early in the paper ☑ - ***Page 9***
Setting	5	Describe the setting, locations, and relevant dates, including periods of recruitment, exposure, follow-up, and data collection ☑ - ***Page 9***
Participants	6	(*a*) Give the eligibility criteria, and the sources and methods of selection of participants ☑ - ***Page 9-10***
		(*b*) For matched studies, give matching criteria and number of exposed and unexposed N/A
Variables	7	Clearly define all outcomes, exposures, predictors, potential confounders, and effect modifiers. Give diagnostic criteria, if applicable ☑ - ***Page 11 & 12***
Data sources/ measurement	8*	For each variable of interest, give sources of data and details of methods of assessment (measurement). Describe comparability of assessment methods if there is more than one group ☑ - ***Page 10 & 11***
Bias	9	Describe any efforts to address potential sources of bias ☑ - ***Page 10 & 11***
Study size	10	Explain how the study size was arrived at - N/A
Quantitative variables	11	Explain how quantitative variables were handled in the analyses. If applicable, describe which groupings were chosen and why ☑ - ***Page 10, 11 & 12***
Statistical methods	12	(*a*) Describe all statistical methods, including those used to control for confounding ☑ - ***Page 11 & 12***
		(*b*) Describe any methods used to examine subgroups and interactions ☑ - ***Page 12***
		(*c*) Explain how missing data were addressed ☑ - ***Page 12***
		(*d*) If applicable, describe analytical methods taking account of sampling strategy ☑ - ***Page 12***
		(*e*) Describe any sensitivity analyses - N/A
**Results**		
Participants	13*	(a) Report numbers of individuals at each stage of study—eg numbers potentially eligible, examined for eligibility, confirmed eligible, included in the study, completing follow-up, and analysed ☑ - ***Page 12 & 13***
		(b) Give reasons for non-participation at each stage - N/A
		(c) Consider use of a flow diagram - N/A
Descriptive data	14*	(a) Give characteristics of study participants (eg demographic, clinical, social) and information on exposures and potential confounders ☑ - ***Page 13***
		(b) Indicate number of participants with missing data for each variable of interest ☑ - ***Page 12 & 13***
Outcome data	15*	Report numbers of outcome events or summary measures ☑ - ***Page 15***
Main results	16	(*a*) Give unadjusted estimates and, if applicable, confounder-adjusted estimates and their

For peer review only - http://bmjopen.bmj.com/site/about/guidelines.xhtml

		precision (eg, 95% confidence interval). Make clear which confounders were adjusted for and why they were included ☑ - ***Page 17 thru 19***
		(*b*) Report category boundaries when continuous variables were categorized ☑ - ***Page 10 & 11***
		(*c*) If relevant, consider translating estimates of relative risk into absolute risk for a meaningful time period - N/A
Other analyses	17	Report other analyses done—eg analyses of subgroups and interactions, and sensitivity analyses ☑ - ***Page 17 & 19***
**Discussion**		
Key results	18	Summarise key results with reference to study objectives ☑ - ***Page 15 thru 19***
Limitations	19	Discuss limitations of the study, taking into account sources of potential bias or imprecision. Discuss both direction and magnitude of any potential bias ☑ - ***Page 7, 20-24***
Interpretation	20	Give a cautious overall interpretation of results considering objectives, limitations, multiplicity of analyses, results from similar studies, and other relevant evidence ☑ - ***Page 20 thru 24***
Generalisability	21	Discuss the generalisability (external validity) of the study results ☑ - ***Page 20 thru 24***
**Other information**		
Funding	22	Give the source of funding and the role of the funders for the present study and, if applicable, for the original study on which the present article is based ☑ - ***Page 27***

*Give information separately for exposed and unexposed groups.

Note: An Explanation and Elaboration article discusses each checklist item and gives methodological background and published examples of transparent reporting. The STROBE checklist is best used in conjunction with this article (freely available on the Web sites of PLoS Medicine at http://www.plosmedicine.org/, Annals of Internal Medicine at http://www.annals.org/, and Epidemiology at http://www.epidem.com/). Information on the STROBE Initiative is available at www.strobe-statement.org.

For peer review only

BMJ Open

MATERNAL ANEMIA AND UNDERWEIGHT AS DETERMINANTS OF PREGNANCY OUTCOMES: COHORT STUDY IN EASTERN RURAL MAHARASHTRA, INDIA

Journal:	*BMJ Open*
Manuscript ID	bmjopen-2018-021623.R2
Article Type:	Research
Date Submitted by the Author:	12-May-2018
Complete List of Authors:	Patel, Archana; Lata Medical Research Foundation, Nagpur<br>Prakash, Amber; Lata Medical Research Foundation, Nagpur, Research Unit<br>Das, Prabir Kumar; Lata Medical Research Foundation, Nagpur<br>Gupta, Swarnim ; Lata Medical Research Foundation, Nagpur<br>Pusdekar, Yamini; Lata Medical Research Foundation, Nagpur<br>Hibberd, Patricia; Boston University School of Public Health; Boston University School of Medicine
**Primary Subject Heading**:	Public health
Secondary Subject Heading:	Global health, Epidemiology, Nutrition and metabolism
Keywords:	Body Mass Index, Anemia, Pregnancy, Neonatal Deaths, Malnutrition, Stillbirth

SCHOLARONE™
Manuscripts

MATERNAL ANEMIA AND UNDERWEIGHT AS DETERMINANTS OF PREGNANCY OUTCOMES: COHORT STUDY IN EASTERN RURAL MAHARASHTRA, INDIA

Archana Patel[1], Amber Abhijeet Prakash*[1], Prabir Kumar Das[1], Swarnim Gupta[1], Yamini Pusdekar[1], Patricia L Hibberd[2]

1. Lata Medical Research Foundation, Nagpur, India
2. Boston University School of Public Health and Boston University School of Medicine, Boston, United Stated of America

First Author:

Archana Patel(MD PhD)

Program Director

Address: Lata Medical Research Foundation,

Vasant Nagar, Nagpur- 440022,

Maharashtra, India

Telephone: +0712-2249569

Email: dr_apatel@yahoo.com

*Second and Corresponding Author:

Amber Abhijeet Prakash(MSc)

Senior Statistician and Data Manager

Address: Lata Medical Research Foundation,

Vasant Nagar, Nagpur- 440022,

For peer review only

Maharashtra, India

Telephone: +0712-2249569

Email: rockman.blues21@gmail.com

Third Author:

Prabir Kumar Das(MD)

Country Coordinator

Address: Lata Medical Research Foundation,

Vasant Nagar, Nagpur- 440022,

Maharashtra, India

Telephone: +0712-2249569

Email: prabir_das23@rediffmail.com

Fourth Author:

Swarnim Gupta (PhD)

Nutrition Consultant

Address: Lata Medical Research Foundation,

Vasant Nagar, Nagpur- 440022,

Maharashtra, India

Telephone: +0712-2249569

Email: gupta.swarnim@gmail.com

Fifth Author:

For peer review only

For peer review only - http://bmjopen.bmj.com/site/about/guidelines.xhtml

Yamini Pusdekar(MD)

Research Co-ordinator

Address: Lata Medical Research Foundation,

Vasant Nagar, Nagpur- 440022,

Maharashtra, India

Telephone: +0712-2249569

Email: dryaminipusdekar27@gmail.com

Sixth Author:

Patricia L Hibberd(MD PhD)

Chair, Department of Global Health

Professor of Global Health, Boston University School of Public Health

Professor of Medicine, Boston University School of Medicine

372 Crosstown Building

801 Massachusetts Avenue

Boston, MA 02118

Email:plh0@bu.edu

Telephone:617-414-1260

WORD COUNT: 6,171

For peer review only - http://bmjopen.bmj.com/site/about/guidelines.xhtml

COPYRIGHT

The Corresponding Author has the right to grant on behalf of all authors and does grant on behalf of all authors, an exclusive license (or non exclusive for government employees) on a worldwide basis to the BMJ Publishing Group Ltd to permit this article (if accepted) to be published in BMJ editions and any other BMJPGL products and sublicenses such use and exploit all subsidiary rights, as set out in our license

For peer review only

For peer review only - http://bmjopen.bmj.com/site/about/guidelines.xhtml

ABSTRACT

Objectives: To study the trend in the prevalence of anemia and low BMI pregnant women from Eastern Maharashtra and evaluate if low BMI and anemia affect pregnancy outcomes.

Design: Prospective observational cohort study.

Setting: Catchment areas of 20 rural primary health centers in 4 eastern districts of Maharashtra State, India.

Participants: 72,750 women from the Nagpur site of Maternal and Newborn Health Registry of NIH's Global Network, enrolled from 2009 to 2016.

Main outcome measures: Mode of delivery, pregnancy related complications at delivery, stillbirths, neonatal deaths and low birth weight (LBW) in babies.

Results: Over 90% of the women included in the study were anemic and over a third were underweight (BMI<18 kg/m2) and with both conditions. Mild anemia at any time during delivery significantly increased the risk (Risk ratio; 95% confidence interval (RR;(95% CI)) of stillbirth (1.3(1.1 - 1.6)), neonatal deaths (1.3(1 - 1.6)) and LBW babies (1.1(1- 1.2)) . The risks became even more significant and increased further with moderate/severe anemia any time during pregnancy for stillbirth (1.4(1.2 - 1.8)), neonatal deaths (1.7(1.3 - 2.1)) and LBW babies (1.3(1.2 - 1.4)).,. Underweight at anytime during pregnancy increased the risk of neonatal

For peer review only - http://bmjopen.bmj.com/site/about/guidelines.xhtml

deaths (1.1(1 - 1.3)) and LBW babies (1.2;(1.2 - 1.3)).The risk of having stillbirths (1.5;(1.2 - 1.8)), neonatal deaths (1.7;(1.3 - 2.3)) and LBW babies (1.5;(1.4 - 1.6)) was highest when - the anemia and underweight co-existed in the included women. Obesity/overweight during pregnancy increased the risk of maternal complications at delivery (1.6;(1.5 - 1.7)) and of cesarean section (1.5;(1.4 - 1.6)) and reduced the risk of LBW babies 0.8(0.8 - 0.9)).

Conclusions: Maternal anemia is associated with enhanced risk of stillbirth, neonatal deaths and LBW. The risks increased if anemia and underweight were present simultaneously.

Trial registration: Registered at ClinicTrials.gov (NCT01073475)

Keywords: Body Mass Index, anemia, pregnancy, neonatal deaths, malnutrition, stillbirth, low birth weight

For peer review only

STRENGTHS AND LIMITATIONS

1. This is a large prospective study with a sample size of 72,750 pregnant women collected over an 8year period in the target geographical region of Eastern Maharashtra, focusing on the synergistic effects of low body mass index (BMI) and anemia anytime in pregnant women, on stillbirths, neonatal death and low birth weight in newborns, which may have been overlooked in prior research.
2. Dietary practices and details of compliance with iron supplementation data was not collected in this study.
3. Pre-pregnancy data on BMI and anemia were not available in this study. The broad of range of gestational age during first visit mitigates the exclusive pathophysiologic effect of undernutrition in women with low BMI due to under-nutrition,on pregnancy outcomes.
4. The hemoglobin levels were measured using Sahli's method, which may not be accurate due to subjectivity of the method, poor sensitivity and lack of reliability.

For peer review only

INTRODUCTION

Malnutrition is a serious underlying cause for child and maternal deaths around the globe. Undernutrition during pregnancy restricts fetal growth, contributing to about 800,000 neonatal and 400,000 infant deaths, and 20% of stunting in the first 2 years of the child's life, as well as 20% of maternal deaths at delivery. [1-3]The Sustainable Development Goals (SDG) bring explicit attention to nutrition, including the World Health Assembly target to reduce anemia in women of ages 15-49 years by 50% by 2025.[4-5] Since India has largest number of neonatal, infant and under age 5 children deaths in the world as well as high rates of stunting and growth faltering,[6-8], it is particularly timely to understand maternal nutritional status as a risk factor for maternal and childhood adverse outcomes of pregnancy in the Indian population.

Two important indicators of maternal nutrition are Body Mass Index (BMI) and anemia, both of which can affect health of a mother and her fetus. Much attention has been focused on complications of maternal overweight and obesity (BMI>25 kg/m^2) including gestational hypertension, pre-eclampsia, macrosomia, early induction of labor and need for caesarian deliveries and currently apply mostly to high but also recognized increasingly in middle-income countries, including India.[9]However, in rural India, undernutrition (BMI<18.5 kg/m^2) predominates and is associated with low birth weight (LBW, <2.5kg) and preterm deliveries (<37 weeks of gestation). Pre-pregnancy BMI is most indicative of maternal undernutrition but this data is rarely available when a woman enrolls in the ante-natal clinics in many developing countries including India, where the first ante-natal visit is often after the first trimester. [10] Although the pathophysiology of low BMI early in pregnancy may differ from the same later in

For peer review only - http://bmjopen.bmj.com/site/about/guidelines.xhtml

pregnancy(fetal growth faltering, oligohydramnios etc), there is little information, if any, on the impact of low BMI recorded at the first ante-natal visit regardless of GA, on maternal and fetal outcomes.[11] This information would help to identify high risk pregnant women at their first ante-natal visit. Similarly, maternal anemia, as a result of undernutrition and infections coexist with low BMI, particularly in Indian women of reproductive age. In the 2015 National Family and Health Survey (NFHS), 23% of Indian women had a BMI less than 18.5 kg/m^2 and 53% were anemic (Hemoglobin < 11 gm/dL). [12]Even higher rates were reported in rural areas of the state of Maharashtra.[13]Maternal anemia is also associated with post-partum hemorrhage, LBW, small for gestational age (SGA) babies and perinatal death.[14]. However, there is minimal information on the consequences of the combination of low BMI and anemia in pregnant women in rural Maharashtra and whether women with this dual burden should be targeted for specific interventions. We used data from an ongoing Maternal and Newborn Health Registry (MNHR) based in rural areas of Nagpur, Maharashtra, India to (1) describe the prevalence and trends of maternal anemia and low BMI from 2009 to 2016 (2) describe the maternal demographic characteristics associated with different levels of maternal hemoglobin and BMI from 2009-2016, (3) evaluate whether low BMI and anemia were independent or synergistic risk factors for adverse pregnancy outcomes between 2009 and2016, (4) investigate whether low BMI and anemia had additive or synergistic effects on poor birth outcomes.

METHODS

Study design and settings

For peer review only - http://bmjopen.bmj.com/site/about/guidelines.xhtml

The present study utilizes data collected from the Nagpur, India site of MNHR, which is a population-based registry established in 2009 by the Global Network (GN) with support from *Eunice Kennedy Shriver* National Institute of Child Health and Human Development (NICHD), United States of America (USA).The MNHR is an observational, prospective cohort operating in seven different sites around the world including two sites, in India. The Nagpur site comprises of 20 study clusters in the 4 districts of Nagpur, Bhandara, Chandrapur and Wardha in Eastern Maharashtra, India.Geographical areas surrounding government primary health centers (PHC)that have approximately 900 deliveries per year, constitute a study cluster. The MNHR aims to recruit all consenting women residing within the clusters, as early as possible during their pregnancy and then to follow them until 42 days post-partum. The MNHR methods have been published elsewhere. [15] The time period of this study was from June 2009 to December 2016.

Patient and Public Involvement

The research questions for this study, were developed to evaluate frequent reports of tiredness and weakness by the pregnant women enrolled in the prospective MNHR at the Nagpur site in rural Eastern Maharashtra and to further review the reported high prevalence of maternal anemia in the Indian Demographic Health Surveys. Specifically, we wanted to determine whether rates of anemia had decreased over time, as a result of an active national iron supplementation program, and, whether in our prospective cohort, these rates had an impact on pregnancy outcomes. We also wanted to study whether low BMI, common in our study subjects, further exacerbated the impact of anemia in pregnancy on perinatal outcomes. Since we intended to address these questions in a secondary data analysis using de-identified data in the MNHR, patients and public were not involved in the design of this study. The results will not be

For peer review only - http://bmjopen.bmj.com/site/about/guidelines.xhtml

disseminated to study participants but are intended for additional research to find ways to reduce maternal undernutrition (low BMI) and anemia, as well as to inform health policies on pre-pregnancy and maternal nutrition.

Participants

The participants in this study, were all consenting pregnant women residing in the 20 clusters of the Nagpur GN site, enrolled within the study period, who completed data collection(i.e., had delivery outcomes and a completed 42 days post-partum follow-up) at the time of analyses. Women who had medical termination of pregnancy (MTP) (premature expulsion of a non-viable fetus before 20 weeks of gestation), miscarriages, multiple fetuses or had extreme/missing values for one or more study variables of interest were excluded from the analyses. Early enrollment of the participants was ensured through active surveillance in the study clusters by Nagpur site MNHR public health staff. The enrolled women were followed through to a perinatal visit after delivery (within a week) and to a follow up visit around 42 days after birth. Maternal and infant data on study variables and outcomes, were recorded for the enrollees.

Data collection procedures

Briefly, data were collected by trained registry administrators (RA) via standardized forms. RAs included medical officers and auxiliary nurse-midwives (ANM) employed at PHCs and sub-centers. A two-day training for data collection was provided to the Registry Administrators (RAs), followed by a refresher training every six months, and unscheduled special trainings as needed. The RAs returned filled in forms which were manually scrutinized for errors prior to data entry at Lata Medical Research Foundation (LMRF), Nagpur. After entry, data were

For peer review only - http://bmjopen.bmj.com/site/about/guidelines.xhtml

transmitted to the GN data management center (Research Triangle Institute, Durham, North Carolina, USA) where digital data checks were performed on a monthly basis. The data management center forwarded edit, monitoring and performance reports to the Nagpur site team for data cleaning and management. The data were continually reviewed for quality by NICHD appointed data monitoring committee and by the Nagpur site data collection team.

Study variables

At the first antenatal visit, information regarding the enrolled women's, characteristics including age, education, parity, height, weight and hemoglobin was recorded. Weight to nearest 100grams using a spring balance and height to nearest centimeter was measured using a non-flexible measuring tape fixed to a wall. Body mass index (BMI) was calculated by dividing weight in kilograms (kg) by squared height in meters. Standard World Health Organization (WHO) BMI classifications[16] were used to categorize the enrollees as underweight (BMI<18.5 Kg/m^2), normal (BMI 18.5-24.9 Kg/m^2), or as overweight/ obese (BMI≥25Kg/m^2). Hemoglobin was estimated within two weeks of enrollment using Sahli's method.[17] A concentration of hemoglobin (Hb) level < 11 g/dL indicated presence of anemia. Based on WHO classification [18] hemoglobin readings were used to define the severity of anemia as normal (≥11 g/dL), mild (10<11 g/dL) and moderate/severe (<10 g/dL). BMI and anemia were categorized as ordinal variables for assessing their rates in this study population in consistency with standard public health practices. A 4 level combination variable for BMI-anemia was defined as based on anemia -hemoglobin<11(g/dL) (anemic) vs. ≥ 11 (g/dL) (non-anemic) and BMI<18.5 (Kg/m2) (underweight) vs ≥ 18.5 (Kg/m2) (normal or not underweight), creating 'anemic-normal', 'anemic-underweight', 'non anemic-normal' and 'non anemic-underweight'. Mother's age was

For peer review only - http://bmjopen.bmj.com/site/about/guidelines.xhtml

recorded at the time of enrolment. Maternal education of up to 4 grades was considered 'primary', grade 5 to grade 10 as 'secondary' and more than grade 10 as 'university'. Parity was recorded as whole numbers zero or more, depending on if the woman had been pregnant before enrollment or in pregnancy had carried a fetus to 20 weeks of gestation, or not.

Study Outcomes

The impact of 3 level variables of anemia (normal, mild, moderate/severe) and BMI (underweight, normal, overweight/obese) was assessed on rates of mode of delivery, any pregnancy related complications at delivery, and, post-delivery birth outcomes. While we did not assess the determinants of maternal mortality as maternal mortality rates were low, we described the causes of death with respect to the level of anemia and BMI status. Mode of delivery was recorded as cesarean section(CS) or non-cesarean (vaginal, assisted vaginal).Any pregnancy related complications at delivery were recorded as presence of one or more of the following; obstructed/prolonged labor, severe antepartum hemorrhage (APH), severe postpartum hemorrhage (PPH), hypertension, pre-eclampsia, and oblique/abnormal lie. Birth outcomes such as stillbirth, neonatal deaths (deaths of neonates from birth to 28 days of life) and low birth weight (LBW) rates were assessed. Stillbirth rates were calculated as number of stillbirths per 1000 total births while neonatal death rates were calculated as number of neonatal deaths per 1000 live births. Birth weight was measured within 24 hours of birth by using either a pan spring or a pan digital weighing scale. LBW was defined as neonatal birth weight less than 2500 grams.

For peer review only

For peer review only - http://bmjopen.bmj.com/site/about/guidelines.xhtml

Statistical Analyses

Data were analyzed using the STATA 13.1 statistical package (Stata Corp, College Station, Texas, USA). Study variables of interest and their combinations, with years of enrollment were cross tabulated in Stata and were transformed to graphs either in Stata itself or in Microsoft Excel. Pearson's correlation coefficient was used to study correlation between hemoglobin concentrations and BMI values and was tested for significance. A univariate multinomial logistic regression, adjusting for clustering was used to identify association between the WHO levels of anemia and BMI categories. Significance of trends over the progression of years of enrollment in any of the study variables was determined using Cuzick's non-parametric test [19] for trend across ordered groups. The year-wise rates of the outcomes (CS, pregnancy related complication and LBW) were determined for each level of anemia and category of BMI. Stillbirths and neonatal death rates were calculated per 1000 total births and per 1000 live births respectively. To assess the association of maternal demographic characteristics, anemia and BMI with the study outcomes the data was divided in two data sets, one which included participants who had their first ante-natal visit ≤ 20 weeks of GA and the second set that included participants who had their first ante-natal visit after 20 weeks of GA. Both data sets were analyzed using multivariable regression analyses solved with generalized estimating equations (GEE), adjusting for clustering within the PHCs. For the first set of regression models, explanatory variables included demographic characteristics - mother's age, education level and parity, along with the 3 levels of anemia and 3 categories of BMI, as independent variables while dependent variables included all 5 study outcomes.The regressions were also performed using hemoglobin and BMI as continuous variables and compared with the results when these variables were considered as ordinal variables. The interaction of hemoglobin and BMI was also assessed. A second set of

For peer review only

model used demographic characteristics along with the constructed anemia-BMI variable('anemic-normal', 'anemic-underweight', 'non anemic-normal' and 'non anemic-underweight') as explanatory variables for 3 post-natal outcomes–stillbirths, neonatal death and LBW. These models assessed the impact of anemia or under-weight either alone or in combination on the 3 post-natal outcomes, as it is relevant from the public health viewpoint to understand the risks of adverse outcomes when a combination of co-morbidities such as anemia and underweight co-exist.

RESULTS

Participants

During the study period, 72,750 women had delivery outcomes and complete follow up through day 42 post-partum was completed. Women with invalid or missing data on BMI or hemoglobin (N=764) and miscarriage/MTP/multiple gestation (N=4412) were excluded.

Baseline maternal characteristics

Almost 91% of the included women were anemic (65811/72750), over a third were underweight (25571/72750, 35.1%), and over a third were both anemic and underweight(23867/72750, 32.8%). Anemia was severe in less than 0.2% and moderate in nearly 48% women. The gestational age at the first antenatal visit was ≤20 weeks for 72%, 21-28 weeks for 15% and > 28 weeks for 13% of the study population. The study population mostly aged 20-29 years (93%); about half were primiparous 48% vs. multiparous, 52%. As shown in Table 1, over time there

For peer review only - http://bmjopen.bmj.com/site/about/guidelines.xhtml

was an increase in pregnant women in the older age groups>29 over and those who completed higher levels of education in later years (2015-2016). For parity, there was a reduction in proportions of parity>2 in 2016. The levels of anemia showed no significant changes over time. The proportion of women who were just underweight (28% to 38%) (Figure 1), and, underweight with anemia (25% to 35%) (Figure 2) increased over time. We found a highly significant correlation(r= 0.2; P<0.001) between hemoglobin and BMI. Multinomial logistic regression with normal hemoglobin concentrations as reference, showed highly significant (P<0.001) associations between women who were underweight and those with moderate/severe anemia (Risk Ratio (RR):1.9, 95% Confidence Interval (CI):1.6-2.2) as well as those with mild anemia (RR:1.3, 95% CI:1.2-1.5).

For peer review only - http://bmjopen.bmj.com/site/about/guidelines.xhtml

Table 1: Baseline Characteristics of women in the study, by year of enrollment

Characteristics	Years								
	**2009**<br>N (%)<br>6433 (100)	**2010**<br>N (%)<br>10447 (100)	**2011**<br>N (%)<br>9626 (100)	**2012**<br>N (%)<br>9905 (100)	**2013**<br>N (%)<br>9496 (100)	**2014**<br>N (%)<br>9454 (100)	**2015**<br>N (%)<br>10108 (100)	**2016**<br>N (%)<br>7281 (100)	**Total**<br>N (%)<br>72750 (100)
Mother's Age (years)*									
<20	131 (2)	209 (2)	181 (1.9)	188 (1.9)	190 (2)	193 (2)	209 (2.1)	157 (2.2)	1458 (2)
20-29	6044 (94)	9867 (94.4)	9056 (94.1)	9269 (93.6)	8834 (93)	8743 (92.5)	9266 (91.7)	6640 (91.2)	67719 (93.1)
>29	258 (4)	371 (3.6)	389 (4)	448 (4.5)	472 (5)	518 (5.5)	633 (6.3)	484 (6.6)	3573 (4.9)
Mother's Education (grades)*									
Primary or Less (<=4)	1476 (22.9)	2281 (21.8)	1846 (19.2)	1927 (19.5)	1882 (19.8)	693 (7.3)	633 (6.3)	329 (4.5)	11067 (15.2)
Secondary(5-10)	3814 (59.3)	6210 (59.4)	5818 (60.4)	5880 (59.4)	5432 (57.2)	4962 (52.5)	5101 (50.5)	3522 (48.4)	40739 (56)
University(>10)	1139 (17.7)	1956 (18.7)	1958 (20.3)	2071 (20.9)	2172 (22.9)	3768 (39.9)	4358 (43.1)	3415 (46.9)	20837 (28.6)
Missing	4 (0.1)	0 (0)	4 (0)	27 (0.3)	10 (0.1)	31 (0.3)	16 (0.2)	15 (0.2)	107 (0.1)
Parity*									
Nulliparous	2905 (45.2)	5118 (49)	4706 (48.9)	4817 (48.6)	4384 (46.2)	4530 (47.9)	4967 (49.1)	3598 (49.4)	35025 (48.1)
1-2	3300 (51.3)	5032 (48.2)	4669 (48.5)	4827 (48.7)	4907 (51.7)	4721 (49.9)	4888 (48.4)	3544 (48.7)	35888 (49.3)
>2	228 (3.5)	297 (2.8)	249 (2.6)	261 (2.6)	204 (2.1)	197 (2.1)	234 (2.3)	137 (1.9)	1807 (2.5)
Missing	0 (0)	0 (0)	2 (0)	0 (0)	1 (0)	6 (0.1)	19 (0.2)	2 (0)	30 (0)
Anemia (gm/dl)									
Moderate/Severe(<10)	2962 (46)	5283 (50.6)	4620 (48)	4730 (47.8)	4682 (49.3)	4418 (46.7)	5058 (50)	3457 (47.5)	35210 (48.4)
Mild(10<11)	2743 (42.6)	4341 (41.6)	4033 (41.9)	4337 (43.8)	4006 (42.2)	4152 (43.9)	4041 (40)	2948 (40.5)	30601 (42.1)
Normal(>=11)	728 (11.3)	823 (7.9)	973 (10.1)	838 (8.5)	808 (8.5)	884 (9.4)	1009 (10)	876 (12)	6939 (9.5)
Body Mass Index (kg/m^2) *									
Underweight(<18.5)	1784 (27.7)	3537 (33.9)	3391 (35.2)	3450 (34.8)	3411 (35.9)	3424 (36.2)	3786 (37.5)	2788 (38.3)	25571 (35.1)
Normal(18.5-24.9)	4426 (68.8)	6558 (62.8)	5931 (61.6)	6061 (61.2)	5699 (60)	5619 (59.4)	5789 (57.3)	4095 (56.2)	44178 (60.7)
Overweight or Obese(≥25)	223 (3.5)	352 (3.4)	304 (3.2)	394 (4)	386 (4.1)	411 (4.3)	533 (5.3)	398 (5.5)	3001 (4.1)
Anemia(gm/dl)-BMI(kg/m^2) factors									
Anemic(<11)-Normal(>=18.5)	4070 (63.3)	6256 (59.9)	5522 (57.4)	5823 (58.8)	5463 (57.5)	5384 (56.9)	5569 (55.1)	3857 (53)	41944 (57.7)
Anemic(<11)-Underweight(<18.5)	1635 (25.4)	3368 (32.2)	3131 (32.5)	3244 (32.8)	3225 (34)	3186 (33.7)	3530 (34.9)	2548 (35)	23867 (32.8)
Non-Anemic(>=11- Normal(>=18.5)	579 (9)	654 (6.3)	713 (7.4)	632 (6.4)	622 (6.6)	646 (6.8)	753 (7.4)	636 (8.7)	5235 (7.2)
Non-Anemic(>=11)-Underweight(<18.5)	149 (2.3)	169 (1.6)	260 (2.7)	206 (2.1)	186 (2)	238 (2.5)	256 (2.5)	240 (3.3)	1704 (2.3)
Body Mass Index (kg/m^2) [Mean/SD/IQR]	20.1/2.6/3.4	19.7/2.6/3.2	19.6/2.6/3.4	19.7/2.7/3.5	19.7/2.8/3.6	19.7/2.9/3.6	19.7/3/3.7	19.7/3/3.7	19.7/2.8/3.6
Hemoglobin (gm/dl) [Mean/SD/IQR]	9.8/0.9/1	9.7/0.9/1	9.7/0.8/1.1	9.8/0.8/1	9.9/0.8/0.8	9.9/0.8/0.8	9.9/0.8/1	9.9/0.9/1	9.8/0.8/1

* Highly significant (P<0.001) non parametric trend over years of enrollment

For peer review only - http://bmjopen.bmj.com/site/about/guidelines.xhtml

Maternal Mortality

There were 29 maternal deaths in the study population, 20 of whom had severe anemia. Moderate/Severe anemia was identified as a primary cause of death in 1 and as a co-morbid condition in 19. Of the total 29 deaths, anemia and underweight was observed in 8, anemia alone in 19, underweight alone in 1, and overweight/obese in 2. Reasons for maternal deaths, were hemorrhage (n=9, 31%), preeclampsia/eclampsia (n=7, 24%), infection (n=6, 21%), other/missing (n=7, 22%).

Study outcomes by years of enrollment

The rates of stillbirths, neonatal deaths and LBW babies increased with increasing severity of anemia, with lower BMI, and, if anemia and under-weight co-existed as compared to either condition alone, consistently in all years of enrollment. Rates of LBW babies decreased in overweight/obese mothers. The rates of CS and pregnancy related complications increased with increasing BMI. Rates of CS increased over time for all levels of anemia and BMI(Table 2).

For peer review only

Table 2: Rates of study with respect to levels of anemia and body mass index categories, by year of enrolment

	Year of Enrollment								
	**2009**	**2010**	**2011**	**2012**	**2013**	**2014**	**2015**	**2016**	**Total**
**Variables**	**Rates of women experiencing cesarean sections**								
Anemia (gm/dl)									
Moderate/Severe(<10)	15.67	16.83	17.19	19.53	20.91	23.74	24.73	28.67	20.85
Mild(10-11)	16	17.97	19.69	21.51	24.84	26.42	31.08	33.58	23.8
Normal(>=11)	18.41	26.12	24.87	26.13	31.44	32.01	37.07	39.61	29.8
Body Mass Index (kg/m^2)									
Underweight(<18.5)	15.08	15.13	16.13	17.65	19.2	21.61	23.38	26.79	19.5
Normal(18.5-24.9)	15.95	18.65	19.91	21.53	24.64	26.87	29.9	33.41	23.6
Overweight or Obese(≥25)	27.8	35.8	33.55	41.12	43.78	43.55	49.72	53.52	42.59
Anemic(HB< 11 gm/dl) & Underweight (BMI<18.5 kg/m^2)	15.05	14.96	15.94	17.32	18.98	21.19	23.31	26.14	19.22
**Variables**	**Rates of women with pregnancy complications present at delivery**								
Anemia (gm/dl)									
Moderate/Severe(<10)	14.58	14.1	12.03	14.76	14.72	10.5	10.7	10.85	12.78
Mild(10<11)	14.51	14.67	14.95	15.75	17.05	13.97	11.9	12.48	14.49
Normal(>=11)	16.62	21.51	16.65	17.42	22.15	18.1	15.86	14.27	17.73
Body Mass Index (kg/m^2)									
Underweight(<18.5)	13.85	12.58	11.53	14.03	14.07	10.25	10.46	10.26	12.04
Normal(18.5-24.9)	14.91	15.46	14.37	15.38	17.07	13.54	11.99	12.43	14.48
Overweight or Obese(≥25)	19.73	28.41	25.66	28.17	25.39	22.38	17.26	18.34	22.93
Anemic(HB< 11 gm/dl) & Underweight (BMI<18.5 kg/m^2)	13.82	12.44	11.85	14.09	13.64	9.79	10.28	10.09	11.92
**Variables**	**Stillbirth rate per 1000 total births**								
Anemia (gm/dl)									
Moderate/Severe(<10)	29.37	25.55	22.29	23.89	26.27	21.05	23.33	26.61	24.54
Mild(10-11)	27.34	24.65	20.58	19.37	23.71	19.27	18.81	15.6	21.11
Normal(>=11)	12.36	18.23	15.42	16.71	16.09	16.97	14.87	15.98	15.85
Body Mass Index (kg/m^2)									
Underweight(<18.5)	29.15	25.73	22.41	22.32	25.21	20.74	22.45	27.62	24.05
Normal(18.5-24.9)	25.76	24.7	19.9	20.13	24.21	19.93	19.52	17.34	21.5
Overweight or Obese(≥25)	22.42	11.36	23.03	30.46	18.13	12.17	20.64	10.05	18.33
Anemic(HB< 11 gm/dl) & Underweight (BMI<18.5 kg/m^2)	29.97	25.83	23.32	23.43	25.74	21.34	23.23	27.86	24.68
**Variables**	**Neonatal death rate per 1000 live births**								
Anemia (gm/dl)									
Moderate/Severe(<10)	22.06	21.63	19.64	28.74	24.05	21.99	24.05	21.89	23.09
Mild(10<11)	17.54	16.08	15.44	16.74	19.03	18.26	17.72	16.47	17.15
Normal(>=11)	11.25	25.38	11.62	14.8	6.33	12.82	13.25	9.37	13.06
Body Mass Index (kg/m^2)									
Underweight(<18.5)	21.23	20.13	21.26	23.68	24.98	20.09	22.94	20.74	21.96
Normal(18.5-24.9)	18.19	19.28	14.14	21.86	18.13	19.25	19.04	16.42	18.38
Overweight or Obese(≥25)	13.95	20.53	27.68	15.96	13.37	17.54	17.54	18.13	17.97

For peer review only - http://bmjopen.bmj.com/site/about/guidelines.xhtml

Anemic(HB< 11 gm/dl) & Underweight (BMI<18.5 kg/m^2)	21.25	19.26	22.07	24.59	26.48	20.3	22.84	21.06	22.32
**Variables**	**Rates of low birth weight (<2500 g) babies**								
Anemia (gm/dl)									
Moderate/Severe(<10)	17.42	17.87	17.51	18.46	19.14	19.04	20.42	22.65	19.01
Mild(10-11)	16.99	14.37	14.63	13.53	14.5	14.72	16.98	18.69	15.35
Normal(>=11)	14.15	15.43	12.85	11.22	10.4	12.9	16.45	16.32	13.78
Body Mass Index (kg/m^2)									
Underweight(<18.5)	19.79	18.63	17.9	19.59	20.14	19.28	21.61	22.74	19.92
Normal(18.5-24.9)	15.95	15.23	14.69	13.81	14.6	15.09	17.29	19.54	15.61
Overweight or Obese(≥25)	11.66	10.51	15.13	10.41	10.88	14.11	12.38	10.8	11.96
Anemic(HB< 11 gm/dl) & Underweight (BMI<18.5 kg/m^2)	20.18	18.53	17.89	19.79	20.5	19.55	21.5	22.96	20.04

For peer review only

Study Outcomes: Regression

The rates of CS and pregnancy related complications during delivery were significantly higher in non-anemic women vs. anemic women for both data sets. Mild anemia recorded ≤ 20 weeks had no impact on the outcomes, but when recorded after 20 weeks mild anemia increased the risk of stillbirths and neonatal deaths. Moderate/severe anemia increased the risk of neonatal deaths and LBW when recorded ≤ 20 weeks and when present after 20 weeks it also increased the risk of stillbirths. (Table 3A, 3B). The rates of LBW were significantly higher in underweight women anytime during pregnancy (Table 3A, 3B, 3C). The risk of CS as well as pregnancy related complications were higher in women who were overweight/obese recorded anytime during pregnancy while the risk of LBW was reduced in the same group when recorded after 20 weeks of pregnancy (Table 3C). The risks of stillbirth, neonatal deaths and LBW were the highest when women were both anemic and underweight (Table 4).

Nulliparous women had a significantly higher risk for all five outcomes, higher mother's age (>29 years) was associated with stillbirth, neonatal deaths and LBW, while lower maternal education level (secondary or less) was associated with stillbirths, neonatal deaths and LBW. Women < 20 years were at lower risk for CS. Women with lower levels of education were also significantly less likely to have a CS or pregnancy related complication(Table 3C).

For peer review only

Table 3A: Risk factors for study outcomes, multivariable estimates (GA at enrolment ≤ 20 weeks)

	Study Outcomes				
**Characteristics**	**Cesarean Sections**<br><br>**N=11628, 24%**<br>**RR(95% CI)^**	**Pregnancy related Maternal complications**<br>**N=6762, 14%**<br>**RR(95% CI)^**	**Stillbirths**<br><br>**N=1104, 2.3%**<br>**RR(95% CI)^**	**Neonatal deaths (within 28 days of birth)**<br>**N=963, 2.0%**<br>**RR(95% CI)^**	**Low birth weight (<2500 gms)**<br>**N=8378, 18%**<br>**RR(95% CI)^**
Mother's Age (years)					
<20	0.83*(0.73 - 0.95)	0.83*(0.72 - 0.97)	0.73(0.47 - 1.12)	1.23(0.87 - 1.75)	1(0.88 - 1.15)
20-29	ref	ref	Ref	ref	ref
>29	1.56**(1.44 - 1.68)	1.37**(1.22 - 1.53)	1.45*(1.13 - 1.86)	1.37*(1.03 - 1.80)	1.27**(1.15 - 1.40)
Mother's Education (grades)					
Primary or Less (<=4)	0.50**(0.46 - 0.54)	0.76**(0.70 - 0.83)	1.68**(1.39 - 2.04)	1.53**(1.24 - 1.87)	1.17**(1.08 - 1.25)
Secondary(5-10)	0.70**(0.67 - 0.73)	0.90**(0.85 - 0.94)	1.40**(1.21 - 1.61)	1.30**(1.12 - 1.52)	1.15**(1.09 - 1.21)
University(>10)	ref	ref	Ref	ref	ref
Parity					
Nulliparous	1.33**(1.28 - 1.39)	2.00**(1.86 - 2.15)	1.32**(1.17 - 1.50)	1.57**(1.38 - 1.80)	1.38**(1.31 - 1.44)
1-2	ref	ref	Ref	ref	ref
>2	0.49**(0.40 - 0.60)	0.80*(0.63 - 1.00)	1.50*(1.06 - 2.13)	1.62*(1.10 - 2.37)	0.99(0.85 - 1.17)
Anemia (gm/dl)					
Severe/Moderate (<10)	0.89**(0.84 - 0.95)	0.90*(0.83 - 0.98)	1.16(0.92 - 1.46)	1.37*(1.06 - 1.77)	1.24**(1.14 - 1.35)
Mild(10-11)	0.91*(0.86 - 0.97)	0.92*(0.85 - 0.99)	1.12(0.88 - 1.41)	1.16(0.90 - 1.50)	1.09(1.00 - 1.18)
Normal(>=11)	ref	ref	Ref	ref	ref
Body Mass Index (kg/m^2)					
Underweight(<18.5)	0.81**(0.77 - 0.84)	0.83**(0.79 - 0.88)	1.08(0.96 - 1.22)	1.09(0.96 - 1.24)	1.18**(1.13 - 1.23)
Normal(18.5-24.9)	ref	ref	Ref	ref	ref
Overweight or Obese(≥25)	1.64**(1.51 - 1.78)	1.44**(1.28 - 1.61)	1.19(0.84 - 1.67)	1.25(0.87 - 1.79)	0.91(0.79 - 1.04)

* P<0.05, ** P<0.001

^ Obtained by multivariable GEE regression models for each outcome, separately

For peer review only - http://bmjopen.bmj.com/site/about/guidelines.xhtml

Table 3B: Risk factors for study outcomes, multivariable estimates (GA at enrollment>20 weeks)

	Study Outcomes				
**Characteristics**	**Cesarean Sections N= 4816, 20% RR(95% CI)^**	**Pregnancy related Maternal complications N=3260, 14% RR(95% CI)^**	**Stillbirths N=488, 2% RR(95% CI)^**	**Neonatal deaths (within 28 days of birth) N=390, 1% RR(95% CI)^**	**Low birth weight (<2500 gms) N=3776, 16% RR(95% CI)^**
Mother's Age (years)					
<20	0.63*(0.46 - 0.86)	0.78(0.58 - 1.07)	1.28(0.66 - 2.49)	1.47(0.75 - 2.87)	1.17(0.92 - 1.48)
20-29	ref	ref	Ref	ref	ref
>29	1.66**(1.48 - 1.86)	1.37**(1.18 - 1.60)	2.31**(1.70 - 3.13)	1.47(1.00 - 2.16)	1.29**(1.13 - 1.48)
Mother's Education (grades)					
Primary or Less (<=4)	0.45**(0.40 - 0.50)	0.61**(0.54 - 0.68)	1.51*(1.14 - 2.01)	1.56*(1.13 - 2.14)	1.08(0.98 - 1.19)
Secondary(5-10)	0.66**(0.62 - 0.71)	0.87**(0.80 - 0.94)	1.34*(1.05 - 1.71)	1.37*(1.04 - 1.80)	1.10*(1.02 - 1.19)
University(>10)	ref	ref	Ref	ref	ref
Parity					
Nulliparous	1.51**(1.41 - 1.60)	1.98**(1.83 - 2.15)	1.25*(1.04 - 1.51)	1.31*(1.06 - 1.61)	1.31**(1.23 - 1.40)
1-2	ref	ref	Ref	ref	ref
>2	0.42**(0.32 - 0.55)	0.74*(0.56 - 0.98)	0.89(0.56 - 1.42)	1.49(0.95 - 2.34)	1(0.84 - 1.20)
Anemia (gm/dl)					
Severe/Moderate (<10)	0.79**(0.72 - 0.87)	0.87*(0.78 - 0.97)	2.35**(1.56 - 3.53)	2.76**(1.73 - 4.42)	1.33**(1.19 - 1.49)
Mild(10-11)	0.87**(0.80 - 0.94)	0.87*(0.78 - 0.97)	1.81*(1.20 - 2.72)	1.73*(1.08 - 2.76)	1.07(0.96 - 1.20)
Normal(>=11)	ref	ref	Ref	ref	ref
Body Mass Index (kg/m^2)					
Underweight(<18.5)	0.84**(0.77 - 0.91)	0.82**(0.74 - 0.90)	1.12(0.91 - 1.39)	1.12(0.88 - 1.42)	1.28**(1.19 - 1.38)
Normal(18.5-24.9)	ref	ref	Ref	ref	ref
Overweight or Obese(≥25)	1.59**(1.45 - 1.74)	1.55**(1.38 - 1.73)	0.7(0.44 - 1.10)	1.03(0.67 - 1.60)	0.78*(0.68 - 0.91)

* P<0.05, ** P<0.001

^ Obtained by multivariable GEE regression models for each outcome, separately

For peer review only - http://bmjopen.bmj.com/site/about/guidelines.xhtml

Table 3C: Risk factors for study outcomes, multivariable estimates (all Included women)

	Study Outcomes				
**Characteristics**	**Cesarean Sections N=16693, 23% RR(95% CI)^**	**Pregnancy related Maternal complications N=10163, 14% RR(95% CI)^**	**Stillbirths N=1620, 2.2% RR(95% CI)^**	**Neonatal deaths (within 28 days of birth) N=1368, 1.9% RR(95% CI)^**	**Low birth weight (<2500 gms) N=12347, 17% RR(95% CI)^**
Mother's Age (years)					
<20	0.80**(0.72 - 0.90)	0.83*(0.73 - 0.95)	0.88(0.62 - 1.25)	1.28(0.94 - 1.75)	1.04(0.92 - 1.16)
20-29	ref	ref	Ref	ref	ref
>29	1.60**(1.50 - 1.70)	1.38**(1.26 - 1.50)	1.76**(1.45 - 2.12)	1.47**(1.18 - 1.83)	1.28**(1.18 - 1.38)
Mother's Education (grades)					
Primary or Less (<=4)	0.47**(0.44 - 0.50)	0.69**(0.64 - 0.74)	1.62**(1.38 - 1.89)	1.49**(1.26 - 1.77)	1.14**(1.08 - 1.21)
Secondary(5-10)	0.68**(0.66 - 0.70)	0.89**(0.85 - 0.93)	1.38**(1.22 - 1.57)	1.30**(1.14 - 1.49)	1.14**(1.09 - 1.19)
University(>10)	ref	ref	Ref	ref	ref
Parity					
Nulliparous	1.38**(1.34 - 1.43)	2.00**(1.88 - 2.12)	1.31**(1.18 - 1.45)	1.48**(1.33 - 1.66)	1.35**(1.30 - 1.41)
1-2	ref	ref	Ref	ref	ref
>2	0.45**(0.38 - 0.53)	0.76*(0.64 - 0.90)	1.22(0.93 - 1.62)	1.52*(1.14 - 2.04)	1(0.89 - 1.13)
Anemia (gm/dl)					
Severe/Moderate (<10)	0.87**(0.83 - 0.92)	0.89**(0.84 - 0.95)	1.43**(1.17 - 1.75)	1.67**(1.33 - 2.08)	1.26**(1.18 - 1.35)
Mild(10>11)	0.90**(0.86 - 0.94)	0.90*(0.85 - 0.96)	1.28*(1.05 - 1.57)	1.28*(1.02 - 1.60)	1.08*(1.01 - 1.15)
Normal(>=11)	ref	ref	Ref	ref	ref
Body Mass Index (kg/m^2)					
Underweight(<18.5)	0.85**(0.82 - 0.88)	0.85**(0.81 - 0.88)	1.1(1.00 - 1.22)	1.12*(1.00 - 1.25)	1.21**(1.16 - 1.25)
Normal(18.5-24.9)	ref	ref	Ref	ref	ref
Overweight or Obese(≥25)	1.57**(1.48 - 1.67)	1.48**(1.36 - 1.60)	0.92(0.70 - 1.20)	1.11(0.84 - 1.46)	0.84*(0.76 - 0.94)

* P<0.05, ** P<0.001

^ Obtained by multivariable GEE regression models for each outcome, separately

For peer review only - http://bmjopen.bmj.com/site/about/guidelines.xhtml

For peer review only

Table 4: Anemia-BMI factors for study outcomes, multivariable estimates

	Study Outcomes		
**Characteristics**	**Stillbirths**<br><br>**N=1620, 2.2%**<br>**RR(95% CI)^**	**Neonatal deaths (within 28 days of birth)**<br>**N=1368, 1.9%**<br>**RR(95% CI)^**	**Low birth weight (<2500 gms)**<br>**N=12347, 17%**<br>**RR(95% CI)^**
Mother's Age (years)			
<20	0.88(0.62 - 1.25)	1.28(0.94 - 1.75)	1.04(0.93 - 1.16)
20-29	ref	Ref	ref
>29	1.75**(1.45 - 2.11)	1.48**(1.19 - 1.84)	1.27**(1.17 - 1.37)
Mother's Education (grades)			
Primary or Less (<=4)	1.64**(1.40 - 1.91)	1.52**(1.29 - 1.80)	1.16**(1.10 - 1.23)
Secondary(5-10)	1.40**(1.23 - 1.58)	1.32**(1.16 - 1.51)	1.15**(1.10 - 1.20)
University(>10)	ref	Ref	ref
Parity			
Nulliparous	1.30**(1.18 - 1.44)	1.47**(1.31 - 1.64)	1.35**(1.29 - 1.40)
1-2	ref	Ref	ref
>2	1.24(0.94 - 1.63)	1.56*(1.17 - 2.09)	1.02(0.91 - 1.15)
Anemia(gm/dl)-BMI(kg/m^2) factors			
Anemic(<11)-Normal(>=18.5)	1.30*(1.04 - 1.63)	1.54**(1.19 - 2.00)	1.22**(1.13 - 1.32)
Anemic(<11)-Underweight(<18.5)	1.47**(1.17 - 1.85)	1.74**(1.33 - 2.26)	1.49**(1.37 - 1.62)
Non-Anemic(>=11- Normal(>=18.5)	ref	Ref	ref
Non-Anemic(>=11)-Underweight(<18.5)	0.96(0.62 - 1.48)	1.37(0.88 - 2.13)	1.37**(1.20 - 1.57)

* P<0.05, ** P<0.001

^ Obtained by univariate GEE regression models for each outcome, separately

For peer review only - http://bmjopen.bmj.com/site/about/guidelines.xhtml

DISCUSSION

Maternal malnutrition continues to be a silent emergency particularly in low middle-income countries (LMICs) like India. We attempted to understand through present study, not only the trends in the prevalence of anemia and low BMI over the eight years but the less understood combination of low maternal BMI and anemia with pregnancy related outcomes among rural women from Eastern Maharashtra. We found that more than 90% of the pregnant women were anemic, 35% were underweight (BMI< 18.5) and nearly a third had both conditions. It was alarming that the rates of anemia remained unchanged over eight years (2009-2016) and those of underweight increased. The majority of women had mild to moderate anemia, with severe anemia in less than 0.2%. While mild anemia recorded before 20 weeks of GA did not increase the risk of stillbirths, neonatal deaths or LBW, it increased the risk of stillbirths and neonatal deaths when recorded later in the pregnancy (>20 weeks). Moderate/severe anemia recorded anytime during pregnancy increased the risk of neonatal deaths and LBW. It also increased the risk of stillbirths when recorded later in pregnancy. In developing countries, women often enroll late in the antenatal care services. In our study population, approximately a third enrolled after 20 weeks of pregnancy, 90% of which were anemic and nearly half had moderate/severe anemia. This study shows that one public health action of early and aggressive management of anemia could lower the risk of adverse neonatal outcomes. Rates of underweight increased by 10% over eight years, and there was an increase in the rates of overweight/obese from 2012 to 2016. Under-weight recorded any time during pregnancy increased the risk of LBW (Table 3A, 3B). A third of this study population was underweight any time during pregnancy, which is an underestimation of pre-pregnancy BMI. More than a third of women were both anemic and underweight. The risk of stillbirths, neonatal deaths and LBW increased further if both anemia

For peer review only - http://bmjopen.bmj.com/site/about/guidelines.xhtml

and underweight co-existed (Table 4). A limiting factor in our study was that we were not able to evaluate likely confounders of adverse outcomes in pregnancy such as socio-economic status, number and timing of ante-natal visits and use of tobacco, etc. as these data were not collected in the MNH registry.

The NFHS data (NFHS-3 and NFHS-4) found anemia prevalence rate of 54% between 2005 and 2016, with a minimal reduction in Maharashtra between NFHS-3(58%) to NFHS-4 (49%).[12, 17, 18] Disturbingly, our estimates were not only higher than the District Level Household Survey (DLHS-4, 2012-2013) which reported a rate between 43% and 49% among pregnant women aged 15-49 years in the Nagpur, Bhandara and Wardha districts, but also remained static over the past eight years [19] Previous studies from eastern Maharashtra have reported similar high rates of anemia. [20-23] The women in our study were young (20-29years), from both rural and backward tribal regions known for high prevalence of micronutrient deficiency specifically iron deficiency anemia and other causes of anemia such as sickle cell anemia, hookworm infestation and malaria.[24-29]Variations in rates of anemia may be due to methods used for surveying and assessing hemoglobin levels.[30-31] The unchanging status of anemia could be as a result of irregular and poor coverage of the national iron supplementation program [32-34] and suboptimal compliance with iron supplementation during pregnancy (<30%), [10,35] due to side effects, forgetfulness, and false beliefs about the importance of anemia in pregnancy.[36-38] Directly observed administration of iron supplementation may improve compliance and is being implemented for adolescent girls in India.[39,40] We believe it should be considered in pregnant women. Some reduction in rates of severe anemia were observed which may be attributable to improved attendance of ante-natal care visits, early detection and current management guidelines

For peer review only - http://bmjopen.bmj.com/site/about/guidelines.xhtml

using injectable iron for treatment of severe anemia at the public health facilities from the 12th Five year plan.[41]

Our study reports over a third of the study participants to be underweight during pregnancy, which is consistent with the findings of NFHS for rural Nagpur.[12]We also found an 8% increase in low BMI in pregnant women between 2009 and 2016. There was also a trend towards BMI from the normal to overweight/obese categories over this same time period as has been observed.[42]

Anemia and underweight co-existed among one-third of the study participants which increased over time. This change may be due to increasing prevalence of underweight as the rates of anemia did not change over the study period. The most probable cause for maternal underweight in this region would be malnutrition.[43,44] It is also likely that iron and other micronutrient deficiency were responsible for higher rates of anemia in underweight women[45-47] but this should be studied further to reduce rates of anemia in the community.

The rates of CS increased over the years consistent with an increase in facility based deliveries in India.[48] However, anemia at any level reduced the likelihood of CS and pregnancy related complication and underweight and anemic women are more likely to have fetal growth retardation and smaller babies.[49-52] Pregnancy related complication in our study population are most commonly due to obstructed labor and toxemia of pregnancy, both of which are less likely to occur in underweight and anemic women who tend to have smaller babies.[47] Women with higher BMI (who are less likely to be anemic) are at increased risk of miscarriage, gestational diabetes, preeclampsia, venous thromboembolism, induced labor, caesarean section, anesthetic complications and wound infections.[48]

For peer review only - http://bmjopen.bmj.com/site/about/guidelines.xhtml

The risk of LBW was mostly determined by low BMI and severity of anemia any time during pregnancy. Therates of LBW increased over the years in women who had moderate/severe anemia and in women who were underweight. Other Indian studies have reported similar results [11]. The risk of having LBW babies in women who were underweight also increased by 20%, consistent with other studies that have reported an association of maternal anthropometry and size of the baby.[2,3] A study in Tanzania, on 12,269 newborns, identified short maternal stature as an significant risk factor for SGA and preterm-appropriate for gestational age.[53] A recent study from Mexico reported that higher proportions of SGA neonates were born to malnourished and anemic mothers than the mothers who were either malnourished or anemic.[54]LBW babies are at increased risk of becoming stunted adults whichwill expound the inter-generational burden of LBW. [55]

The risk of stillbirths increased when any anemia was recorded after 20 weeks of GA. While there are studies to support our findings on the association of maternal anemia on stillbirths,[11,56,57] it is unclear whether it was as a result of an intrapartum complication or whether it reflected poor socio-economic conditions that limited access to quality intra-partum care. It is also possible thatsickle cell anemia, hookworm infestation and malaria, all common in Eastern Maharashtra may have contributed to higher rates of stillbirths, [20-25]as does ethnicity in underweight women previously.[58]

The neonatal deaths increased when mild anemia was recorded after 20 weeks of GA and if moderate/severe anemia was recorded at any time during pregnancy. Although there is a paucity

For peer review only - http://bmjopen.bmj.com/site/about/guidelines.xhtml

of data on the reason for an association between anemia and neonatal mortality, it is consistent with the finding of a recent meta-analysis in LMIC (one study from India and another from Malawi)that found the odds of neonatal mortality to be 2.7 times among the anemic mothers. [55, 59, 60] The neonatal mortality could also be attributed to increased risk of preterm and LBW deliveries in women who are anemic [61] Underweight recorded any time during pregnancy did not increase neonatal death. However, when the two conditions co-existed, the risk was higher than presence of either one of them alone (table 4).Low maternal BMI and anemia are associated with LBW and preterm deliveries which are independent risk factors for infant mortality in India[62,63].

CONCLUSIONS AND PUBLIC HEALTH IMPLICATIONS

The combination of anemia and underweight in pregnancy increased the risk of stillbirths, neonatal deaths and delivering LBW babies. These findings raise major concerns because national programs to address iron deficiency anemia have not reduced the rates of anemia among the rural pregnant women in eastern Maharashtra over past 8 years. There has been a steady increase in prevalence of underweight in pregnancy with almost a third of pregnant women being both anemic and underweight. Immediate and effective public health interventions are needed to address maternal anemia and malnutrition to improve birth outcomes. There is an urgent need for evaluation and addressing of gaps of the iron supplementation program for adolescent girls and pregnant women in these areas with high rates of anemia, in addition to a more holistic approach to curb maternal malnutrition. Meanwhile, seeking simultaneously short-term strategies such as

For peer review only - http://bmjopen.bmj.com/site/about/guidelines.xhtml

referral to a better healthcare service and management of delivery for the segment of pregnant population who are both anemic and underweight could be helpful in reducing neonatal mortality. Such actions are imperative to break the intergenerational cycle of poor growth in the offspring and also for improving child survival.

LIST OF ABBREVIATIONS

ANM - Auxiliary Nurse Midwife

APH - Antepartum Hemorrhage

BMI - Body Mass Index

CI - Confidence Interval

CS - Cesarean Section

DLHS - District Level Household Survey

GEE - Generalized Estimating Equations

GN - Global Network

IFA - Iron Folic Acid

IRB - Institutional Review Board

LBW - Low Birth Weight

LMIC - Low and Middle-Income Countries

LMRF - Lata Medical Research Foundation

MNHR - Maternal and Newborn Health Registry

MTP - Medical Termination of Pregnancy

For peer review only - http://bmjopen.bmj.com/site/about/guidelines.xhtml

NFHS - National Family and Health Survey

NICHD - *Eunice Kennedy Shriver* National Institute of Child Health and Human Development

PHC –Primaryhealth Center

PPH - Postpartum Hemorrhage

RA - Registry Administrator

RR - Risk Ratio

SGA - Small for Gestational Age

USA - United States of America

WHO - World Health Organization

AUTHOR CONTRIBUTIONS

AP conceived and designed the study, and developed the initial data collection tools; AP and AAP wrote the first draft of the manuscript, which PLH, PKD, YVP and SG subsequently revised. AP and AAP developed the analysis plan outline and AAP carried out the data analyses. AP, PLH, PKD, YVP and SG contributed to data analysis through review, interpretation and cross-checking figures and tables. All authors read, revised, and approved the final manuscript.

ACKNOWLEDGEMENTS

For peer review only - http://bmjopen.bmj.com/site/about/guidelines.xhtml

We are deeply indebted to Research Triangle International, Durham, USA for their enthusiastic support. Thanks and recognition are due to the Registry Administrators at the Nagpur site of the MNHR who collect data, often in very challenging circumstances, and the communities, families, and mothers who agree to participate in the MNHR. We express our sincere gratitude to Dr. VaishaliKhedikar, Dr. SavitaBhargav and Dr, KunalKurhe at LMRF, who helped immensely by strengthening the manuscript through their inputs.

DATA SHARING

A de-identified minimal dataset that underlies the findings and conclusions described in the manuscript can be shared upon request by the editors to verify the reported study findings.All authors hereby declare that individual participant data in any form is not a part of this publication.

FUNDING

This study was funded by the *Eunice Kennedy Shriver* National Institute of Child Health and Human Development of the US National Institutes of Health funded this study (Grants Reference Nos. U01HD058322 and U01HD078439).

For peer review only - http://bmjopen.bmj.com/site/about/guidelines.xhtml

ETHICS

The study in Nagpur, India has been reviewed and approved by the Institutional Review Board (IRB) of theLata Medical Research Foundation (RPC#22A)and the Partners Human Research Committee/IRB in Boston, MA (H-35430).

CONSENT TO PARTICIPATE

The study wasexplained thoroughly and in detail, to the women who lived in the participating communities. The women were enrolled in the study only after giving informed consent. An IRB approved consent form was used for this purpose.

COMPETING INTERESTS

None declared

TRANSPARENCY

For peer review only

The lead author(ABP) affirms that the manuscript is an honest, accurate, and transparent account of the study being reported; that no important aspects of the study have been omitted; and that any discrepancies from the study as planned have been explained.

For peer review only

For peer review only - http://bmjopen.bmj.com/site/about/guidelines.xhtml

1
2
3
4

What is already known on this topic

Low body mass Index (BMI) and anemia during pregnancy have been independently identified as risk factors for delivering pre-term, small for gestational age (SGA) and LBWs, however their relationship with respect to neonatal mortality and stillbirth remains inconclusive. Also, their relationship of severity of anemia with adverse pregnancy outcomes is not well established for low middle-income countries (LMIC). The risk arising out of co-existence of anemia and low BMI -a common scenario in LMIC like India, on poor maternal has not been characterized.

What this study adds

This study showed a high prevalence of anemia (>90%) and underweight (35%) among rural pregnant women in eastern Maharashtra. The rate of anemia remained unaltered but there was an increase in the underweight and overweight/obese woman over the past 8 years from 2009-2016. This highlights the magnitude of persistent public health problem of nutrition origin despite the national programs in place and also the emerging dual burden of diseases in the rural areas as well.

Anemia and underweight during pregnancy were individually found to increase the risk of stillbirths, neonatal deaths and LBW. Women with moderate-severe anemia were at greater risk as compared to the mildly anemic women. These risks increasedfurther if anemia and underweight were present together during pregnancy. Thisraisesconcerns as the two conditions often co-exist in LMICs and in the present study was, as high as, affecting a third of the study population.

Recommendations for policy

There is urgent need to address maternal malnutrition. Though holistic approach would be eradicating maternal malnutrition through more targeted and intensive food or medicinal supplement based approach, seeking short term strategies such as referral to a better healthcare service simultaneously could help in breaking the intergenerational cycle of poor growth in the offspring and reducing child mortality.

For peer review only - http://bmjopen.bmj.com/site/about/guidelines.xhtml

REFERENCES:

1. Black RE, Victora CG, Walker SP, et al. Maternal and child undernutrition and overweight in low-income and middle-income countries.*Lancet*2013; 382(9890): 427-51. doi: 10.1016/S0140-6736(13)60937-X. (accessed 30 November, 2017).
2. Bhutta ZA, Das JK, Rizvi A, et al. Evidence-based interventions for improvement of maternal and child nutrition: what can be done and at what cost? *Lancet* 2013; 382(9890): 452-77. doi:10.1016/S0140-6736(13)60996-4.(accessed 30 November, 2017).
3. Black RE, Allen LH, Bhutta ZA, et al. Maternal and child undernutrition: global and regional exposures and health consequences. *Lancet* 2008; 371(9608):243-60. doi: 10.1016/S0140-6736(07)61690-0.(accessed30 November, 2017).
4. United Nations Development Program.*Sustainable Development Goals*. http://www.undp.org/content/undp/en/home/sustainable-development-goals.html (accessed 30 November 2017).
5. World Health Organization. *Global nutrition targets 2025: policy brief series (WHO/NMH/NHD/14.2).2014*.http://apps.who.int/iris/bitstream/10665/149018/1/WHO_NMH_NHD_14.2_eng.pdf?ua=1(accessed on 11 Sept 2017).
6. Government of India. *Millennium Development Goals: India Country Report 2015*. http://mospi.nic.in/sites/default/files/publication_reports/mdg_2july15_1.pdf(accessed on 11 Sept 2017).
7. United Nations Inter-agency Group for Child Mortality. *Levels & Trends in Child Mortality: Report 2015*. https://www.unicef.org/publications/files/Child_Mortality_Report_2015_Web_9_Sept_15.pdf(accessed on 30 Nov 2017).
8. United Nations Children's Fund. *Committing to Child Survival: A promise renewed. Progress Report 2015*. http://www.apromiserenewed.org/wp-content/uploads/2015/09/APR_2015_8_Sep_15.pdf(accessed on 30 Nov 2017).
9. Van Der Linden EL, Browne JL, Vissers KM, et al. Maternal body mass index and adverse pregnancy outcomes: A Ghanaian cohort study. *Obesity* 2016; 24(1): 215-22. doi:10.1002/oby.21210. (accessed on 30 Nov 2017).

For peer review only - http://bmjopen.bmj.com/site/about/guidelines.xhtml

10. Patel P, Rupani M, Patel S. Antenatal care registration and predicting factors of late registration among pregnant women. 2013. Tropical Doctor 43(1):9-12
11. Pawalia A, Yadav V, Kulandaivelan S. Effect of Obesity on Pregnancy Outcomes – Indian Perspective: A Review. 2015. International Journal of Scientific Research Volume: 4(Issue: 7):453-457
12. NairM, ChoudhuryMK, Choudhury SS, et al. Association between maternal anemia and pregnancy outcomes: a cohort study in Assam, India. *BMJ Glob Health* 2016;1:e000026. doi:10.1136/bmjgh-2015-000026.(accessed30th November 2017).
13. International Institute of Population Sciences. *National Family Health Survey-4: Maharashtra State Fact Sheet*. http://rchiips.org/nfhs/pdf/NFHS4/MH_FactSheet.pdf (accessed 30 November 2017).
14. Haider BA, Olofin I, Wang M, et al.Anemia, prenatal iron use, and risk of adverse pregnancy outcomes: systematic review and meta-analysis.*BMJ* 2013;346:f3443. doi:10.1136/bmj.f3443. (accessed30 November 2017).
15. Goudar SS, Carlo WA, McClure EM, et al. The maternal and newborn health registry study of the global network for women's and children's health research. *Int J GynaecolObstet* 2012; 118(3): 190-3. doi:10.1016/j.ijgo.2012.04.022
16. World Health Organization. *Global database on body mass index.* Http://apps.who.int/bmi/index.jsp?introPage=intro_3.html (accessed 30 November 2017)
17. U.S.Agency for International Development/ PATH. *A Manual for Health Workers: Anemia Detection Methods in Low-Resource Settings*;1997. http://pdf.usaid.gov/pdf_docs/PNACC882.pdf(accessed30 November 2017).
18. World Health Organization (WHO*). Preventing and controlling anemia through primary health care: A guide for health administrators and program managers*. Geneva: WHO; 1989. http://www.who.int/nutrition/publications/micronutrients/anaemia_iron_deficiency/9241542497.pdf (accessed 30 November 2017).
19. Cuzick J. A Wilcoxon-type test for trend. *Stat Med* 1985;4(1):87–90.
20. District Level Household survey 4 (2013-14) Available at: http://mospiold.nic.in/national_data_bank/dlhs4src_10feb2016.pdf Accessed on 12th May 2017

For peer review only - http://bmjopen.bmj.com/site/about/guidelines.xhtml

21. Arlappa N, Meshram II, BalakrishnaN,et al. Prevalence of anemia among different physiological groups in the rural areas of Maharashtra. *Indian J Community Health* 2014; 26(3):278-284.

22. International Institute for Population Sciences (IIPS) and Macro International. *National Family Health Survey (NFHS-3), 2005–06, India, Volume I*. Mumbai: IIPS;2007. http://rchiips.org/nfhs/NFHS-3%20Data/VOL-1/India_volume_I_corrected_17oct08.pdf(accessed 1 December 2017).

23. International Institute of Population Sciences(IIPS). *District Level Household and Facility Survey -4. State Fact Sheet Maharashtra (2012-13)*. http://rchiips.org/pdf/dlhs4/report/MH.pdf (accessed 22 November 2017).

24. Colah RB, Mukherjee MB, Martin S, et al. Sickle cell disease in tribal populations in India. *Indian J Med Res*2015;141(5):509-515. doi:10.4103/0971-5916.159492 (accessed 1 December 2017).

25. Corrêa G, Das M, Kovelamudi R,et al. High burden of malaria and anemia among tribal pregnant women in a chronic conflict corridor in India. *Confl and Health 2017*; 11: 10. doi: 10.1186/s13031-017-0113-1 (accessed 1 December 2017).

26. Ahankari AS, Myles PR, Dixit JV, et al. Risk factors for maternal anemia and low birth weight in pregnant women living in rural India: a prospective cohort study. *Public Health* 2017 Oct;151:63-73. doi: 10.1016/j.puhe.2017.06.023(accessed 1 December 2017)

27. World Health Organization. *The prevalence of anemia in women: a tabulation of available information (WHO/MCH/MSM/92)*. 2nd ed. Geneva: World Health Organization; 1992. http://apps.who.int/iris/bitstream/10665/58994/1/WHO_MCH_MSM_92.2.pd (accessed 30 November 2017)

28. Bharati S, Pal M, Som S, Bharati P. Temporal trend of anemia among reproductive-aged women in India. *Asia Pac J Public Health*2015;27(2):NP1193-1207.

29. Balarajan YS, Fawzi WW, Subramanian SV. Changing patterns of social inequalities in anemia among women in India: cross-sectional study using nationally representative data. *BMJOpen* 2013;3(3):e002233 (accessed 1 December 2017).

30. Balasubramaniam P, Malathi A. Comparative study of hemoglobin estimated by Drabkin's and Sahli's methods.*J Postgrad Med* 1992; 38(1):8-9.

For peer review only - http://bmjopen.bmj.com/site/about/guidelines.xhtml

31. Kalaivani K. Prevalence & consequences of anemia in pregnancy. *Indian J Med Res* 2009;130(5):627–33.

32. Pasricha SR, Biggs BA, Prashanth NS, et al. Factors influencing receipt of iron supplementation by young children and their mothers in rural India: local and national cross-sectional studies. *BMC Public Health*2011;11:617. doi: 10.1186/1471-2458-11-617(accessed 1 December 2017).

33. Chaturvedi S, RanadiveB.Are we really making motherhood safe? A study of provision of iron supplements and emergency obstetric care in rural Maharashtra.*Natl Med J India* 2007; 20(6):294-6.

34. Wadgave HV. Burden of anemia among the pregnant women in rural Area. Healthline2011;2:76-7.http://www.iapsmgc.org/index_pdf/53.pdf (accessed 1 December 2017).

35. Pal PP, Sharma S, SarkarTK, et al. Iron and folic acid consumption by the ante-natal mothers in a rural area of India in 2010. *IntJPrev Med* 2013;4(10):1213-1216.

36. Galloway R, Dusch E, Elder L,etal. Women's perceptions of iron deficiency and anemia prevention and control in eight developing countries.*SocSci Med* 2002;55(4):529-44.

37. Mithra P, Unnikrishnan B, Rekha T, et al. Compliance with iron-folic acid (IFA) therapy among pregnant women in an urban area of south India. *Afr Health Sci 2*013;13(4):880-885. 10.4314/ahs.v13i4.3(accessed 1 December 2017).

38. KumarV,Singh P. Insurance coverage under different health schemes in Uttar Pradesh, India. *ClinEpidemiol Glob Health*2017; 5(1): 1-7.http://www.ceghonline.com/article/S2213-3984(16)30011-2/fulltext(accessed 1 December 2017).

39. Kotecha PV, Nirupam S, Karkar PD. Adolescent girls' anemia control program, Gujarat, India. Indian J Med Res 2009;130(5):584-9.

40. Vir SC, Singh N, Nigam AK, et al. Weekly iron and folic acid supplementation with counseling reduces anemia in adolescent girls: A large-scale effectiveness study in Uttar Pradesh, India. *Food Nutr Bull*2008;29:186-94.

41. Bhatt B, Suryawanshi S, Mali B. "THE NATIONAL HEALTH OUTCOMES IN THE 12th FIVE YEAR PLAN AND THE GOALS THEREOF" International Journal

of Application or Innovation in Engineering & Management (IJAIEM) 2014.l;3(3): 423-25

42. NCD Risk Factor Collaboration (NCD-RisC).Trends in adult body-mass index in 200 countries from 1975 to 2014: a pooled analysis of 1698 population-based measurement studies with 19.2 million participants. Lancet 2016;387(10026):1377-1396. doi: 10.1016/S0140-6736(16)30054-X(accessed 1 December 2017).
43. National Nutrition Monitoring Bureau (NNMB). Diet and nutritional status of rural population, prevalence of hypertension and diabetes among adults and infant and young child feeding practices-A report of third repeat survey. Technical report no. 26.Hyderabad: National Institute of Nutrition, Indian Council of Medical Research; 2012. http://nnmbindia.org/1_NNMB_Third_Repeat_Rural_Survey___Technicl_Report_26.pdf (accessed 1 December 2017).
44. Mahanta LB, Choudhury M, Devi A, et al. On the Study of Pre-Pregnancy Body Mass Index (BMI) and Weight Gain as Indicators of Nutritional Status of Pregnant Women Belonging to Low Socio-Economic Category: A Study from Assam. *Indian J Community Med* 2015;40(3):198-202. doi: 10.4103/0970-0218.158870 (accessed 1 December 2017).
45. Sumarmi S, Puspitasari N, Handajani R, et al. Underweight as a risk factor for iron depletion and iron-deficient erythropoiesis among young women in rural areas of East Java, Indonesia.*Malays J Nutr* 2016;22(2):219–32.
46. Hettiarachchi M, Liyanage C, Wickremasinghe R, et al. Prevalence and severity of micronutrient deficiency: a cross-sectional study among adolescents in Sri Lanka.*Asia Pac J ClinNutr* 2006;15(1):56-63.
47. Darnton-Hill I, Mkparu UC. Micronutrients in Pregnancy in Low- and Middle-Income Countries. *Nutrients* 2015;7(3):1744-1768. doi:10.3390/nu7031744 (accessed on 1 December 2017). https://link.springer.com/article/10.1186/s13690-017-0181-0
48. Patel AB, Prakash AA, Raynes-Greenow C, et al. Description of inter-institutional referrals after admission for labor and delivery: a prospective population based cohort study in rural Maharashtra, India.BMC Health Serv Res2017; 17(1):360. doi: 10.1186/s12913-017-2302-4 (accessed 1 December, 2017).

For peer review only - http://bmjopen.bmj.com/site/about/guidelines.xhtml

49. Ahankari A, Bapat S, Myles P, et al. Factors associated with preterm delivery and low birth weight: a study from rural Maharashtra, India. *F1000Res*2017;6:72. doi: 10.12688/f1000research.10659.1 1 (accessed 1 December 2017).

50. Rakesh PS. Prevalence of anemia in Kerala State, Southern India - a systematic review.*JClinDiagn Res* 2017;11(5):LE01-LE04. doi: 10.7860/JCDR/2017/24681.9951(accessed 1 December 2017).

51. UNICEF. *National Factsheet, Coverage Evaluation Survey, 2009*. New Delhi: UNICEF India Country Office; 2010. http://www.indiawaterportal.org/sites/indiawaterportal.org/files/National%20Factsheet_Coverage%20Evaluation%20Survey_UNICEF_2009.pdf (accessed 25 November 2017).

52. Sebastian T, Yadav B, Jeyaseelan L, et al. Small for gestational age births among South Indian women: temporal trend and risk factors from 1996 to 2010. *BMC Pregnancy and Childbirth*2015;15:7. doi:10.1186/s12884-015-0440-4 (accessed 1 December 2017).

53. Muhihi A, Sudfeld CR, Smith ER, et al. Risk factors for small-for-gestational-age and preterm births among 19,269 Tanzanian newborns. *BMC Pregnancy*

54. Varela-Silva MI, Azcorra H, Dickinson F, Bogin B, Frisancho AR. Influence of maternal stature, pregnancy age, and infant birth weight on growth during childhood in Yucatan, Mexico: a test of the intergenerational effects hypothesis. *Am J Hum Biol* 2009; 21(5):657-63. doi: 10.1002/ajhb.20883 (accessed 6 December 2017)

55. Kaur M, Chauhan A, Manzar MD, et al. Maternal anemia and neonatal outcome: aprospective study on urban pregnant women. *J ClinDiagn Res* 2015;9(12):QC04-QC08. doi:10.7860/JCDR/2015/14924.6985 (accessed 1 December 2017).

56. Verma A, Shrimali L. Maternal body mass index and pregnancy outcome. J ClinDiagn Res 2012;6(9):1531-1533. doi:10.7860/JCDR/2012/4508.2551(accessed 1 December 2017).

57. Haider BA, Olofin I, Wang M, et al.Anemia, prenatal iron use, and risk of adverse pregnancy outcomes: systematic review and meta-analysis. *The BMJ*2013;346:f3443. doi:10.1136/bmj.f3443(accessed 1 December 2017).

*Childbirth*2016;16:110. doi:10.1186/s12884-016-0900-5(accessed 1 December 2017).

58. Mahajan SD, Singh S, Shah P, et al.Effect of maternal malnutrition and anemia on the endocrine regulation of fetal growth.*Endocr Res* 2004; 30(2):189-203.

59. Tandu-UmbaB, Mbangama AM. Association of maternal anemia with other risk factors in occurrence of Great obstetrical syndromes at university clinics, Kinshasa, DR Congo. *BMC Pregnancy Childbirth* 2015; *15:* 183. doi: 10.1186/s12884-015-0623-z(accessed 1 December 2017).

60. Ramakrishnan U, Grant F, Goldenberg T, et al. Effect of women's nutrition before and during early pregnancy on maternal and infant outcomes: a systematic review.PaediatrPerinatEpidemiol, 26: 285–301. doi:10.1111/j.1365-3016.2012.01281.x (accessed 1 December 2017).

61. Kalk P, Guthmann F, Krause K, et al.Impact of maternal body mass index on neonatal outcome.*Eur J Med Res* 2009 May 14; 14(5): 216-22.

62. Nair M, Choudhury M, Choudhury S, Kakoty S, Sarma U, Webster P et al. Association between maternal anaemia and pregnancy outcomes: a cohort study in Assam, India. BMJ Global Health 2016; 1:e000026. doi:10.1136/bmjgh-2015-000026

63. Sankar MJ, Neogi SB, Sharma J, et al. State of newborn health in India. J Perinato 2016; 36(3), S3–S8. doi: 10.1038/jp.2016.183 (accessed 1 December 2017).

For peer review only

1 **Figure 1: Categorical anemia (gm/dl)in included women by body mass index (kg/m^2), over year of enrolment**

4 **Figure1: Pic**

For peer review only

For peer review only - http://bmjopen.bmj.com/site/about/guidelines.xhtml

Figure 2: Proportions of Anemia (gm/dl) & body mass index (kg/m^2) interaction in included women, over year of enrollment

Figure2: Pic

For peer review only

For peer review only - http://bmjopen.bmj.com/site/about/guidelines.xhtml

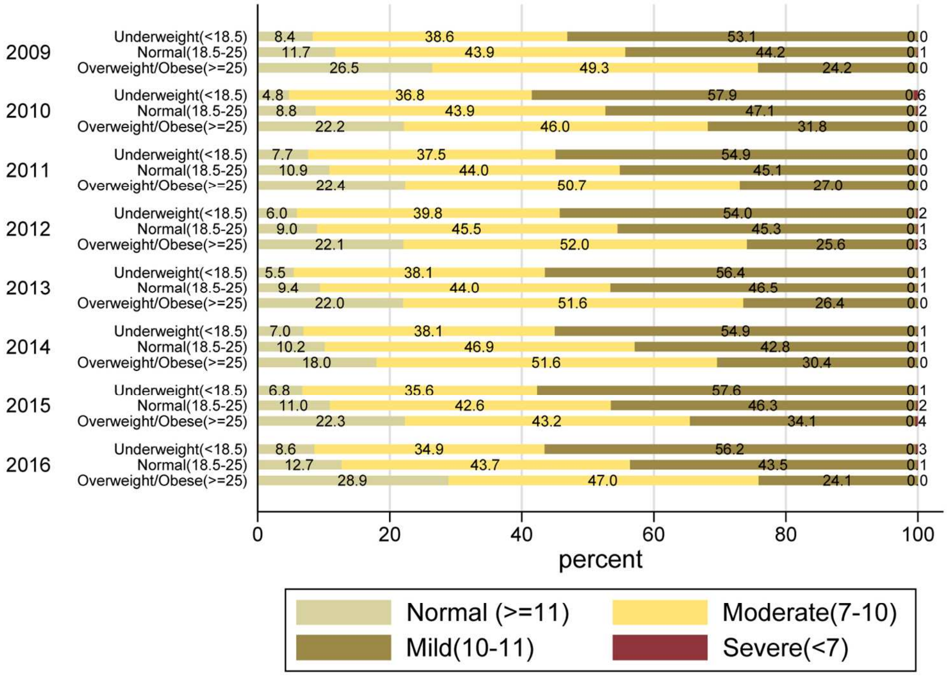

Figure 1: Categorical anemia (gm/dl)in included women by body mass index (kg/m2), over year of enrolment

139x101mm (300 x 300 DPI)

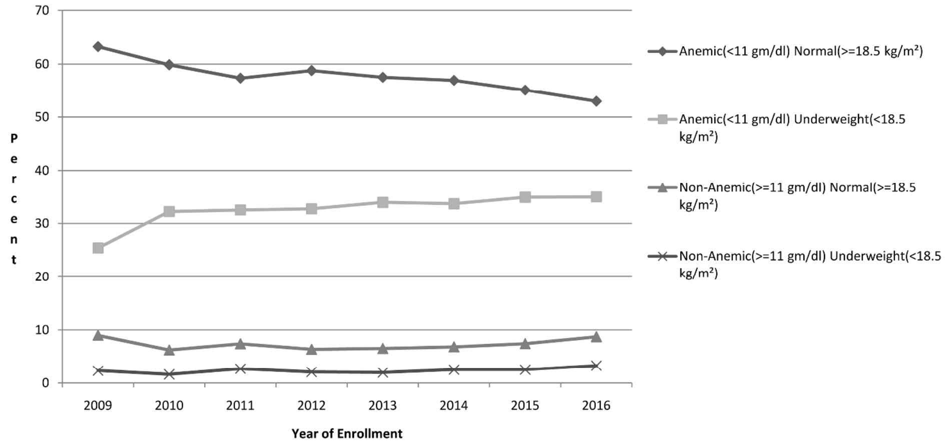

Proportions of Anemia (gm/dl) & body mass index (kg/m2) interaction in included women, over year of enrollment

247x114mm (300 x 300 DPI)

STROBE Statement—Checklist of items that should be included in reports of ***cohort studies***

	Item No	Recommendation
**Title and abstract**	1	(*a*) Indicate the study's design with a commonly used term in the title or the abstract ☑ - ***Page 1***
		(*b*) Provide in the abstract an informative and balanced summary of what was done and what was found ☑ - ***Page 5***
**Introduction**		
Background/rationale	2	Explain the scientific background and rationale for the investigation being reported ☑ - ***Page 8***
Objectives	3	State specific objectives, including any prespecified hypotheses ☑ - ***Page 8-9***
**Methods**		
Study design	4	Present key elements of study design early in the paper ☑ - ***Page 9***
Setting	5	Describe the setting, locations, and relevant dates, including periods of recruitment, exposure, follow-up, and data collection ☑ - ***Page 9***
Participants	6	(*a*) Give the eligibility criteria, and the sources and methods of selection of participants ☑ - ***Page 9-10***
		(*b*) For matched studies, give matching criteria and number of exposed and unexposed N/A
Variables	7	Clearly define all outcomes, exposures, predictors, potential confounders, and effect modifiers. Give diagnostic criteria, if applicable ☑ - ***Page 11 & 12***
Data sources/ measurement	8*	For each variable of interest, give sources of data and details of methods of assessment (measurement). Describe comparability of assessment methods if there is more than one group ☑ - ***Page 10 & 11***
Bias	9	Describe any efforts to address potential sources of bias ☑ - ***Page 10 & 11***
Study size	10	Explain how the study size was arrived at - N/A
Quantitative variables	11	Explain how quantitative variables were handled in the analyses. If applicable, describe which groupings were chosen and why ☑ - ***Page 10, 11 & 12***
Statistical methods	12	(*a*) Describe all statistical methods, including those used to control for confounding ☑ - ***Page 11 & 12***
		(*b*) Describe any methods used to examine subgroups and interactions ☑ - ***Page 12***
		(*c*) Explain how missing data were addressed ☑ - ***Page 12***
		(*d*) If applicable, describe analytical methods taking account of sampling strategy ☑ - ***Page 12***
		(*e*) Describe any sensitivity analyses - N/A
**Results**		
Participants	13*	(a) Report numbers of individuals at each stage of study—eg numbers potentially eligible, examined for eligibility, confirmed eligible, included in the study, completing follow-up, and analysed ☑ - ***Page 12 & 13***
		(b) Give reasons for non-participation at each stage - N/A
		(c) Consider use of a flow diagram - N/A
Descriptive data	14*	(a) Give characteristics of study participants (eg demographic, clinical, social) and information on exposures and potential confounders ☑ - ***Page 13***
		(b) Indicate number of participants with missing data for each variable of interest ☑ - ***Page 12 & 13***
Outcome data	15*	Report numbers of outcome events or summary measures ☑ - ***Page 15***
Main results	16	(*a*) Give unadjusted estimates and, if applicable, confounder-adjusted estimates and their

For peer review only

		precision (eg, 95% confidence interval). Make clear which confounders were adjusted for and why they were included ☑ - ***Page 17 thru 19***
		(*b*) Report category boundaries when continuous variables were categorized ☑ - ***Page 10 & 11***
		(*c*) If relevant, consider translating estimates of relative risk into absolute risk for a meaningful time period - N/A
Other analyses	17	Report other analyses done—eg analyses of subgroups and interactions, and sensitivity analyses ☑ - ***Page 17 & 19***
**Discussion**		
Key results	18	Summarise key results with reference to study objectives ☑ - ***Page 15 thru 19***
Limitations	19	Discuss limitations of the study, taking into account sources of potential bias or imprecision. Discuss both direction and magnitude of any potential bias ☑ - ***Page 7, 20-24***
Interpretation	20	Give a cautious overall interpretation of results considering objectives, limitations, multiplicity of analyses, results from similar studies, and other relevant evidence ☑ - ***Page 20 thru 24***
Generalisability	21	Discuss the generalisability (external validity) of the study results ☑ - ***Page 20 thru 24***
**Other information**		
Funding	22	Give the source of funding and the role of the funders for the present study and, if applicable, for the original study on which the present article is based ☑ - ***Page 27***

*Give information separately for exposed and unexposed groups.

Note: An Explanation and Elaboration article discusses each checklist item and gives methodological background and published examples of transparent reporting. The STROBE checklist is best used in conjunction with this article (freely available on the Web sites of PLoS Medicine at http://www.plosmedicine.org/, Annals of Internal Medicine at http://www.annals.org/, and Epidemiology at http://www.epidem.com/). Information on the STROBE Initiative is available at www.strobe-statement.org.

For peer review only

For peer review only - http://bmjopen.bmj.com/site/about/guidelines.xhtml

BMJ Open

MATERNAL ANEMIA AND UNDERWEIGHT AS DETERMINANTS OF PREGNANCY OUTCOMES: COHORT STUDY IN EASTERN RURAL MAHARASHTRA, INDIA

Journal:	*BMJ Open*
Manuscript ID	bmjopen-2018-021623.R3
Article Type:	Research
Date Submitted by the Author:	23-Jun-2018
Complete List of Authors:	Patel, Archana; Lata Medical Research Foundation, Nagpur<br>Prakash, Amber; Lata Medical Research Foundation, Nagpur, Research Unit<br>Das, Prabir Kumar; Lata Medical Research Foundation, Nagpur<br>Gupta, Swarnim ; Lata Medical Research Foundation, Nagpur<br>Pusdekar, Yamini; Lata Medical Research Foundation, Nagpur<br>Hibberd, Patricia; Boston University School of Public Health; Boston University School of Medicine
**Primary Subject Heading**:	Public health
Secondary Subject Heading:	Global health, Epidemiology, Nutrition and metabolism
Keywords:	Body Mass Index, Anemia, Pregnancy, Neonatal Deaths, Malnutrition, Stillbirth

SCHOLARONE™
Manuscripts

only

MATERNAL ANEMIA AND UNDERWEIGHT AS DETERMINANTS OF PREGNANCY OUTCOMES: COHORT STUDY IN EASTERN RURAL MAHARASHTRA, INDIA

Archana Patel[1], Amber Abhijeet Prakash*[1], Prabir Kumar Das[1], Swarnim Gupta[1], Yamini Pusdekar[1], Patricia L Hibberd[2]

1. Lata Medical Research Foundation, Nagpur, India

2. Boston University School of Public Health and Boston University School of Medicine, Boston, United Stated of America

First Author:

Archana Patel(MD PhD)

Program Director

Address: Lata Medical Research Foundation,

Vasant Nagar, Nagpur- 440022,

Maharashtra, India

Telephone: +0712-2249569

Email: dr_apatel@yahoo.com

*Second and Corresponding Author:

Amber Abhijeet Prakash(MSc)

Senior Statistician and Data Manager

Address: Lata Medical Research Foundation,

Vasant Nagar, Nagpur- 440022,

For peer review only

Maharashtra, India

Telephone: +0712-2249569

Email: rockman.blues21@gmail.com

Third Author:

Prabir Kumar Das(MD)

Country Coordinator

Address: Lata Medical Research Foundation,

Vasant Nagar, Nagpur- 440022,

Maharashtra, India

Telephone: +0712-2249569

Email: prabir_das23@rediffmail.com

Fourth Author:

Swarnim Gupta(PhD)

Nutrition Consultant

Address: Lata Medical Research Foundation,

Vasant Nagar, Nagpur- 440022,

Maharashtra, India

Telephone: +0712-2249569

Email: gupta.swarnim@gmail.com

Fifth Author:

For peer review only

Yamini Pusdekar(MD)

Research Co-ordinator

Address: Lata Medical Research Foundation,

Vasant Nagar, Nagpur- 440022,

Maharashtra, India

Telephone: +0712-2249569

Email: dryaminipusdekar27@gmail.com

Sixth Author:

Patricia L Hibberd(MD PhD)

Chair, Department of Global Health

Professor of Global Health, Boston University School of Public Health

Professor of Medicine, Boston University School of Medicine

372 Crosstown Building

801 Massachusetts Avenue

Boston, MA 02118

Email:plh0@bu.edu

Telephone:617-414-1260

WORD COUNT: 6,300

For peer review only

For peer review only - http://bmjopen.bmj.com/site/about/guidelines.xhtml

COPYRIGHT

The Corresponding Author has the right to grant on behalf of all authors and does grant on behalf of all authors, an exclusive license (or non exclusive for government employees) on a worldwide basis to the BMJ Publishing Group Ltd to permit this article (if accepted) to be published in BMJ editions and any other BMJPGL products and sublicenses such use and exploit all subsidiary rights, as set out in our license

For peer review only

For peer review only - http://bmjopen.bmj.com/site/about/guidelines.xhtml

ABSTRACT

Objectives: To study the trend in the prevalence of anemia and low BMI among pregnant women from Eastern Maharashtra and evaluate if low BMI and anemia affect pregnancy outcomes.

Design: Prospective observational cohort study.

Setting: Catchment areas of 20 rural primary health centers in 4 eastern districts of Maharashtra State, India.

Participants: 72,750 women from the Nagpur site of Maternal and Newborn Health Registry of NIH's Global Network, enrolled from 2009 to 2016.

Main outcome measures: Mode of delivery, pregnancy related complications at delivery, stillbirths, neonatal deaths and low birth weight (LBW) in babies.

Results: Over 90% of the women included in the study were anemic and over a third were underweight (BMI<18 kg/m2) and with both conditions. Mild anemia at any time during delivery significantly increased the risk (Risk ratio; 95% confidence interval (RR;(95% CI)) of stillbirth (1.3(1.1 - 1.6)), neonatal deaths (1.3(1 - 1.6)) and LBW babies (1.1(1- 1.2)) . The risks became even more significant and increased further with moderate/severe anemia any time during pregnancy for stillbirth (1.4(1.2 - 1.8)), neonatal deaths (1.7(1.3 - 2.1)) and LBW babies

For peer review only - http://bmjopen.bmj.com/site/about/guidelines.xhtml

(1.3(1.2 - 1.4))., . Underweight at anytime during pregnancy increased the risk of neonatal deaths (1.1(1 - 1.3)) and LBW babies (1.2;(1.2 - 1.3)).The risk of having stillbirths (1.5;(1.2 - 1.8)), neonatal deaths (1.7;(1.3 - 2.3)) and LBW babies (1.5;(1.4 - 1.6)) was highest when - the anemia and underweight co-existed in the included women. Obesity/overweight during pregnancy increased the risk of maternal complications at delivery (1.6;(1.5 - 1.7)) and of cesarean section (1.5;(1.4 - 1.6)) and reduced the risk of LBW babies 0.8(0.8 - 0.9)).

Conclusion: Maternal anemia is associated with enhanced risk of stillbirth, neonatal deaths and LBW. The risks increased if anemia and underweight were present simultaneously.

Trial registration: Registered at ClinicTrials.gov (NCT01073475)

Keywords: Body Mass Index, anemia, pregnancy, neonatal deaths, malnutrition, stillbirth, low birth weight

For peer review only - http://bmjopen.bmj.com/site/about/guidelines.xhtml

For peer review only

STRENGTHS AND LIMITATIONS

1. This is a large prospective study with a sample size of 72,750 pregnant women collected over an 8year period in the target geographical region of Eastern Maharashtra, focusing on the synergistic effects of low body mass index (BMI) and anemia anytime in pregnant women, on stillbirths, neonatal death and low birth weight in newborns, which may have been overlooked in prior research.
2. Dietary practices and details of compliance with iron supplementation data was not collected in this study.
3. Pre-pregnancy data on BMI and anemia were not available in this study. The broad of range of gestational age during first visit mitigates the exclusive pathophysiologic effect of undernutrition in women with low BMI due to under-nutrition, on pregnancy outcomes.
4. The hemoglobin levels were measured using Sahli's method, which may not be accurate due to subjectivity of the method, poor sensitivity and lack of reliability.

For peer review only - http://bmjopen.bmj.com/site/about/guidelines.xhtml

INTRODUCTION

Malnutrition is a serious underlying cause for child and maternal deaths around the globe. Undernutrition during pregnancy restricts fetal growth, contributing to about 800,000 neonatal and 400,000 infant deaths, and 20% of stunting in the first 2 years of the child's life, as well as 20% of maternal deaths at delivery. [1-3]The Sustainable Development Goals (SDG) bring explicit attention to nutrition, including the World Health Assembly target to reduce anemia in women of ages 15-49 years by 50% by 2025.[4-5] Since India has largest number of neonatal, infant and under age 5 children deaths in the world as well as high rates of stunting and growth faltering,[6-8], it is particularly timely to understand maternal nutritional status as a risk factor for maternal and childhood adverse outcomes of pregnancy in the Indian population.

Two important indicators of maternal nutrition are Body Mass Index (BMI) and anemia, both of which can affect health of a mother and her fetus. Much attention has been focused on complications of maternal overweight and obesity (BMI>25 kg/m^2) including gestational hypertension, pre-eclampsia, macrosomia, early induction of labor and need for caesarian deliveries and currently apply mostly to high but also recognized increasingly in middle-income countries, including India.[9]However, in rural India, undernutrition (BMI<18.5 kg/m^2) predominates and is associated with low birth weight (LBW, <2.5kg) and preterm deliveries (<37 weeks of gestation). Pre-pregnancy BMI is most indicative of maternal undernutrition but this data is rarely available when a woman enrolls in the ante-natal clinics in many developing countries including India, where the first ante-natal visit is often after the first trimester. [10] Although the pathophysiology of low BMI early in pregnancy may differ from the same later in

For peer review only - http://bmjopen.bmj.com/site/about/guidelines.xhtml

pregnancy (fetal growth faltering, oligohydramnios etc), there is little information, if any, on the impact of low BMI recorded at the first ante-natal visit regardless of GA, on maternal and fetal outcomes. [11] This information would help to identify high risk pregnant women at their first ante-natal visit. Similarly, maternal anemia, as a result of undernutrition and infections coexist with low BMI, particularly in Indian women of reproductive age. In the 2015 National Family and Health Survey (NFHS), 23% of Indian women had a BMI less than 18.5 kg/m^2 and 53% were anemic (Hemoglobin < 11 gm/dL). [12] Even higher rates were reported in rural areas of the state of Maharashtra. [13] Maternal anemia is also associated with post-partum hemorrhage, LBW, small for gestational age (SGA) babies and perinatal death.[14] However, there is minimal information on the consequences of the combination of low BMI and anemia in pregnant women in rural Maharashtra and whether women with this dual burden should be targeted for specific interventions. We used data from an ongoing Maternal and Newborn Health Registry (MNHR) based in rural areas of Nagpur, Maharashtra, India to (1) describe the prevalence and trends of maternal anemia and low BMI from 2009 to 2016 (2) describe the maternal demographic characteristics associated with different levels of maternal hemoglobin and BMI from 2009-2016, (3) evaluate whether low BMI and anemia were independent or synergistic risk factors for adverse pregnancy outcomes between 2009 and2016, (4) investigate whether low BMI and anemia had additive or synergistic effects on poor birth outcomes.

METHODS

Study design and settings

For peer review only - http://bmjopen.bmj.com/site/about/guidelines.xhtml

The present study utilizes data collected from the Nagpur, India site of MNHR, which is a population-based registry established in 2009 by the Global Network (GN) with support from *Eunice Kennedy Shriver* National Institute of Child Health and Human Development (NICHD), United States of America (USA).The MNHR is an observational, prospective cohort operating in seven different sites around the world including two sites, in India. The Nagpur site comprises of 20study clusters in the 4 districts of Nagpur, Bhandara, Chandrapur and Wardha in Eastern Maharashtra, India. Geographical areas surrounding government primary health centers (PHC) that have approximately 900 deliveries per year constitute a study cluster. The MNHR aims to recruit all consenting women residing within the clusters, as early as possible during their pregnancy and then to follow them until 42 days post-partum. The MNHR methods have been published elsewhere. [15]The time period of this study was fromJune2009 to December 2016.

Patient and Public Involvement

The research questions for this study were developed to evaluate frequent reports of tiredness and weakness by the pregnant women enrolled in the prospective MNHR at the Nagpur site in rural Eastern Maharashtra and to further review the reported high prevalence of maternal anemia in the Indian Demographic Health Surveys. Specifically, we wanted to determine whether rates of anemia had decreased over time, as a result of an active national iron supplementation program, and, whether in our prospective cohort, these rates had an impact on pregnancy outcomes. We also wanted to study whether low BMI, common in our study subjects, further exacerbated the impact of anemia in pregnancy on perinatal outcomes. Since we intended to address these questions in a secondary data analysis using de-identified data in the MNHR, patients and public were not involved in the design of this study. The results will not be

For peer review only - http://bmjopen.bmj.com/site/about/guidelines.xhtml

disseminated to study participants but are intended for additional research to find ways to reduce maternal undernutrition (low BMI) and anemia, as well as to inform health policies on pre-pregnancy and maternal nutrition.

Participants

The participants in this study, were all consenting pregnant women residing in the 20 clusters of the Nagpur GN site, enrolled within the study period, who completed data collection(i.e., had delivery outcomes and a completed 42 days post-partum follow-up) at the time of analyses. Women who had medical termination of pregnancy (MTP) (premature expulsion of a non-viable fetus before 20 weeks of gestation), miscarriages, multiple fetuses or had extreme/missing values for one or more study variables of interest were excluded from the analyses. Early enrollment of the participants was ensured through active surveillance in the study clusters by Nagpur site MNHR public health staff. The enrolled women were followed through to a perinatal visit after delivery (within a week) and to a follow up visit around 42 days after birth. Maternal and infant data on study variables and outcomes, were recorded for the enrollees.

Data collection procedures

Briefly, data were collected by trained registry administrators (RA) via standardized forms. RAs included medical officers and auxiliary nurse-midwives (ANM) employed at PHCs and sub-centers. A two-day training for data collection was provided to the Registry Administrators (RAs), followed by a refresher training every six months, and unscheduled special trainings as needed. The RAs returned filled in forms which were manually scrutinized for errors prior to data entry at Lata Medical Research Foundation (LMRF), Nagpur. After entry, data were

For peer review only - http://bmjopen.bmj.com/site/about/guidelines.xhtml

transmitted to the GN data management center (Research Triangle Institute, Durham, North Carolina, USA) where digital data checks were performed on a monthly basis. The data management center forwarded edit, monitoring and performance reports to the Nagpur site team for data cleaning and management. The data were continually reviewed for quality by NICHD appointed data monitoring committee and by the Nagpur site data collection team.

Study variables

At the first antenatal visit, information regarding the enrolled women's, characteristics including age, education, parity, height, weight and hemoglobin was recorded. Weight to nearest 100grams using a spring balance and height to nearest centimeter was measured using a non-flexible measuring tape fixed to a wall. Body mass index (BMI) was calculated by dividing weight in kilograms (kg) by squared height in meters. Standard World Health Organization (WHO) BMI classifications[16] were used to categorize the enrollees as underweight (BMI<18.5 Kg/m^2), normal (BMI 18.5-24.9 Kg/m^2), or as overweight/ obese (BMI≥25Kg/m^2).Hemoglobin was estimated within two weeks of enrollment using Sahli's method.[17]A concentration of hemoglobin (Hb) level < 11 g/dL indicated presence of anemia. Based on WHO classification [18] hemoglobin readings were used to define the severity of anemia as normal (≥11g/dL), mild (10<11 g/dL) and moderate/severe (<10 g/dL).BMI and anemia were categorized as ordinal variables for assessing their rates in this study population in consistency with standard public health practices. A 4 level combination variable for BMI-anemia was defined as based on anemia -hemoglobin<11(g/dL) (anemic) vs. ≥ 11 (g/dL) (non-anemic) and BMI<18.5 (Kg/m2) (underweight) vs ≥ 18.5 (Kg/m2) (normal or not underweight), creating 'anemic-normal', 'anemic-underweight', 'non anemic-normal' and 'non anemic-underweight'. Mother's age was

For peer review only - http://bmjopen.bmj.com/site/about/guidelines.xhtml

recorded at the time of enrolment. Maternal education of up to 4 grades was considered 'primary', grade 5 to grade 10 as 'secondary' and more than grade 10 as 'university'. Parity was recorded as whole numbers zero or more, depending on if the woman had been pregnant before enrollment or in pregnancy had carried a fetus to 20 weeks of gestation, or not.

Study Outcomes

The impact of 3 level variables of anemia (normal, mild, moderate/severe) and BMI (underweight, normal, overweight/obese) was assessed on rates of mode of delivery, any pregnancy related complications at delivery, and, post-delivery birth outcomes. While we did not assess the determinants of maternal mortality as maternal mortality rates were low, we described the causes of death with respect to the level of anemia and BMI status. Mode of delivery was recorded as cesarean section(CS) or non-cesarean (vaginal, assisted vaginal).Any pregnancy related complications at delivery were recorded as presence of one or more of the following; obstructed/prolonged labor, severe antepartum hemorrhage (APH), severe postpartum hemorrhage (PPH), hypertension, pre-eclampsia, and oblique/abnormal lie. Birth outcomes such as stillbirth, neonatal deaths (deaths of neonates from birth to 28 days of life) and low birth weight (LBW) rates were assessed. Stillbirth rates were calculated as number of stillbirths per 1000 total births while neonatal death rates were calculated as number of neonatal deaths per 1000 live births. Birth weight was measured within 24 hours of birth by using either a pan spring or a pan digital weighing scale. LBW was defined as neonatal birth weight less than 2500 grams.

For peer review only

For peer review only - http://bmjopen.bmj.com/site/about/guidelines.xhtml

Statistical Analyses

Data were analyzed using the STATA 13.1 statistical package (Stata Corp, College Station, Texas, USA). Study variables of interest and their combinations, with years of enrollment were cross tabulated in Stata and were transformed to graphs either in Stata itself or in Microsoft Excel. Pearson's correlation coefficient was used to study correlation between hemoglobin concentrations and BMI values and was tested for significance. A univariate multinomial logistic regression, adjusting for clustering was used to identify association between the WHO levels of anemia and BMI categories. Significance of trends over the progression of years of enrollment in any of the study variables was determined using Cuzick's non-parametric test [19] for trend across ordered groups. The year-wise rates of the outcomes (CS, pregnancy related complication and LBW) were determined for each level of anemia and category of BMI. Stillbirths and neonatal death rates were calculated per 1000 total births and per 1000 live births respectively. To assess the association of maternal demographic characteristics, anemia and BMI with the study outcomes the data was divided in two data sets, one which included participants who had their first ante-natal visit ≤ 20 weeks of GA and the second set that included participants who had their first ante-natal visit after 20 weeks of GA. Both data sets were analyzed using multivariable regression analyses solved with generalized estimating equations (GEE), adjusting for clustering within the PHCs. For the first set of regression models, explanatory variables included demographic characteristics - mother's age, education level and parity, along with the 3 levels of anemia and 3 categories of BMI, as independent variables while dependent variables included all 5 study outcomes. The regressions were also performed using hemoglobin and BMI as continuous variables and compared with the results when these variables were considered as ordinal variables. The interaction of hemoglobin and BMI was also assessed. A second set of

For peer review only - http://bmjopen.bmj.com/site/about/guidelines.xhtml

model used demographic characteristics along with the constructed anemia-BMI variable ('anemic-normal', 'anemic-underweight', 'non anemic-normal' and 'non anemic-underweight') as explanatory variables for 3 post-natal outcomes–stillbirths, neonatal death and LBW. These models assessed the impact of anemia or under-weight either alone or in combination on the 3 post-natal outcomes, as it is relevant from the public health viewpoint to understand the risks of adverse outcomes when a combination of co-morbidities such as anemia and underweight co-exist.

RESULTS

Participants

During the study period, 72,750 women had delivery outcomes and complete follow up through day 42 post-partum was completed. Women with invalid or missing data on BMI or hemoglobin (N=764) and miscarriage/MTP/multiple gestation (N=4412) were excluded.

Baseline maternal characteristics

Almost 91% of the included women were anemic (65811/72750), over a third were underweight (25571/72750, 35.1%), and over a third were both anemic and underweight(23867/72750, 32.8%). Anemia was severe in less than 0.2% and moderate in nearly 48% women. The gestational age at the first antenatal visit was ≤20 weeks for 72%, 21-28 weeks for 15% and > 28 weeks for 13% of the study population. The study population mostly aged 20-29 years (93%); about half were primiparous 48% vs. multiparous, 52%. As shown in Table 1, over time there

For peer review only - http://bmjopen.bmj.com/site/about/guidelines.xhtml

was an increase in pregnant women in the older age groups>29 over and those who completed higher levels of education in later years (2015-2016). For parity, there was a reduction in proportions of parity>2 in 2016. The levels of anemia showed no significant changes over time. The proportion of women who were just underweight (28% to 38%) (Figure 1), and, underweight with anemia (25% to 35%) (Figure 2) increased over time. We found a highly significant correlation(r= 0.2; P<0.001) between hemoglobin and BMI. Multinomial logistic regression with normal hemoglobin concentrations as reference, showed highly significant (P<0.001) associations between women who were underweight and those with moderate/severe anemia (Risk Ratio (RR):1.9, 95% Confidence Interval (CI):1.6-2.2) as well as those with mild anemia (RR:1.3, 95% CI:1.2-1.5).

For peer review only - http://bmjopen.bmj.com/site/about/guidelines.xhtml

Table 1: Baseline Characteristics of women in the study, by year of enrollment

Characteristics	Years								
	**2009**<br>N (%)<br>6433 (100)	**2010**<br>N (%)<br>10447 (100)	**2011**<br>N (%)<br>9626 (100)	**2012**<br>N (%)<br>9905 (100)	**2013**<br>N (%)<br>9496 (100)	**2014**<br>N (%)<br>9454 (100)	**2015**<br>N (%)<br>10108 (100)	**2016**<br>N (%)<br>7281 (100)	**Total**<br>N (%)<br>72750 (100)
Mother's Age (years)*									
<20	131 (2)	209 (2)	181 (1.9)	188 (1.9)	190 (2)	193 (2)	209 (2.1)	157 (2.2)	1458 (2)
20-29	6044 (94)	9867 (94.4)	9056 (94.1)	9269 (93.6)	8834 (93)	8743 (92.5)	9266 (91.7)	6640 (91.2)	67719 (93.1)
>29	258 (4)	371 (3.6)	389 (4)	448 (4.5)	472 (5)	518 (5.5)	633 (6.3)	484 (6.6)	3573 (4.9)
Mother's Education (grades)*									
Primary or Less (<=4)	1476 (22.9)	2281 (21.8)	1846 (19.2)	1927 (19.5)	1882 (19.8)	693 (7.3)	633 (6.3)	329 (4.5)	11067 (15.2)
Secondary(5-10)	3814 (59.3)	6210 (59.4)	5818 (60.4)	5880 (59.4)	5432 (57.2)	4962 (52.5)	5101 (50.5)	3522 (48.4)	40739 (56)
University(>10)	1139 (17.7)	1956 (18.7)	1958 (20.3)	2071 (20.9)	2172 (22.9)	3768 (39.9)	4358 (43.1)	3415 (46.9)	20837 (28.6)
Missing	4 (0.1)	0 (0)	4 (0)	27 (0.3)	10 (0.1)	31 (0.3)	16 (0.2)	15 (0.2)	107 (0.1)
Parity*									
Nulliparous	2905 (45.2)	5118 (49)	4706 (48.9)	4817 (48.6)	4384 (46.2)	4530 (47.9)	4967 (49.1)	3598 (49.4)	35025 (48.1)
1-2	3300 (51.3)	5032 (48.2)	4669 (48.5)	4827 (48.7)	4907 (51.7)	4721 (49.9)	4888 (48.4)	3544 (48.7)	35888 (49.3)
>2	228 (3.5)	297 (2.8)	249 (2.6)	261 (2.6)	204 (2.1)	197 (2.1)	234 (2.3)	137 (1.9)	1807 (2.5)
Missing	0 (0)	0 (0)	2 (0)	0 (0)	1 (0)	6 (0.1)	19 (0.2)	2 (0)	30 (0)
Anemia (gm/dl)									
Moderate/Severe(<10)	2962 (46)	5283 (50.6)	4620 (48)	4730 (47.8)	4682 (49.3)	4418 (46.7)	5058 (50)	3457 (47.5)	35210 (48.4)
Mild(10<11)	2743 (42.6)	4341 (41.6)	4033 (41.9)	4337 (43.8)	4006 (42.2)	4152 (43.9)	4041 (40)	2948 (40.5)	30601 (42.1)
Normal(>=11)	728 (11.3)	823 (7.9)	973 (10.1)	838 (8.5)	808 (8.5)	884 (9.4)	1009 (10)	876 (12)	6939 (9.5)
Body Mass Index (kg/m^2) *									
Underweight(<18.5)	1784 (27.7)	3537 (33.9)	3391 (35.2)	3450 (34.8)	3411 (35.9)	3424 (36.2)	3786 (37.5)	2788 (38.3)	25571 (35.1)
Normal(18.5-24.9)	4426 (68.8)	6558 (62.8)	5931 (61.6)	6061 (61.2)	5699 (60)	5619 (59.4)	5789 (57.3)	4095 (56.2)	44178 (60.7)
Overweight or Obese(≥25)	223 (3.5)	352 (3.4)	304 (3.2)	394 (4)	386 (4.1)	411 (4.3)	533 (5.3)	398 (5.5)	3001 (4.1)
Anemia(gm/dl)-BMI(kg/m^2) factors									
Anemic(<11)-Normal(>=18.5)	4070 (63.3)	6256 (59.9)	5522 (57.4)	5823 (58.8)	5463 (57.5)	5384 (56.9)	5569 (55.1)	3857 (53)	41944 (57.7)
Anemic(<11)-Underweight(<18.5)	1635 (25.4)	3368 (32.2)	3131 (32.5)	3244 (32.8)	3225 (34)	3186 (33.7)	3530 (34.9)	2548 (35)	23867 (32.8)
Non-Anemic(>=11- Normal(>=18.5)	579 (9)	654 (6.3)	713 (7.4)	632 (6.4)	622 (6.6)	646 (6.8)	753 (7.4)	636 (8.7)	5235 (7.2)
Non-Anemic(>=11)-Underweight(<18.5)	149 (2.3)	169 (1.6)	260 (2.7)	206 (2.1)	186 (2)	238 (2.5)	256 (2.5)	240 (3.3)	1704 (2.3)
Body Mass Index (kg/m^2) [Mean/SD/IQR]	20.1/2.6/3.4	19.7/2.6/3.2	19.6/2.6/3.4	19.7/2.7/3.5	19.7/2.8/3.6	19.7/2.9/3.6	19.7/3/3.7	19.7/3/3.7	19.7/2.8/3.6
Hemoglobin (gm/dl) [Mean/SD/IQR]	9.8/0.9/1	9.7/0.9/1	9.7/0.8/1.1	9.8/0.8/1	9.9/0.8/0.8	9.9/0.8/0.8	9.9/0.8/1	9.9/0.9/1	9.8/0.8/1

* Highly significant (P<0.001) non parametric trend over years of enrollment

For peer review only - http://bmjopen.bmj.com/site/about/guidelines.xhtml

Maternal Mortality

There were 29 maternal deaths in the study population, 20 of whom had severe anemia. Moderate/Severe anemia was identified as a primary cause of death in 1 and as a co-morbid condition in 19. Of the total 29 deaths, anemia and underweight was observed in 8, anemia alone in 19, underweight alone in 1, and overweight/obese in 2. Reasons for maternal deaths, were hemorrhage (n=9, 31%), preeclampsia/eclampsia (n=7, 24%), infection (n=6, 21%), other/missing (n=7, 22%).

Study outcomes by years of enrollment

The rates of stillbirths, neonatal deaths and LBW babies increased with increasing severity of anemia, with lower BMI, and, if anemia and under-weight co-existed as compared to either condition alone, consistently in all years of enrollment. Rates of LBW babies decreased in overweight/obese mothers. The rates of CS and pregnancy related complications increased with increasing BMI. Rates of CS increased over time for all levels of anemia and BMI(Table 2).

For peer review only

For peer review only - http://bmjopen.bmj.com/site/about/guidelines.xhtml

Table 2: Rates of study with respect to levels of anemia and body mass index categories, by year of enrolment

	Year of Enrollment								
	**2009**	**2010**	**2011**	**2012**	**2013**	**2014**	**2015**	**2016**	**Total**
**Variables**	**Rates of women experiencing cesarean sections**								
Anemia (gm/dl)*									
Moderate/Severe(<10)	15.67	16.83	17.19	19.53	20.91	23.74	24.73	28.67	20.85
Mild(10-11)	16	17.97	19.69	21.51	24.84	26.42	31.08	33.58	23.8
Normal(>=11)	18.41	26.12	24.87	26.13	31.44	32.01	37.07	39.61	29.8
Body Mass Index (kg/m^2)									
Underweight(<18.5)	15.08	15.13	16.13	17.65	19.2	21.61	23.38	26.79	19.5
Normal(18.5-24.9)	15.95	18.65	19.91	21.53	24.64	26.87	29.9	33.41	23.6
Overweight or Obese(≥25)	27.8	35.8	33.55	41.12	43.78	43.55	49.72	53.52	42.59
Anemic(HB< 11 gm/dl) & Underweight (BMI<18.5 kg/m^2)*	15.05	14.96	15.94	17.32	18.98	21.19	23.31	26.14	19.22
**Variables**	**Rates of women with pregnancy complications present at delivery**								
Anemia (gm/dl)*									
Moderate/Severe(<10)	14.58	14.1	12.03	14.76	14.72	10.5	10.7	10.85	12.78
Mild(10<11)	14.51	14.67	14.95	15.75	17.05	13.97	11.9	12.48	14.49
Normal(>=11)	16.62	21.51	16.65	17.42	22.15	18.1	15.86	14.27	17.73
Body Mass Index (kg/m^2)									
Underweight(<18.5)	13.85	12.58	11.53	14.03	14.07	10.25	10.46	10.26	12.04
Normal(18.5-24.9)	14.91	15.46	14.37	15.38	17.07	13.54	11.99	12.43	14.48
Overweight or Obese(≥25)	19.73	28.41	25.66	28.17	25.39	22.38	17.26	18.34	22.93
Anemic(HB< 11 gm/dl) & Underweight (BMI<18.5 kg/m^2)*	13.82	12.44	11.85	14.09	13.64	9.79	10.28	10.09	11.92
**Variables**	**Stillbirth rate per 1000 total births**								
Anemia (gm/dl)									
Moderate/Severe(<10)	29.37	25.55	22.29	23.89	26.27	21.05	23.33	26.61	24.54
Mild(10-11)	27.34	24.65	20.58	19.37	23.71	19.27	18.81	15.6	21.11
Normal(>=11)	12.36	18.23	15.42	16.71	16.09	16.97	14.87	15.98	15.85
Body Mass Index (kg/m^2)*									
Underweight(<18.5)	29.15	25.73	22.41	22.32	25.21	20.74	22.45	27.62	24.05
Normal(18.5-24.9)	25.76	24.7	19.9	20.13	24.21	19.93	19.52	17.34	21.5
Overweight or Obese(≥25)	22.42	11.36	23.03	30.46	18.13	12.17	20.64	10.05	18.33
Anemic(HB< 11 gm/dl) & Underweight (BMI<18.5 kg/m^2)*	29.97	25.83	23.32	23.43	25.74	21.34	23.23	27.86	24.68
**Variables**	**Neonatal death rate per 1000 live births**								
Anemia (gm/dl)									
Moderate/Severe(<10)	22.06	21.63	19.64	28.74	24.05	21.99	24.05	21.89	23.09
Mild(10<11)	17.54	16.08	15.44	16.74	19.03	18.26	17.72	16.47	17.15
Normal(>=11)	11.25	25.38	11.62	14.8	6.33	12.82	13.25	9.37	13.06
Body Mass Index (kg/m^2)									
Underweight(<18.5)	21.23	20.13	21.26	23.68	24.98	20.09	22.94	20.74	21.96
Normal(18.5-24.9)	18.19	19.28	14.14	21.86	18.13	19.25	19.04	16.42	18.38
Overweight or Obese(≥25)	13.95	20.53	27.68	15.96	13.37	17.54	17.54	18.13	17.97

For peer review only - http://bmjopen.bmj.com/site/about/guidelines.xhtml

Anemic(HB< 11 gm/dl) & Underweight (BMI<18.5 kg/m^2)	21.25	19.26	22.07	24.59	26.48	20.3	22.84	21.06	22.32
**Variables**	**Rates of low birth weight (<2500 g) babies**								
Anemia (gm/dl)									
Moderate/Severe(<10)	17.42	17.87	17.51	18.46	19.14	19.04	20.42	22.65	19.01
Mild(10-11)	16.99	14.37	14.63	13.53	14.5	14.72	16.98	18.69	15.35
Normal(>=11)	14.15	15.43	12.85	11.22	10.4	12.9	16.45	16.32	13.78
Body Mass Index (kg/m^2)*									
Underweight(<18.5)	19.79	18.63	17.9	19.59	20.14	19.28	21.61	22.74	19.92
Normal(18.5-24.9)	15.95	15.23	14.69	13.81	14.6	15.09	17.29	19.54	15.61
Overweight or Obese(≥25)	11.66	10.51	15.13	10.41	10.88	14.11	12.38	10.8	11.96
Anemic(HB< 11 gm/dl) & Underweight (BMI<18.5 kg/m^2)*	20.18	18.53	17.89	19.79	20.5	19.55	21.5	22.96	20.04

* Significant (P<0.01) non parametric trend over years of enrollment

For peer review only

For peer review only - http://bmjopen.bmj.com/site/about/guidelines.xhtml

Study Outcomes: Regression

The risks of CS and pregnancy related complications during delivery were significantly higher in non-anemic women vs. anemic women for both data sets. Mild anemia recorded ≤ 20 weeks had no impact on the outcomes, but when recorded after 20 weeks mild anemia increased the risk of stillbirths and neonatal deaths. Moderate/severe anemia increased the risk of neonatal deaths and LBW when recorded≤ 20 weeks and when present after 20 weeks it also increased the risk of stillbirths. (Table 3A, 3B). The rates of LBW were significantly higher in underweight women anytime during pregnancy (Table 3A, 3B, 3C).The risk of CS as well as pregnancy related complications were higher in women who were overweight/obese recorded anytime during pregnancy while the risk of LBW was reduced in the same group when recorded after 20 weeks of pregnancy (Table 3C). The risks of stillbirth, neonatal deaths and LBW were the highest when women were both anemic and underweight (Table 4).

Nulliparous women had a significantly higher risk for all five outcomes, higher mother's age (>29 years) was associated with stillbirth, neonatal deaths and LBW, while lower maternal education level (secondary or less) was associated with stillbirths, neonatal deaths and LBW. Women < 20 years were at lower risk for CS. Women with lower levels of education were also significantly less likely to have a CS or pregnancy related complication(Table 3C).

For peer review only - http://bmjopen.bmj.com/site/about/guidelines.xhtml

Table 3A: Risk factors for study outcomes, multivariable estimates (GA at enrolment ≤ 20 weeks)

	Study Outcomes				
**Characteristics**	**Cesarean Sections**<br><br>**N=11628, 24%**<br>**RR(95% CI)^**	**Pregnancy related Maternal complications**<br>**N=6762, 14%**<br>**RR(95% CI)^**	**Stillbirths**<br><br>**N=1104, 2.3%**<br>**RR(95% CI)^**	**Neonatal deaths (within 28 days of birth)**<br>**N=963, 2.0%**<br>**RR(95% CI)^**	**Low birth weight (<2500 gms)**<br>**N=8378, 18%**<br>**RR(95% CI)^**
Mother's Age (years)					
<20	0.83*(0.73 - 0.95)	0.83*(0.72 - 0.97)	0.73(0.47 - 1.12)	1.23(0.87 - 1.75)	1(0.88 - 1.15)
20-29	ref	Ref	Ref	ref	ref
>29	1.56**(1.44 - 1.68)	1.37**(1.22 - 1.53)	1.45*(1.13 - 1.86)	1.37*(1.03 - 1.80)	1.27**(1.15 - 1.40)
Mother's Education (grades)					
Primary or Less (<=4)	0.50**(0.46 - 0.54)	0.76**(0.70 - 0.83)	1.68**(1.39 - 2.04)	1.53**(1.24 - 1.87)	1.17**(1.08 - 1.25)
Secondary(5-10)	0.70**(0.67 - 0.73)	0.90**(0.85 - 0.94)	1.40**(1.21 - 1.61)	1.30**(1.12 - 1.52)	1.15**(1.09 - 1.21)
University(>10)	ref	Ref	Ref	ref	ref
Parity					
Nulliparous	1.33**(1.28 - 1.39)	2.00**(1.86 - 2.15)	1.32**(1.17 - 1.50)	1.57**(1.38 - 1.80)	1.38**(1.31 - 1.44)
1-2	ref	Ref	Ref	ref	ref
>2	0.49**(0.40 - 0.60)	0.80*(0.63 - 1.00)	1.50*(1.06 - 2.13)	1.62*(1.10 - 2.37)	0.99(0.85 - 1.17)
Anemia (gm/dl)					
Severe/Moderate (<10)	0.89**(0.84 - 0.95)	0.90*(0.83 - 0.98)	1.16(0.92 - 1.46)	1.37*(1.06 - 1.77)	1.24**(1.14 - 1.35)
Mild(10-11)	0.91*(0.86 - 0.97)	0.92*(0.85 - 0.99)	1.12(0.88 - 1.41)	1.16(0.90 - 1.50)	1.09(1.00 - 1.18)
Normal(>=11)	ref	Ref	Ref	ref	ref
Body Mass Index (kg/m^2)					
Underweight(<18.5)	0.81**(0.77 - 0.84)	0.83**(0.79 - 0.88)	1.08(0.96 - 1.22)	1.09(0.96 - 1.24)	1.18**(1.13 - 1.23)
Normal(18.5-24.9)	ref	Ref	Ref	ref	ref
Overweight or Obese(≥25)	1.64**(1.51 - 1.78)	1.44**(1.28 - 1.61)	1.19(0.84 - 1.67)	1.25(0.87 - 1.79)	0.91(0.79 - 1.04)

* P<0.05, ** P<0.001

^ Obtained by multivariable GEE regression models for each outcome, separately

For peer review only - http://bmjopen.bmj.com/site/about/guidelines.xhtml

Table 3B: Risk factors for study outcomes, multivariable estimates (GA at enrollment>20 weeks)

	Study Outcomes				
**Characteristics**	**Cesarean Sections N= 4816, 20% RR(95% CI)^**	**Pregnancy related Maternal complications N=3260, 14% RR(95% CI)^**	**Stillbirths N=488, 2% RR(95% CI)^**	**Neonatal deaths (within 28 days of birth) N=390, 1% RR(95% CI)^**	**Low birth weight (<2500 gms) N=3776, 16% RR(95% CI)^**
Mother's Age (years)					
<20	0.63*(0.46 - 0.86)	0.78(0.58 - 1.07)	1.28(0.66 - 2.49)	1.47(0.75 - 2.87)	1.17(0.92 - 1.48)
20-29	ref	Ref	Ref	ref	ref
>29	1.66**(1.48 - 1.86)	1.37**(1.18 - 1.60)	2.31**(1.70 - 3.13)	1.47(1.00 - 2.16)	1.29**(1.13 - 1.48)
Mother's Education (grades)					
Primary or Less (<=4)	0.45**(0.40 - 0.50)	0.61**(0.54 - 0.68)	1.51*(1.14 - 2.01)	1.56*(1.13 - 2.14)	1.08(0.98 - 1.19)
Secondary(5-10)	0.66**(0.62 - 0.71)	0.87**(0.80 - 0.94)	1.34*(1.05 - 1.71)	1.37*(1.04 - 1.80)	1.10*(1.02 - 1.19)
University(>10)	ref	Ref	Ref	ref	ref
Parity					
Nulliparous	1.51**(1.41 - 1.60)	1.98**(1.83 - 2.15)	1.25*(1.04 - 1.51)	1.31*(1.06 - 1.61)	1.31**(1.23 - 1.40)
1-2	ref	Ref	Ref	ref	ref
>2	0.42**(0.32 - 0.55)	0.74*(0.56 - 0.98)	0.89(0.56 - 1.42)	1.49(0.95 - 2.34)	1(0.84 - 1.20)
Anemia (gm/dl)					
Severe/Moderate (<10)	0.79**(0.72 - 0.87)	0.87*(0.78 - 0.97)	2.35**(1.56 - 3.53)	2.76**(1.73 - 4.42)	1.33**(1.19 - 1.49)
Mild(10-11)	0.87**(0.80 - 0.94)	0.87*(0.78 - 0.97)	1.81*(1.20 - 2.72)	1.73*(1.08 - 2.76)	1.07(0.96 - 1.20)
Normal(>=11)	ref	Ref	Ref	ref	ref
Body Mass Index (kg/m^2)					
Underweight(<18.5)	0.84**(0.77 - 0.91)	0.82**(0.74 - 0.90)	1.12(0.91 - 1.39)	1.12(0.88 - 1.42)	1.28**(1.19 - 1.38)
Normal(18.5-24.9)	ref	Ref	Ref	ref	ref
Overweight or Obese(≥25)	1.59**(1.45 - 1.74)	1.55**(1.38 - 1.73)	0.7(0.44 - 1.10)	1.03(0.67 - 1.60)	0.78*(0.68 - 0.91)

* P<0.05, ** P<0.001

^ Obtained by multivariable GEE regression models for each outcome, separately

For peer review only

Table 3C: Risk factors for study outcomes, multivariable estimates (all included women)

	Study Outcomes				
**Characteristics**	**Cesarean Sections<br>N=16693, 23%<br>RR(95% CI)^**	**Pregnancy related Maternal complications<br>N=10163, 14%<br>RR(95% CI)^**	**Stillbirths<br>N=1620, 2.2%<br>RR(95% CI)^**	**Neonatal deaths (within 28 days of birth)<br>N=1368, 1.9%<br>RR(95% CI)^**	**Low birth weight (<2500 gms)<br>N=12347, 17%<br>RR(95% CI)^**
Mother's Age (years)					
<20	0.80**(0.72 - 0.90)	0.83*(0.73 - 0.95)	0.88(0.62 - 1.25)	1.28(0.94 - 1.75)	1.04(0.92 - 1.16)
20-29	ref	Ref	Ref	ref	ref
>29	1.60**(1.50 - 1.70)	1.38**(1.26 - 1.50)	1.76**(1.45 - 2.12)	1.47**(1.18 - 1.83)	1.28**(1.18 - 1.38)
Mother's Education (grades)					
Primary or Less (<=4)	0.47**(0.44 - 0.50)	0.69**(0.64 - 0.74)	1.62**(1.38 - 1.89)	1.49**(1.26 - 1.77)	1.14**(1.08 - 1.21)
Secondary(5-10)	0.68**(0.66 - 0.70)	0.89**(0.85 - 0.93)	1.38**(1.22 - 1.57)	1.30**(1.14 - 1.49)	1.14**(1.09 - 1.19)
University(>10)	ref	Ref	Ref	ref	ref
Parity					
Nulliparous	1.38**(1.34 - 1.43)	2.00**(1.88 - 2.12)	1.31**(1.18 - 1.45)	1.48**(1.33 - 1.66)	1.35**(1.30 - 1.41)
1-2	ref	Ref	Ref	ref	ref
>2	0.45**(0.38 - 0.53)	0.76*(0.64 - 0.90)	1.22(0.93 - 1.62)	1.52*(1.14 - 2.04)	1(0.89 - 1.13)
Anemia (gm/dl)					
Severe/Moderate (<10)	0.87**(0.83 - 0.92)	0.89**(0.84 - 0.95)	1.43**(1.17 - 1.75)	1.67**(1.33 - 2.08)	1.26**(1.18 - 1.35)
Mild(10>11)	0.90**(0.86 - 0.94)	0.90*(0.85 - 0.96)	1.28*(1.05 - 1.57)	1.28*(1.02 - 1.60)	1.08*(1.01 - 1.15)
Normal(>=11)	ref	Ref	Ref	ref	ref
Body Mass Index (kg/m^2)					
Underweight(<18.5)	0.85**(0.82 - 0.88)	0.85**(0.81 - 0.88)	1.1(1.00 - 1.22)	1.12*(1.00 - 1.25)	1.21**(1.16 - 1.25)
Normal(18.5-24.9)	ref	Ref	Ref	ref	ref
Overweight or Obese(≥25)	1.57**(1.48 - 1.67)	1.48**(1.36 - 1.60)	0.92(0.70 - 1.20)	1.11(0.84 - 1.46)	0.84*(0.76 - 0.94)

* P<0.05, ** P<0.001

^ Obtained by multivariable GEE regression models for each outcome, separately

For peer review only - http://bmjopen.bmj.com/site/about/guidelines.xhtml

Table 4: Anemia-BMI factors for study outcomes, multivariable estimates

Characteristics	Study Outcomes		
	Stillbirths<br>N=1620, 2.2%<br>RR(95% CI)^	Neonatal deaths (within 28 days of birth)<br>N=1368, 1.9%<br>RR(95% CI)^	Low birth weight (<2500 gms)<br>N=12347, 17%<br>RR(95% CI)^
Mother's Age (years)			
<20	0.88(0.62 - 1.25)	1.28(0.94 - 1.75)	1.04(0.93 - 1.16)
20-29	Ref	Ref	ref
>29	1.75**(1.45 - 2.11)	1.48**(1.19 - 1.84)	1.27**(1.17 - 1.37)
Mother's Education (grades)			
Primary or Less (<=4)	1.64**(1.40 - 1.91)	1.52**(1.29 - 1.80)	1.16**(1.10 - 1.23)
Secondary(5-10)	1.40**(1.23 - 1.58)	1.32**(1.16 - 1.51)	1.15**(1.10 - 1.20)
University(>10)	Ref	Ref	ref
Parity			
Nulliparous	1.30**(1.18 - 1.44)	1.47**(1.31 - 1.64)	1.35**(1.29 - 1.40)
1-2	Ref	Ref	ref
>2	1.24(0.94 - 1.63)	1.56*(1.17 - 2.09)	1.02(0.91 - 1.15)
Anemia(gm/dl)-BMI(kg/m^2) factors			
Anemic(<11)-Normal(>=18.5)	1.30*(1.04 - 1.63)	1.54**(1.19 - 2.00)	1.22**(1.13 - 1.32)
Anemic(<11)-Underweight(<18.5)	1.47**(1.17 - 1.85)	1.74**(1.33 - 2.26)	1.49**(1.37 - 1.62)
Non-Anemic(>=11- Normal(>=18.5)	Ref	Ref	ref
Non-Anemic(>=11)-Underweight(<18.5)	0.96(0.62 - 1.48)	1.37(0.88 - 2.13)	1.37**(1.20 - 1.57)

* P<0.05, ** P<0.001

^ Obtained by univariate GEE regression models for each outcome, separately

For peer review only

For peer review only - http://bmjopen.bmj.com/site/about/guidelines.xhtml

DISCUSSION

Maternal malnutrition continues to be a silent emergency particularly in low middle-income countries (LMICs) like India. We attempted to understand through present study, not only the trends in the prevalence of anemia and low BMI over the eight years but the less understood combination of low maternal BMI and anemia with pregnancy related outcomes among rural women from Eastern Maharashtra. We found that more than 90% of the pregnant women were anemic, 35% were underweight (BMI< 18.5) and nearly a third had both conditions. It was alarming that the rates of anemia remained unchanged over eight years (2009-2016) and those of underweight increased. The majority of women had mild to moderate anemia, with severe anemia in less than 0.2%. While mild anemia recorded before 20 weeks of GA did not increase the risk of stillbirths, neonatal deaths or LBW, it increased the risk of stillbirths and neonatal deaths when recorded later in the pregnancy (>20 weeks). Moderate/severe anemia recorded anytime during pregnancy increased the risk of neonatal deaths and LBW. It also increased the risk of stillbirths when recorded later in pregnancy. In developing countries, women often enroll late in the antenatal care services. In our study population, approximately a third enrolled after 20 weeks of pregnancy, 90% of which were anemic and nearly half had moderate/severe anemia.

Although anemia occurring anytime during pregnancy is a risk factor for poor neonatal outcomes, anemia especially during the third trimester is an important factor in determining birth weight. Rapid fetal growth occurs in the third trimester, increasing the iron and other micronutrient requirement. This pathophysiology explains the association of third trimester hemoglobin levels with low birth weight and neonatal deaths.[20-21]

This study shows that a subtle public health action of early and aggressive management of anemia could lower the risk of adverse neonatal outcomes. Rates of underweight increased by

For peer review only - http://bmjopen.bmj.com/site/about/guidelines.xhtml

10% over eight years, and there was an increase in the rates of overweight/obese from 2012 to 2016. Under-weight recorded any time during pregnancy increased the risk of LBW (Table 3A, 3B). A third of this study population was underweight any time during pregnancy, which is an underestimation of pre-pregnancy BMI. More than a third of women were both anemic and underweight. The risk of stillbirths, neonatal deaths and LBW increased further if both anemia and underweight co-existed (Table 4). A limiting factor in our study was that we were not able to evaluate likely confounders of adverse outcomes in pregnancy such as socio-economic status, number and timing of ante-natal visits and use of tobacco, etc. as these data were not collected in the MNH registry.

The NFHS data (NFHS-3 and NFHS-4) found anemia prevalence rate of 54% between 2005 and 2016, with a minimal reduction in Maharashtra between NFHS-3(58%) to NFHS-4 (49%).[22] Disturbingly, our estimates were not only higher than the District Level Household Survey (DLHS-4, 2012-2013) which reported a rate between 43% and 49%among pregnant women aged 15-49 years in the Nagpur, Bhandara and Wardha districts, but also remained static over the past eight years [23-25]Previous studies from eastern Maharashtra have reported similar high rates of anemia. [26-28]The women in our study were young (20-29years), from both rural and backward tribal regions known for high prevalence of micronutrient deficiency specifically iron deficiency anemia and other causes of anemia such as sickle cell anemia, hookworm infestation and malaria.[29-31]Variations in rates of anemia may be due to methods used for surveying and assessing hemoglobin levels.[32] The unchanging status of anemia could be as a result of irregular and poor coverage of the national iron supplementation program [33-35] and suboptimal compliance with iron supplementation during pregnancy (<30%), [10,36] due to side effects, forgetfulness to take timely dosage of iron supplementation pills, and false beliefs about

For peer review only - http://bmjopen.bmj.com/site/about/guidelines.xhtml

the importance of anemia in pregnancy.[37-39] Directly observed administration of iron supplementation may improve compliance and is being implemented for adolescent girls in India.[40,41] We believe it should be considered in pregnant women. Some reduction in rates of severe anemia were observed which may be attributable to improved attendance of ante-natal care visits, early detection and current management guidelines using injectable iron for treatment of severe anemia at the public health facilities from the 12th Five year plan.[42]

Our study reports over a third of the study participants to be underweight during pregnancy, which is consistent with the findings of NFHS for rural Nagpur. [13] We also found an 8% increase in low BMI in pregnant womenbetween2009 and 2016. There was also a trend towards BMI from the normal to overweight/obese categories over this same time period as has been observed.[43]Maternal pre-pregnancy BMI as well as BMI during any trimester of pregnancy has different effects on maternal and fetal outcomes. Adverse effects of low BMI preferentially target the fetus, by causing growth restriction and LBW, whereas high BMI has a greater impact on maternal health and subsequently affects neonatal health through altered glucose homeostasis, leading to fetal macrosomia and other such pathophysiological mechanisms.[44-46]

Anemia and underweight co-existed among one-third of the study participants which increased over time. This change may be due to increasing prevalence of underweight as the rates of anemia did not change over the study period. The most probable cause for maternal underweight in this region would be malnutrition.[47,48] It is also likely that iron and other micronutrient deficiency were responsible for higher rates of anemia in underweight women[49-51] but this should be studied further to reduce rates of anemia in the community.

For peer review only - http://bmjopen.bmj.com/site/about/guidelines.xhtml

The rates of CS increased over the years consistent with an increase in facility based deliveries in India.[52] However, anemia at any level reduced the likelihood of CS and pregnancy related complication and underweight and anemic women are more likely to have fetal growth retardation and smaller babies.[53-56] Pregnancy related complication in our study population are most commonly due to obstructed labor and toxemia of pregnancy, both of which are less likely to occur in underweight and anemic women who tend to have smaller babies.[49] Women with higher BMI (who are less likely to be anemic) are at increased risk of miscarriage, gestational diabetes, preeclampsia, venous thromboembolism, induced labor, caesarean section, anesthetic complications and wound infections.[48]

The risk of LBW was mostly determined by low BMI and severity of anemia any time during pregnancy. The rates of LBW increased over the years in women who had moderate/severe anemia and in women who were underweight. Other Indian studies have reported similar results [9]. The risk of having LBW babies in women who were underweight also increased by 20%, consistent with other studies that have reported an association of maternal anthropometry and size of the baby.[2,3]A study in Tanzania, on 12,269 newborns, identified short maternal stature as an significant risk factor for SGA and preterm-appropriate for gestational age.[57] A recent study from Mexico reported that higher proportions of SGA neonates were born to malnourished and anemic mothers than the mothers who were either malnourished or anemic.[58]LBW babies are at increased risk of becoming stunted adults which will expound the inter-generational burden of LBW. [59]

For peer review only - http://bmjopen.bmj.com/site/about/guidelines.xhtml

The risk of stillbirths increased when any anemia was recorded after 20 weeks of GA. While there are studies to support our findings on the association of maternal anemia on stillbirths,[12,60,61] it is unclear whether it was as a result of an intrapartum complication or whether it reflected poor socio-economic conditions that limited access to quality intra-partum care. It is also possible that sickle cell anemia, hookworm infestation and malaria, all common in Eastern Maharashtra may have contributed to higher rates of stillbirths, [22-25] as does ethnicity in underweight women previously.[62]

The risk of neonatal deaths increased when mild anemia was recorded after 20 weeks of GA and if moderate/severe anemia was recorded at any time during pregnancy. Although there is a paucity of data on the reason for an association between anemia and neonatal mortality, it is consistent with the finding of a recent meta-analysis in LMIC (one study from India and another from Malawi)that found the odds of neonatal mortality to be 2.7 times higher among the anemic mothers. [55, 59, 63] The neonatal mortality could also be attributed to increased risk of preterm and LBW deliveries in women who are anemic [64] Underweight recorded any time during pregnancy did not increase neonatal death. However, when the two conditions co-existed, the risk was higher than presence of either one of them alone (table 4).Low maternal BMI and anemia are associated with LBW and preterm deliveries which are independent risk factors for infant mortality in India[65,66,67].

CONCLUSIONS AND PUBLIC HEALTH IMPLICATIONS

For peer review only - http://bmjopen.bmj.com/site/about/guidelines.xhtml

The combination of anemia and underweight in pregnancy increased the risk of stillbirths, neonatal deaths and delivering LBW babies. These findings raise major concerns because national programs to address iron deficiency anemia have not reduced the rates of anemia among the rural pregnant women in eastern Maharashtra over past 8 years. There has been a steady increase in prevalence of underweight in pregnancy with almost a third of pregnant women being both anemic and underweight. Immediate and effective public health interventions are needed to address maternal anemia and malnutrition to improve birth outcomes. There is an urgent need for evaluation and addressing of gaps of the iron supplementation program for adolescent girls and pregnant women in these areas with high rates of anemia, in addition to a more holistic approach to curb maternal malnutrition. Meanwhile, seeking simultaneously short-term strategies such as referral to a better healthcare service and management of delivery for the segment of pregnant population who are both anemic and underweight could be helpful in reducing neonatal mortality. Such actions are imperative to break the intergenerational cycle of poor growth in the offspring and also for improving child survival.

LIST OF ABBREVIATIONS

ANM - Auxiliary Nurse Midwife

APH - Antepartum Hemorrhage

BMI - Body Mass Index

CI - Confidence Interval

CS - Cesarean Section

For peer review only - http://bmjopen.bmj.com/site/about/guidelines.xhtml

DLHS - District Level Household Survey

GEE - Generalized Estimating Equations

GN - Global Network

IFA - Iron Folic Acid

IRB - Institutional Review Board

LBW - Low Birth Weight

LMIC - Low and Middle-Income Countries

LMRF - Lata Medical Research Foundation

MNHR - Maternal and Newborn Health Registry

MTP - Medical Termination of Pregnancy

NFHS - National Family and Health Survey

NICHD - *Eunice Kennedy Shriver* National Institute of Child Health and Human Development

PHC –Primary Health Center

PPH - Postpartum Hemorrhage

RA - Registry Administrator

RR - Risk Ratio

SGA - Small for Gestational Age

USA - United States of America

WHO - World Health Organization

AUTHOR CONTRIBUTIONS

For peer review only

AP conceived and designed the study, and developed the initial data collection tools; AP and AAP wrote the first draft of the manuscript, which PLH, PKD, YVP and SG subsequently revised. AP and AAP developed the analysis plan outline and AAP carried out the data analyses. AP, PLH, PKD, YVP and SG contributed to data analysis through review, interpretation and cross-checking figures and tables. All authors read, revised, and approved the final manuscript.

ACKNOWLEDGEMENTS

We are deeply indebted to Research Triangle International, Durham, USA for their enthusiastic support. Thanks and recognition are due to the Registry Administrators at the Nagpur site of the MNHR who collect data, often in very challenging circumstances, and the communities, families, and mothers who agree to participate in the MNHR. We express our sincere gratitude to Dr. Vaishali Khedikar, Dr. Savita Bhargav and Dr, Kunal Kurhe at LMRF, who helped immensely by strengthening the manuscript through their inputs.

DATA SHARING

For peer review only

A de-identified minimal dataset that underlies the findings and conclusions described in the manuscript can be shared upon request by the editors to verify the reported study findings. All authors hereby declare that individual participant data in any form is not a part of this publication.

FUNDING

This study was funded by the *Eunice Kennedy Shriver* National Institute of Child Health and Human Development of the US National Institutes of Health funded this study (Grants Reference Nos. U01HD058322 and U01HD078439).

ETHICS

The study in Nagpur, India has been reviewed and approved by the Institutional Review Board (IRB) of the Lata Medical Research Foundation (RPC#22A)and the Partners Human Research Committee/IRB in Boston, MA (H-35430).

CONSENT TO PARTICIPATE

For peer review only - http://bmjopen.bmj.com/site/about/guidelines.xhtml

The study was explained thoroughly and in detail, to the women who lived in the participating communities. The women were enrolled in the study only after giving informed consent. An IRB approved consent form was used for this purpose.

COMPETING INTERESTS

None declared

TRANSPARENCY

The lead author(ABP) affirms that the manuscript is an honest, accurate, and transparent account of the study being reported; that no important aspects of the study have been omitted; and that any discrepancies from the study as planned have been explained.

For peer review only - http://bmjopen.bmj.com/site/about/guidelines.xhtml

What is already known on this topic

Low body mass Index (BMI) and anemia during pregnancy have been independently identified as risk factors for delivering pre-term, small for gestational age (SGA) and LBWs, however their relationship with respect to neonatal mortality and stillbirth remains inconclusive. Also, their relationship of severity of anemia with adverse pregnancy outcomes is not well established for low middle-income countries (LMIC). The risk arising out of co-existence of anemia and low BMI -a common scenario in LMIC like India, on poor maternal has not been characterized.

What this study adds

This study showed a high prevalence of anemia (>90%) and underweight (35%) among rural pregnant women in eastern Maharashtra. The rate of anemia remained unaltered but there was an increase in the underweight and overweight/obese woman over the past 8 years from 2009-2016. This highlights the magnitude of persistent public health problem of nutrition origin despite the national programs in place and also the emerging dual burden of diseases in the rural areas as well.

Anemia and underweight during pregnancy were individually found to increase the risk of stillbirths, neonatal deaths and LBW. Women with moderate-severe anemia were at greater risk as compared to the mildly anemic women. These risks increased further if anemia and underweight were present together during pregnancy. This raises concerns as the two conditions often co-exist in LMICs and in the present study was, as high as, affecting a third of the study population.

Recommendations for policy

There is urgent need to address maternal malnutrition. Though holistic approach would be eradicating maternal malnutrition through more targeted and intensive food or medicinal supplement based approach, seeking short term strategies such as referral to a better healthcare service simultaneously could help in breaking the intergenerational cycle of poor growth in the offspring and reducing child mortality.

For peer review only - http://bmjopen.bmj.com/site/about/guidelines.xhtml

REFERENCES:

1. Black RE, Victora CG, Walker SP, et al. Maternal and child undernutrition and overweight in low-income and middle-income countries.*Lancet*2013; 382(9890): 427-51. doi: 10.1016/S0140-6736(13)60937-X. (accessed 30 November, 2017).
2. Bhutta ZA, Das JK, Rizvi A, et al. Evidence-based interventions for improvement of maternal and child nutrition: what can be done and at what cost? *Lancet* 2013; 382(9890): 452-77. doi:10.1016/S0140-6736(13)60996-4.(accessed 30 November, 2017).
3. Black RE, Allen LH, Bhutta ZA, et al. Maternal and child undernutrition: global and regional exposures and health consequences. *Lancet* 2008; 371(9608):243-60. doi: 10.1016/S0140-6736(07)61690-0.(accessed30 November, 2017).
4. United Nations Development Program.*Sustainable Development Goals*. http://www.undp.org/content/undp/en/home/sustainable-development-goals.html (accessed 30 November 2017).
5. World Health Organization. *Global nutrition targets 2025: policy brief series (WHO/NMH/NHD/14.2).2014*.http://apps.who.int/iris/bitstream/10665/149018/1/WHO_NMH_NHD_14.2_eng.pdf?ua=1(accessed on 11 Sept 2017).
6. Government of India. *Millennium Development Goals: India Country Report 2015*. http://mospi.nic.in/sites/default/files/publication_reports/mdg_2july15_1.pdf(accessed on 11 Sept 2017).
7. United Nations Inter-agency Group for Child Mortality. *Levels & Trends in Child Mortality: Report 2015*. https://www.unicef.org/publications/files/Child_Mortality_Report_2015_Web_9_Sept_15.pdf(accessed on 30 Nov 2017).
8. United Nations Children's Fund. *Committing to Child Survival: A promise renewed. Progress Report 2015*. http://www.apromiserenewed.org/wp-content/uploads/2015/09/APR_2015_8_Sep_15.pdf(accessed on 30 Nov 2017).
9. Van Der Linden EL, Browne JL, Vissers KM, et al. Maternal body mass index and adverse pregnancy outcomes: A Ghanaian cohort study. *Obesity* 2016; 24(1): 215-22. doi:10.1002/oby.21210. (accessed on 30 Nov 2017).

For peer review only - http://bmjopen.bmj.com/site/about/guidelines.xhtml

10. Patel P, Rupani M, Patel S. Antenatal care registration and predicting factors of late registration among pregnant women. 2013. Tropical Doctor 43(1):9-12
11. Pawalia A, Yadav V, Kulandaivelan S. Effect of Obesity on Pregnancy Outcomes – Indian Perspective: A Review. 2015. International Journal of Scientific Research Volume: 4(Issue: 7):453-457
12. NairM,ChoudhuryMK,Choudhury SS, et al. Association between maternal anemia and pregnancy outcomes: a cohort study in Assam, India. *BMJ Glob Health* 2016;1:e000026. doi:10.1136/bmjgh-2015-000026.(accessed30th November 2017).
13. International Institute of Population Sciences. *National Family Health Survey-4: Maharashtra State Fact Sheet*. http://rchiips.org/nfhs/pdf/NFHS4/MH_FactSheet.pdf (accessed 30 November 2017).
14. Haider BA, Olofin I, Wang M, et al.Anemia, prenatal iron use, and risk of adverse pregnancy outcomes: systematic review and meta-analysis.*BMJ* 2013;346:f3443. doi:10.1136/bmj.f3443. (accessed30 November 2017).
15. Goudar SS, Carlo WA, McClure EM, et al. The maternal and newborn health registry study of the global network for women's and children's health research. *Int J GynaecolObstet* 2012; 118(3): 190-3. doi:10.1016/j.ijgo.2012.04.022
16. World Health Organization. *Global database on body mass index.* Http://apps.who.int/bmi/index.jsp?introPage=intro_3.html (accessed 30 November 2017)
17. U.S.Agency for International Development/ PATH. *A Manual for Health Workers: Anemia Detection Methods in Low-Resource Settings*;1997. http://pdf.usaid.gov/pdf_docs/PNACC882.pdf(accessed30 November 2017).
18. World Health Organization (WHO*). Preventing and controlling anemia through primary health care: A guide for health administrators and program managers*. Geneva: WHO; 1989. http://www.who.int/nutrition/publications/micronutrients/anaemia_iron_deficiency/9241542497.pdf (accessed 30 November 2017).
19. Cuzick J. A Wilcoxon-type test for trend. *Stat Med* 1985;4(1):87–90.

For peer review only - http://bmjopen.bmj.com/site/about/guidelines.xhtml

20. Kumar J, Asha N, Murthy S, Sujatha MS, and Manjunath VG. Maternal Anemia in Various Trimesters and its Effect on Newborn Weight and Maturity: An Observational Study. Int J Prev Med. 2013 Feb; 4(2): 193–199.

21. Kumar A, Chaudhary K, Prasad S. Maternal Indicators and obstetric outcome in the north Indian population: A hospital based study. J Postgrad Med. 2010;56:192–5.

22. District Level Household survey 4 (2013-14) Available at: http://mospiold.nic.in/national_data_bank/dlhs4src_10feb2016.pdf Accessed on 12th May 2017

23. Arlappa N, Meshram II, BalakrishnaN,et al. Prevalence of anemia among different physiological groups in the rural areas of Maharashtra. *Indian J Community Health* 2014; 26(3):278-284.

24. International Institute for Population Sciences (IIPS) and Macro International. *National Family Health Survey (NFHS-3), 2005–06, India, Volume I*. Mumbai: IIPS;2007. http://rchiips.org/nfhs/NFHS-3%20Data/VOL-1/India_volume_I_corrected_17oct08.pdf(accessed 1 December 2017).

25. International Institute of Population Sciences(IIPS). *District Level Household and Facility Survey -4. State Fact Sheet Maharashtra (2012-13)*. http://rchiips.org/pdf/dlhs4/report/MH.pdf (accessed 22 November 2017).

26. Colah RB, Mukherjee MB, Martin S,et al. Sickle cell disease in tribal populations in India. *Indian J Med Res*2015;141(5):509-515. doi:10.4103/0971-5916.159492 (accessed 1 December 2017).

27. Corrêa G, Das M, KovelamudiR,et al. High burden of malaria and anemia among tribal pregnant women in a chronic conflict corridor in India. *Confl and Health 2017*; 11: 10. doi: 10.1186/s13031-017-0113-1 (accessed 1 December 2017).

28. Ahankari AS, Myles PR, Dixit JV,etal.Risk factors for maternal anemia and low birth weight in pregnant women living in rural India: a prospective cohort study. *Public Health* 2017 Oct;151:63-73. doi: 10.1016/j.puhe.2017.06.023(accessed 1 December 2017)

For peer review only - http://bmjopen.bmj.com/site/about/guidelines.xhtml

29. World Health Organization. *The prevalence of anemia in women: a tabulation of available information (WHO/MCH/MSM/92)*. 2nd ed. Geneva: World Health Organization; 1992. http://apps.who.int/iris/bitstream/10665/58994/1/WHO_MCH_MSM_92.2.pd (accessed 30 November 2017)

30. Bharati S, Pal M, Som S, Bharati P. Temporal trend of anemia among reproductive-aged women in India. *Asia Pac J Public Health*2015;27(2):NP1193-1207.

31. Balarajan YS, Fawzi WW, Subramanian SV. Changing patterns of social inequalities in anemia among women in India: cross-sectional study using nationally representative data. *BMJOpen* 2013;3(3):e002233 (accessed 1 December 2017).

32. Balasubramaniam P, Malathi A. Comparative study of hemoglobin estimated by Drabkin's and Sahli'smethods.*J Postgrad Med* 1992; 38(1):8-9.

33. Kalaivani K. Prevalence & consequences of anemia in pregnancy. *Indian J Med Res* 2009;130(5):627–33.

34. Pasricha SR, Biggs BA, Prashanth NS, et al. Factors influencing receipt of iron supplementation by young children and their mothers in rural India: local and national cross-sectional studies. *BMC Public Health*2011;11:617. doi: 10.1186/1471-2458-11-617(accessed 1 December 2017).

35. Chaturvedi S, RanadiveB.Are we really making motherhood safe? A study of provision of iron supplements and emergency obstetric care in rural Maharashtra.*Natl Med J India* 2007; 20(6):294-6.

36. Pal PP, Sharma S, SarkarTK,etal.Iron and folic acid consumption by the ante-natal mothers in a rural area of India in 2010. *IntJPrev Med* 2013;4(10):1213-1216.

37. Wadgave HV. Burden of anemia among the pregnant women in rural Area. Healthline2011;2:76-7.http://www.iapsmgc.org/index_pdf/53.pdf (accessed 1 December 2017).

38. Galloway R, Dusch E, Elder L,etal.Women's perceptions of iron deficiency and anemia prevention and control in eight developing countries.*SocSci Med* 2002;55(4):529-44.

39. Mithra P, Unnikrishnan B, Rekha T, et al. Compliance with iron-folic acid (IFA) therapy among pregnant women in an urban area of south India. *Afr Health Sci* 2013;13(4):880-885. 10.4314/ahs.v13i4.3(accessed 1 December 2017).

For peer review only - http://bmjopen.bmj.com/site/about/guidelines.xhtml

40. Kotecha PV, Nirupam S, Karkar PD. Adolescent girls' anemia control program, Gujarat, India. Indian J Med Res 2009;130(5):584-9.

41. Vir SC, Singh N, Nigam AK, et al. Weekly iron and folic acid supplementation with counseling reduces anemia in adolescent girls: A large-scale effectiveness study in Uttar Pradesh, India. *Food Nutr Bull*2008;29:186-94.

42. Bhatt B, Suryawanshi S, Mali B. "THE NATIONAL HEALTH OUTCOMES IN THE 12th FIVE YEAR PLAN AND THE GOALS THEREOF" International Journal of Application or Innovation in Engineering & Management (IJAIEM) 2014.l;3(3): 423-25

43. NCD Risk Factor Collaboration (NCD-RisC).Trends in adult body-mass index in 200 countries from 1975 to 2014: a pooled analysis of 1698 population-based measurement studies with 19.2 million participants. Lancet 2016;387(10026):1377-1396. doi: 10.1016/S0140-6736(16)30054-X(accessed 1 December 2017).

44. Raatikainen K, Heiskanen N, Heinonen S. Transition from overweight to obesity worsens pregnancy outcome in a BMI-dependent manner. Obesity (Silver Spring) 2006;14:165–171.

45. Ricart W, Lopez J, Mozas J, Pericot A, Sancho MA, Gonzalez N et al. Body mass index has a greater impact on pregnancy outcomes than gestational hyperglycaemia. Diabetologia. 2005;48:1736–1742.

46. Cedergren M. Effects of gestational weight gain and body mass index on obstetric outcome in Sweden. Int J Gynaecol Obstet. 2006;93:269–274.

47. National Nutrition Monitoring Bureau (NNMB). Diet and nutritional status of rural population, prevalence of hypertension and diabetes among adults and infant and young child feeding practices-A report of third repeat survey. Technical report no. 26.Hyderabad: National Institute of Nutrition, Indian Council of Medical Research; 2012. http://nnmbindia.org/1_NNMB_Third_Repeat_Rural_Survey___Technicl_Report_26.pdf (accessed 1 December 2017).

48. Mahanta LB, Choudhury M, Devi A, et al. On the Study of Pre-Pregnancy Body Mass Index (BMI) and Weight Gain as Indicators of Nutritional Status of Pregnant Women Belonging to Low Socio-Economic Category: A Study from Assam. *Indian J Community Med* 2015;40(3):198-202. doi: 10.4103/0970-0218.158870 (accessed 1 December 2017).

For peer review only - http://bmjopen.bmj.com/site/about/guidelines.xhtml

49. Sumarmi S, Puspitasari N, Handajani R, et al. Underweight as a risk factor for iron depletion and iron-deficient erythropoiesis among young women in rural areas of East Java, Indonesia.*Malays J Nutr* 2016;22(2):219–32.

50. Hettiarachchi M, Liyanage C, Wickremasinghe R, et al.Prevalence and severity of micronutrient deficiency: a cross-sectional study among adolescents in Sri Lanka.*Asia Pac J ClinNutr* 2006;15(1):56-63.

51. Darnton-Hill I, Mkparu UC. Micronutrients in Pregnancy in Low- and Middle-Income Countries. *Nutrients* 2015;7(3):1744-1768. doi:10.3390/nu7031744 (accessed on 1 December 2017).
https://link.springer.com/article/10.1186/s13690-017-0181-0

52. Patel AB, Prakash AA, Raynes-Greenow C, et al. Description of inter-institutional referrals after admission for labor and delivery: a prospective population based cohort study in rural Maharashtra, India.BMC Health Serv Res2017; 17(1):360. doi: 10.1186/s12913-017-2302-4 (accessed 1 December, 2017).

53. Ahankari A, Bapat S, Myles P, et al. Factors associated with preterm delivery and low birth weight: a study from rural Maharashtra, India. *F1000Res*2017;6:72. doi: 10.12688/f1000research.10659.1 1 (accessed 1 December 2017).

54. Rakesh PS. Prevalence of anemia in Kerala State, Southern India - a systematic review.*JClinDiagn Res* 2017;11(5):LE01-LE04. doi: 10.7860/JCDR/2017/24681.9951(accessed 1 December 2017).

55. UNICEF. *National Factsheet, Coverage Evaluation Survey, 2009*. New Delhi: UNICEF India Country Office; 2010. http://www.indiawaterportal.org/sites/indiawaterportal.org/files/National%20Factsheet_Coverage%20Evaluation%20Survey_UNICEF_2009.pdf (accessed 25 November 2017).

56. Sebastian T, Yadav B, Jeyaseelan L, et al. Small for gestational age births among South Indian women: temporal trend and risk factors from 1996 to 2010. *BMC Pregnancy and Childbirth*2015;15:7. doi:10.1186/s12884-015-0440-4 (accessed 1 December 2017).

57. Muhihi A, Sudfeld CR, Smith ER, et al. Risk factors for small-for-gestational-age and preterm births among 19,269 Tanzanian newborns. *BMC Pregnancy*

For peer review only - http://bmjopen.bmj.com/site/about/guidelines.xhtml

58. Kaur M, Chauhan A, Manzar MD, et al. Maternal anemia and neonatal outcome: aprospective study on urban pregnant women. *J ClinDiagn Res* 2015;9(12):QC04-QC08. doi:10.7860/JCDR/2015/14924.6985 (accessed 1 December 2017).

59. Varela-Silva MI, Azcorra H, Dickinson F, Bogin B, Frisancho AR. Influence of maternal stature, pregnancy age, and infant birth weight on growth during childhood in Yucatan, Mexico: a test of the intergenerational effects hypothesis. *Am J Hum Biol* 2009; 21(5):657-63. doi: 10.1002/ajhb.20883 (accessed 6 December 2017)

60. Haider BA, Olofin I, Wang M, et al.Anemia, prenatal iron use, and risk of adverse pregnancy outcomes: systematic review and meta-analysis. *The BMJ*2013;346:f3443. doi:10.1136/bmj.f3443(accessed 1 December 2017).

61. Mahajan SD, Singh S, Shah P, et al.Effect of maternal malnutrition and anemia on the endocrine regulation of fetal growth.*Endocr Res* 2004; 30(2):189-203.

62. Verma A, Shrimali L. Maternal body mass index and pregnancy outcome. J ClinDiagn Res 2012;6(9):1531-1533. doi:10.7860/JCDR/2012/4508.2551(accessed 1 December 2017).

*Childbirth*2016;16:110. doi:10.1186/s12884-016-0900-5(accessed 1 December 2017).

63. Tandu-UmbaB, Mbangama AM. Association of maternal anemia with other risk factors in occurrence of Great obstetrical syndromes at university clinics, Kinshasa, DR Congo. *BMC Pregnancy Childbirth* 2015; *15:* 183. doi: 10.1186/s12884-015-0623-z(accessed 1 December 2017).

64. Ramakrishnan U, Grant F, Goldenberg T, et al. Effect of women's nutrition before and during early pregnancy on maternal and infant outcomes: a systematic review.PaediatrPerinatEpidemiol, 26: 285–301. doi:10.1111/j.1365-3016.2012.01281.x (accessed 1 December 2017).

65. Kalk P, Guthmann F, Krause K, et al.Impact of maternal body mass index on neonatal outcome.*Eur J Med Res* 2009 May 14; 14(5): 216-22.

66. Nair M, Choudhury M, Choudhury S, Kakoty S, Sarma U, Webster P et al. Association between maternal anaemia and pregnancy outcomes: a cohort study in Assam, India. BMJ Global Health 2016; 1:e000026.doi:10.1136/bmjgh-2015-000026

67. Sankar MJ, Neogi SB, Sharma J, et al. State of newborn health in India. J Perinato 2016; 36(3), S3–S8. doi: 10.1038/jp.2016.183 (accessed 1 December 2017).

For peer review only - http://bmjopen.bmj.com/site/about/guidelines.xhtml

For peer review only

For peer review only - http://bmjopen.bmj.com/site/about/guidelines.xhtml

Figure 1: Categorical anemia (gm/dl)in included women by body mass index (kg/m^2), over year of enrolment

Figure1: Pic

For peer review only

For peer review only - http://bmjopen.bmj.com/site/about/guidelines.xhtml

Figure 2: Proportions of Anemia (gm/dl) & body mass index (kg/m^2) interaction in included women, over year of enrollment

Figure2: Pic

For peer review only

For peer review only - http://bmjopen.bmj.com/site/about/guidelines.xhtml

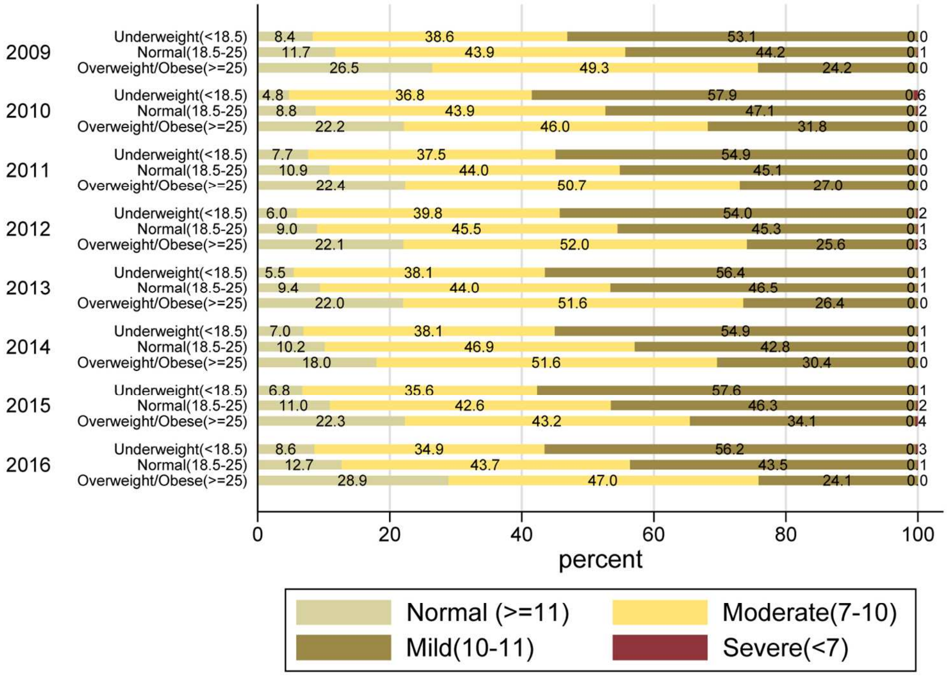

Figure 1: Categorical anemia (gm/dl)in included women by body mass index (kg/m2), over year of enrolment

139x101mm (300 x 300 DPI)

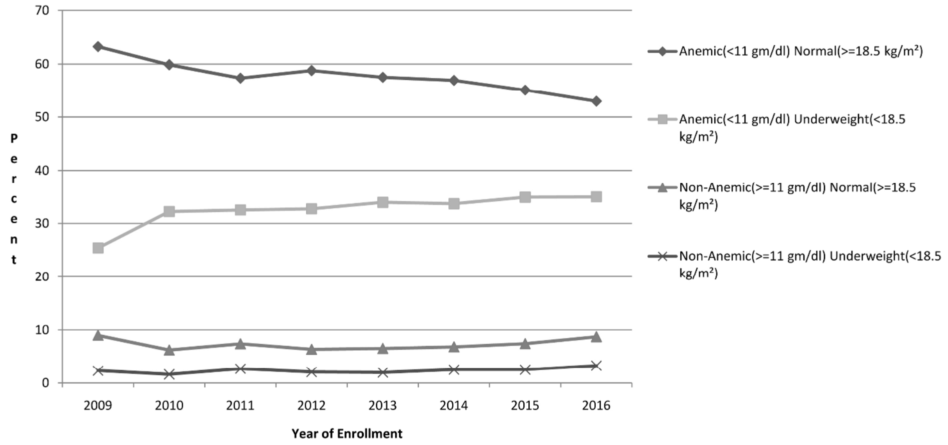

Proportions of Anemia (gm/dl) & body mass index (kg/m2) interaction in included women, over year of enrollment

247x114mm (300 x 300 DPI)

For peer review only

STROBE Statement—Checklist of items that should be included in reports of ***cohort studies***

	Item No	Recommendation
**Title and abstract**	1	(*a*) Indicate the study's design with a commonly used term in the title or the abstract ☑ - ***Page 1***
		(*b*) Provide in the abstract an informative and balanced summary of what was done and what was found ☑ - ***Page 5***
**Introduction**		
Background/rationale	2	Explain the scientific background and rationale for the investigation being reported ☑ - ***Page 8***
Objectives	3	State specific objectives, including any prespecified hypotheses ☑ - ***Page 8-9***
**Methods**		
Study design	4	Present key elements of study design early in the paper ☑ - ***Page 9***
Setting	5	Describe the setting, locations, and relevant dates, including periods of recruitment, exposure, follow-up, and data collection ☑ - ***Page 9***
Participants	6	(*a*) Give the eligibility criteria, and the sources and methods of selection of participants ☑ - ***Page 9-10***
		(*b*) For matched studies, give matching criteria and number of exposed and unexposed N/A
Variables	7	Clearly define all outcomes, exposures, predictors, potential confounders, and effect modifiers. Give diagnostic criteria, if applicable ☑ - ***Page 11 & 12***
Data sources/ measurement	8*	For each variable of interest, give sources of data and details of methods of assessment (measurement). Describe comparability of assessment methods if there is more than one group ☑ - ***Page 10 & 11***
Bias	9	Describe any efforts to address potential sources of bias ☑ - ***Page 10 & 11***
Study size	10	Explain how the study size was arrived at - N/A
Quantitative variables	11	Explain how quantitative variables were handled in the analyses. If applicable, describe which groupings were chosen and why ☑ - ***Page 10, 11 & 12***
Statistical methods	12	(*a*) Describe all statistical methods, including those used to control for confounding ☑ - ***Page 11 & 12***
		(*b*) Describe any methods used to examine subgroups and interactions ☑ - ***Page 12***
		(*c*) Explain how missing data were addressed ☑ - ***Page 12***
		(*d*) If applicable, describe analytical methods taking account of sampling strategy ☑ - ***Page 12***
		(*e*) Describe any sensitivity analyses - N/A
**Results**		
Participants	13*	(a) Report numbers of individuals at each stage of study—eg numbers potentially eligible, examined for eligibility, confirmed eligible, included in the study, completing follow-up, and analysed ☑ - ***Page 12 & 13***
		(b) Give reasons for non-participation at each stage - N/A
		(c) Consider use of a flow diagram - N/A
Descriptive data	14*	(a) Give characteristics of study participants (eg demographic, clinical, social) and information on exposures and potential confounders ☑ - ***Page 13***
		(b) Indicate number of participants with missing data for each variable of interest ☑ - ***Page 12 & 13***
Outcome data	15*	Report numbers of outcome events or summary measures ☑ - ***Page 15***
Main results	16	(*a*) Give unadjusted estimates and, if applicable, confounder-adjusted estimates and their

For peer review only - http://bmjopen.bmj.com/site/about/guidelines.xhtml

		precision (eg, 95% confidence interval). Make clear which confounders were adjusted for and why they were included ☑ - ***Page 17 thru 19***
		(*b*) Report category boundaries when continuous variables were categorized ☑ - ***Page 10 & 11***
		(*c*) If relevant, consider translating estimates of relative risk into absolute risk for a meaningful time period - N/A
Other analyses	17	Report other analyses done—eg analyses of subgroups and interactions, and sensitivity analyses ☑ - ***Page 17 & 19***
**Discussion**		
Key results	18	Summarise key results with reference to study objectives ☑ - ***Page 15 thru 19***
Limitations	19	Discuss limitations of the study, taking into account sources of potential bias or imprecision. Discuss both direction and magnitude of any potential bias ☑ - ***Page 7, 20-24***
Interpretation	20	Give a cautious overall interpretation of results considering objectives, limitations, multiplicity of analyses, results from similar studies, and other relevant evidence ☑ - ***Page 20 thru 24***
Generalisability	21	Discuss the generalisability (external validity) of the study results ☑ - ***Page 20 thru 24***
**Other information**		
Funding	22	Give the source of funding and the role of the funders for the present study and, if applicable, for the original study on which the present article is based ☑ - ***Page 27***

*Give information separately for exposed and unexposed groups.

Note: An Explanation and Elaboration article discusses each checklist item and gives methodological background and published examples of transparent reporting. The STROBE checklist is best used in conjunction with this article (freely available on the Web sites of PLoS Medicine at http://www.plosmedicine.org/, Annals of Internal Medicine at http://www.annals.org/, and Epidemiology at http://www.epidem.com/). Information on the STROBE Initiative is available at www.strobe-statement.org.

For peer review only

For peer review only - http://bmjopen.bmj.com/site/about/guidelines.xhtml
